# Interwrapping Distinct Metal-Organic Frameworks in Dual-MOFs for the Creation of Unique Composite Catalysts

**DOI:** 10.34133/2021/9835935

**Published:** 2021-07-09

**Authors:** Jia-Long Ling, Kai Chen, Chuan-De Wu

**Affiliations:** State Key Laboratory of Silicon Materials, Department of Chemistry, Zhejiang University, Hangzhou 310027, China

## Abstract

Incorporating metal nanoparticles (MNPs) inside metal-organic frameworks (MOFs) demonstrates superior catalytic properties in numerous reactions; however, the size and distribution of MNPs could not be well controlled, resulting in low product selectivity in catalysis by undergoing different catalytic reaction pathways. We report herein a facile strategy for integrating lattice-mismatched MOFs together to fabricate homogeneously distributed “dual-MOFs,” which are the ideal precursors for the preparation of MNPs@MOFs with unique catalytic properties. As a proof of concept, we successfully synthesize a dual-MOF HKUST-1/ZIF-8 for in situ creation of redox-active Cu NPs inside hierarchical porous ZIF-8 under controlled pyrolytic conditions. Combining the advantages of size-tunable Cu NPs in the molecular sieving matrix of ZIF-8, Cu@ZIF-8 demonstrates high activity and selectivity for transformation of alkynes into alkenes without overhydrogenation, which surpasses most of the catalysts in the literature. Therefore, this work paves a new pathway for developing highly efficient and selective heterogeneous catalysts to produce highly value-added chemicals.

## 1. Introduction

Metal-organic frameworks (MOFs) are a class of porous coordination materials, demonstrating unique catalytic properties by confining redox-active species inside the pores, providing special pore microenvironment for redox-active sites and sieving substrate and product molecules during catalysis [1–12]. MOFs have been extensively used as porous precursors to prepare MNPs@carbon (metal nanoparticles (MNPs)) composites under high annealing temperature or as porous hosts to support redox-active species for heterogeneous catalysis [13–15]. MNPs@carbon composites demonstrate superior catalytic properties in numerous reactions by providing conductive carbon matrices, which endowed charge separation and subsequent transfer more easily during catalysis. However, the pore structures of MOFs often severely collapsed under extreme thermolytic conditions, indicating that MNPs@carbon materials could not retain most of the original properties of MOFs [16, 17]. Incorporating redox-active MNPs into MOFs can retain the pore structures of MOFs and facilitate catalysis [18]. However, the size and distribution of MNPs could not be well controlled, often resulting in different catalytic reaction pathways and low product selectivity [19].

**Scheme 1:**
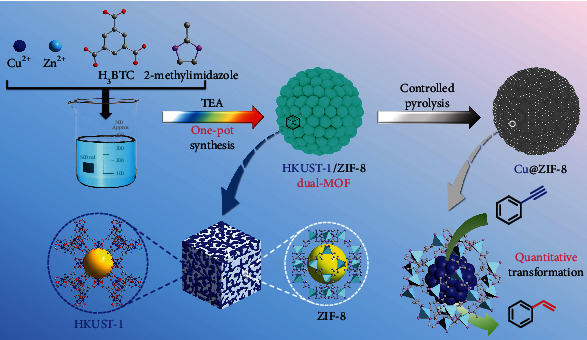
Schematic illustration of the synthesis procedures of dual-MOF HKUST-1/ZIF-8 and its derivative Cu@ZIF-8 composite for semihydrogenation of phenylacetylene into styrene. Controlled pyrolysis of dual-MOF HKUST-1/ZIF-8, consisting of lattice-mismatched MOFs HKUST-1 and ZIF-8 with distinct thermal stability, results in a composite material Cu@ZIF-8, consisting of in situ-generated Cu NPs inside the hierarchical pores of ZIF-8, which could prompt quantitative transformation of alkynes into alkenes without overhydrogenation.

To rationally tune the size, distribution, and microenvironment of redox-active MNPs for highly selective catalysis, we developed a “dual-MOF” strategy, in which one MOF is thermally labile to undergo controlled thermolysis for the formation of MNP active sites, while one MOF is thermally stable to act as porous support for control over the size and microenvironment of in situ-formed MNPs. Accordingly, precise control over the size, distribution, and microenvironment of MNPs in MOFs could be easily achieved by systematically regulating the distribution and ratio of different MOFs. As an illustrated example, we successfully synthesized a dual-MOF HKUST-1/ZIF-8, in which the high thermally stable MOF ZIF-8 acts as protective matrices to inhibit the migration of in situ-generated Cu NPs derived from thermolysis of the MOF HKUST-1 and further provides special pore microenvironment and molecular sieving function in catalysis, which could quantitatively transform alkynes into alkenes without overhydrogenation (Scheme 1).

## 2. Results

### 2.1. Synthesis of Lattice-Mismatched Dual-MOF HKUST-1/ZIF-8

There have been numerous lattice-matched MOFs with identical crystallographic parameters, while it is a challenge to integrate lattice-mismatched MOFs together by a conventional synthesis method, because the high interface energy caused by topology mismatch of different MOFs would generally lead to phases of MOF individuals [20, 21]. To address this challenge, we developed a coprecipitation strategy for the synthesis of lattice-mismatched dual-MOFs, consisting of homogeneously distributed MOF individuals. HKUST-1 and ZIF-8 are two well-known MOFs, which are built from paddle-wheel [Cu_2_(COO)_4_] secondary building units (SBUs) and 1,3,5-benzene-tricarboxylate (BTC) and tetrahedrally coordinated zinc cations connected by 2-methylimidazolate (MIM) linkers, respectively [22, 23]. It has been demonstrated that the formation enthalpies of the Cu-O bond in [Cu_2_(COO)_4_] SBUs and the Zn-N bond in Zn(MIM)_2_ are about −186 and −145 kJ/mol, which are higher than those of Zn-O and Cu-N bonds (about −180 and −90 kJ/mol), respectively [24, 25]. Therefore, nucleations of HKUST-1 and ZIF-8 are thermodynamically favourable, indicating that one-pot stoichiometric reaction of Cu(NO_3_)_2_·3H_2_O, Zn(NO_3_)_2_·6H_2_O and H_3_BTC in the presence of excess 2-methylimidazole would result in a dual-MOF composite, denoted as HKUST-1/ZIF-8.

### 2.2. Structural and Morphological Characterizations of Dual-MOF HKUST-1/ZIF-8

**Figure 1:**
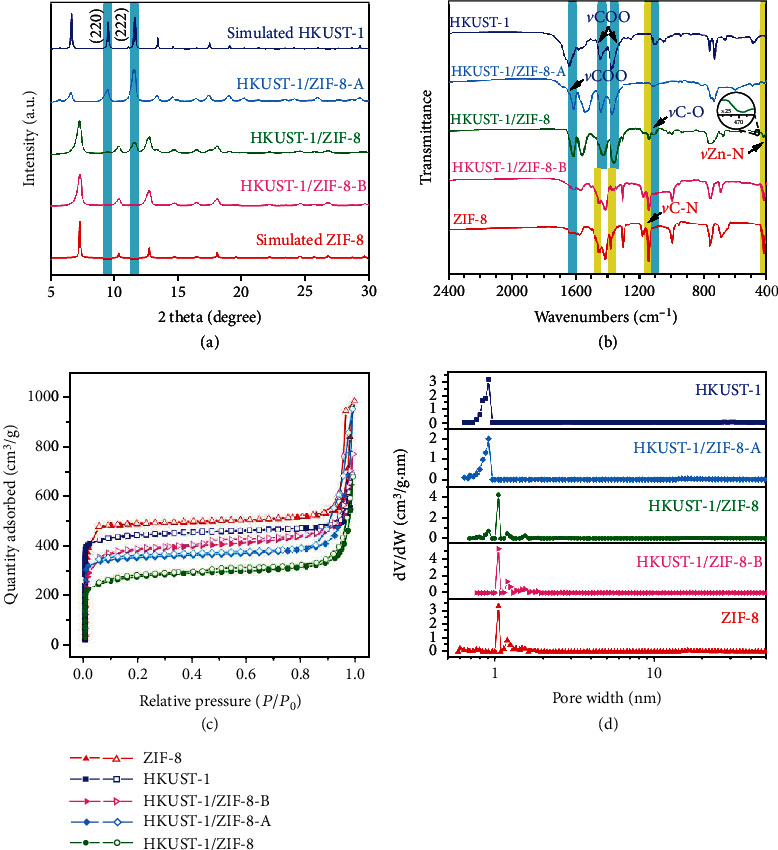
Characterizations of dual-MOF HKUST-1/ZIF-8: (a) PXRD patterns, (b) FT-IR spectra, (c) N_2_ adsorption/desorption isotherms, and (d) pore size distributions of HKUST-1/ZIF-8, HKUST-1/ZIF-8-A, and HKUST-1/ZIF-8-B.

The power X-ray diffraction (PXRD) pattern of HKUST-1/ZIF-8 clearly shows the character diffraction peaks ascribed to ZIF-8 and HKUST-1, including the overlapped peaks, while the diffraction intensity is variable depending on the contents of HKUST-1 and ZIF-8 (Figure 1(a), Figures S1 and S2). The individual HKUST-1 and ZIF-8 in the dual-MOF could be easily etched with acetic acid (HAc) or ammonium hydroxide (NH_3_·H_2_O) to result in hierarchical MOFs HKUST-1 and ZIF-8, based on their distinct acid and alkali stability, denoted as HKUST-1/ZIF-8-A and HKUST-1/ZIF-8-B, respectively. The PXRD peaks of HKUST-1/ZIF-8-A and HKUST-1/ZIF-8-B match well with those of HKUST-1 and ZIF-8, respectively, indicating successful production of dual-MOF HKUST-1/ZIF-8.

The FT-IR spectrum of HKUST-1/ZIF-8 shows the characteristic signals of HKUST-1 appearing at 1620, 1439, and 1363 cm^−1^ that are assigned to the COO–Cu_2_ stretching vibration, and the absorption band at 1110 cm^−1^ is ascribed to the C–O stretching vibration (Figure 1(b) and Figure S5) [26]. The peaks for ZIF-8 appear at 1148 cm^−1^ for the C–N stretching vibration and at 421 cm^−1^ for the Zn–N stretching vibration [27]. These results further confirmed the combination of HKUST-1 and ZIF-8 in the dual-MOF. There is also a very weak peak at 470 cm^−1^ ascribed to the Zn–O stretching vibration, which becomes evident when increasing the ratio of HKUST-1 to ZIF-8, indicating that HKUST-1 and ZIF-8 in HKUST-1/ZIF-8 are coordinatively interconnected in the dual-MOF (Figure S5). The FT-IR patterns of HKUST-1/ZIF-8-A and HKUST-1/ZIF-8-B match well with those of HKUST-1 and ZIF-8, respectively, demonstrating that the dual-MOF is majorly built from HKUST-1 and ZIF-8.

The textual characters of HKUST-1/ZIF-8 were studied by N_2_ sorption experiments (Figures 1(c) and 1(d), Figures S6 and S7, Table S1). HKUST-1/ZIF-8 exhibits the typical type IV isotherms, resulting in the total pore volume of 1.057 cm^3^/g and Brunauer-Emmett-Teller (BET) surface area of 968.3 m^2^/g, which are lower than those of HKUST-1 (*V*_pore_ = 1.068 cm^3^/g, *S*_BET_ = 1517.4 m^2^/g) and ZIF-8 (*V*_pore_ = 1.531 cm^3^/g, *S*_BET_ = 1670.7 m^2^/g), respectively. The surface area shrinkage may originate from interwrapping of the two MOFs. Nonlocal density functional theory (NLDFT) analysis results showed that the dominated pore sizes are ~9 and ~11 Å in HKUST-1/ZIF-8, which are ascribed to the pores in HKUST-1 and ZIF-8, respectively. In contrast, the etched samples of HKUST-1/ZIF-8-A and HKUST-1/ZIF-8-B exhibit the type IV isotherms with major pore sizes of ~9 and ~11 Å, respectively. The above results further proved that the dual-MOF is composed of HKUST-1 and ZIF-8.

**Figure 2:**
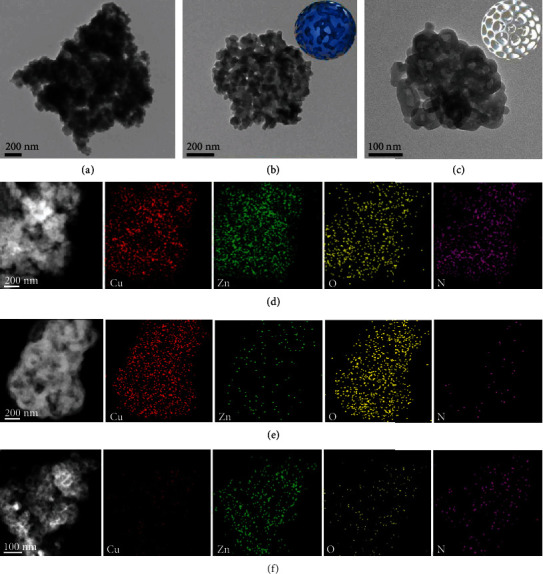
Morphology and composition of dual-MOF HKUST-1/ZIF-8. TEM images of (a) HKUST-1/ZIF-8, (b) HKUST-1/ZIF-8-A, and (c) HKUST-1/ZIF-8-B. HAADF-STEM and EDX elemental mapping images of (d) HKUST-1/ZIF-8, (e) HKUST-1/ZIF-8-A, and (f) HKUST-1/ZIF-8-B.

The morphology of HKUST-1/ZIF-8 was monitored by scanning electron microscopy (SEM) and transmission electron microscopy (TEM) (Figure 2 and Figures S8–S15). The SEM image of HKUST-1/ZIF-8 presents stacked irregular small particles with diameters of 40-60 nm, while the etched samples of HKUST-1/ZIF-8-A and HKUST-1/ZIF-8-B show the almost identical particle shape and sizes. TEM images clearly show the hierarchical porous structures in both etched samples, which originated from intertemplating between HKUST-1 and ZIF-8 in the dual-MOF. Energy-dispersive X-ray (EDX) spectroscopy elemental mapping images show that the Cu, Zn, O, and N elements in HKUST-1/ZIF-8 are uniformly distributed, indicating that HKUST-1 and ZIF-8 components are homogeneously distributed, which could be, respectively, etched under different conditions. The ratio of HKUST-1 to ZIF-8 is about 0.56 in the sample of HKUST-1/ZIF-8, as revealed by inductively coupled plasma optical emission spectroscopy (ICP-OES) and ^1^H NMR spectrum (Figure S16, Table S2).

### 2.3. Preparation and Characterizations of the Cu@ZIF-8 Composite

**Figure 3:**
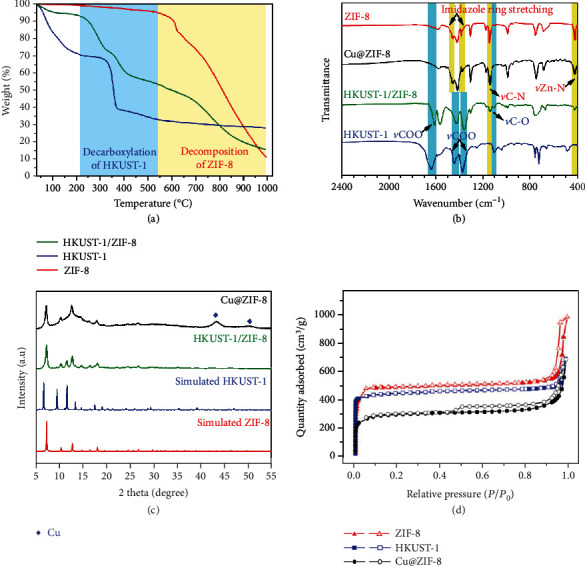
Characterizations of dual-MOF HKUST-1/ZIF-8 and its derivative Cu@ZIF-8 composite. (a) TG curves of HKUST-1/ZIF-8, HKUST-1, and ZIF-8. Comparisons of (b) FT-IR spectra and (c) PXRD patterns for HKUST-1/ZIF-8 and Cu@ZIF-8. (d) N_2_ adsorption/desorption isotherms of Cu@ZIF-8.

The thermal behaviors of HKUST-1/ZIF-8 were monitored by TG-MS measurement under N_2_ atmosphere (Figure 3(a) and Figures S17–S21). Upon raising the annealing temperature, a weight loss is observed in the temperature range of 200-450°C, which is similar to that of HKUST-1, indicating that the weight loss is derived from decarboxylation of the carboxylate linkers in HKUST-1 [28]. When the temperature was raised above 550°C, the TG curve sharply decayed, which is similar to the thermal behaviors of phase-pure ZIF-8 [29]. The thermal behaviors of the dual-MOF clearly demonstrate that the HKUST-1 component could be easily pyrolyzed while the ZIF-8 component remains intact under suitable pyrolytic conditions. We therefore pyrolyzed HKUST-1/ZIF-8 under N_2_ atmosphere at different temperatures for 2 h to fabricate Cu@ZIF-8 composites. The FT-IR spectrum indicates that the carboxylate moieties in HKUST-1 were almost completely decarboxylated for a solid sample of HKUST-1/ZIF-8 pyrolyzed at 350°C (Figure 3(b) and Figures S22–S24). In contrast, the characteristic ring stretching of 2-methylimidazolate ligands at 1460 and 1380 cm^−1^ became predominant, and the characteristic absorption peaks of C–N stretching vibration at 1148 cm^−1^ and Zn–N stretching vibration at 421 cm^−1^ remain intact, indicating that ZIF-8 is stable under the pyrolytic conditions. In the PXRD pattern, there appeared broad metallic Cu peaks (JCPDS, Card No. 04-0836, 43.3 and 50.4°) in the annealed sample of HKUST-1/ZIF-8, while the peaks ascribed to ZIF-8 retained, indicating successful decomposition of the HKUST-1 component, which was further confirmed by XPS spectra (Figure 3(c) and Figures S25–S28).

The textual characters of the 350°C treated sample of dual-MOF HKUST-1/ZIF-8 were analyzed by N_2_ sorption measurements (Figure 3(d), Figures S29–S31, Table S1). Cu@ZIF-8 exhibits the typical type IV isotherms with a BET surface area of 1014.1 m^2^/g. The relatively high nitrogen uptakes at low pressures were contributed by the extensive micropores, while the type H4 hysteresis loops at higher pressures suggest that there are mesopores in the composite material, indicating that Cu@ZIF-8 has hierarchical porous structure, which could facilitate mass transport during catalysis. The pore size distribution analysis revealed that the micropores in ZIF-8 were well reserved while those in HKUST-1 were wiped out in the annealed product, further certifying the decomposition of the HKUST-1 skeleton and retaining of the ZIF-8 skeleton under the pyrolytic conditions.

### 2.4. Tuning the Size of Cu NPs in the Cu@ZIF-8 Composite

**Figure 4:**
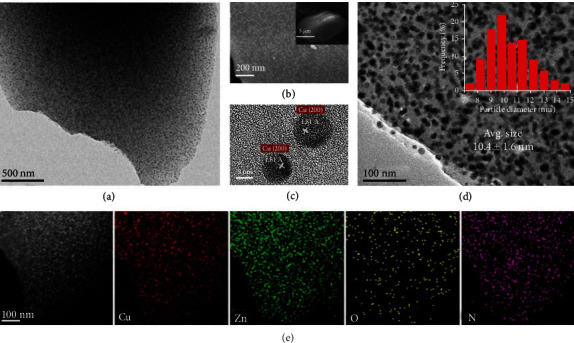
Morphology, composition, and texture of Cu@ZIF-8. (a) TEM and (b) SEM images of Cu@ZIF-8 (the inset shows a low-magnification SEM image). (c, d) HRTEM images of Cu@ZIF-8 with different resolutions (the inset histogram shows the size distribution of Cu metal NPs). (e) HAADF-STEM and EDX elemental mapping images of Cu@ZIF-8.

TEM and SEM images show that there are small aggregated NPs with average diameters of 10 ± 1.6 nm inside ZIF-8 (Figure 4 and Figures S32–S38). A high-resolution TEM (HRTEM) image shows that the lattice-fringe spacing of the NPs is 0.181 nm, ascribed to the metallic Cu (200) facet. Consistent with the conclusions from PXRD patterns, TEM images showed that the diameters of Cu NPs are dependent on the proportion of HKUST-1 and ZIF-8. Upon increasing the ZIF-8 content, the size of Cu NPs gradually decreases under the identical pyrolytic conditions, indicating that ZIF-8 in the dual-MOF plays important roles in tuning the sizes of Cu NPs by acting as physical barriers to prevent the aggregation of Cu NPs under annealing conditions (Table S3). EDX images of Cu@ZIF-8 shows that Cu, Zn, O, and N elements are uniformly distributed, indicating that the Cu NPs are well dispersed in hierarchical porous ZIF-8, which might serve as the homogeneous active sites in catalysis.

To investigate the roles of organic ligands in the formation of the HKUST-1/ZIF-8 dual-MOF and Cu@ZIF-8 composite, we studied the PXRD patterns of the pristine and annealed samples of HKUST-1/ZIF-8 with different proportions of organic ligands (Figures S39–S41). All pristine samples show the similar PXRD profile of HKUST-1/ZIF-8. Excess dosage of H_3_BTC would prevent the formation of ZIF-8, owing to the increased acidity. When the feed ratio of 2-methylimidazole to H_3_BTC was raised from 3 to 9, there appear the characteristic diffraction peaks ascribed to ZIF-8 and metallic Cu in the annealed samples. It is worth noting that the diffraction peaks of metallic Cu became broaden, upon increasing the content of 2-methylimidazole. By further increasing the dosage of 2-methylimidazole, no obvious diffraction peak could be assigned to the copper species, indicating the small sizes of in situ-formed Cu NPs. These experimental results revealed that the feed ratio of the two organic ligands plays crucial roles in the production of the dual-MOF and finally tuning the size of in situ-formed Cu NPs. These conclusions were further confirmed by FT-IR, TEM, ICP-OES, and ^1^H NMR (Figures S44–S63, Tables S2 and S3).

### 2.5. Catalytic Semihydrogenation of Alkynes

The annual world output of styrene to produce synthetic rubbers and plastics is more than 30 million tons, while the trace phenylacetylene would poison the polymerization catalysts and subsequently decrease the product quality [30, 31]. Therefore, removal of trace phenylacetylene from styrene feedstock is of high industrial importance [32]. Because there is no efficient way that could separate phenylacetylene from styrene in the industry, semihydrogenation represents the most practically applicable pathway to eliminate phenylacetylene from styrene [33]. Among numerous developed semihydrogenation catalysts, noble metal-based catalysts inherently exhibited high catalytic activity but low selectivity due to easy overhydrogenation, while earth abundant nonprecious metal-based catalysts showed high selectivity but intrinsic low catalytic activity [34–37]. For example, metallic Cu demonstrates high selectivity in semihydrogenation reaction, attributed to its excellent capability of easy desorption of the alkene species [38–40]; however, the catalytic activity of Cu-based catalysts is very low, while overhydrogenation still occurred [41].

**Figure 5:**
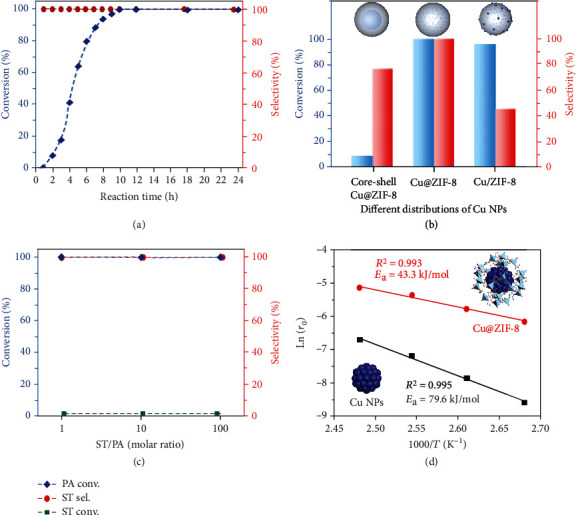
Catalytic semihydrogenation of phenylacetylene by Cu@ZIF-8. (a) Phenylacetylene conversion and styrene selectivity versus reaction time for phenylacetylene hydrogenation catalyzed by Cu@ZIF-8. (b) Phenylacetylene conversion and styrene selectivity versus different distributions of Cu NPs in ZIF-8. (c) Hydrogenation of a mixture of styrene (ST) and phenylacetylene (PA) catalyzed by Cu@ZIF-8. (d) Arrhenius plots for semihydrogenation of phenylacetylene catalyzed by Cu@ZIF-8 (red) and Cu@ZIF-8-A (black).

It is interesting that the annealed product Cu@ZIF-8 demonstrates excellent catalytic properties, which could quantitatively transform phenylacetylene into styrene (>99.9% conversion and >99.9% selectivity) under the catalytic conditions of 1.5 MPa H_2_ and 130°C (Figure 5(a), Figures S64–S69, Table S4). It is worth noting that no overhydrogenation occurred when prolonging the reaction time, even phenylacetylene was fully hydrogenated, which surpassed most of the literature work (Table S5). In contrast, the pristine ZIF-8, HKUST-1, and HKUST-1/ZIF-8 are almost inactive under the identical conditions (Table S4). The phenylacetylene conversion (9.0%) is dramatically decreased for the composite material derived from core-shell nHKUST-1@ZIF-8, because most of the Cu NPs are aggregated in the inner core with low dispersion (Figure 5(b), Figures S70–S72, Table S4) [42]. The excellent catalytic performance of Cu@ZIF-8 should be attributed to the homogeneous distribution and easy access of Cu NPs inside ZIF-8 with excellent molecular sieving properties and synergistic effect, which could highly improve the efficiency and selectivity in the catalysis [43]. In addition, it has been demonstrated that the structural heterogeneity would lead to low catalytic selectivity by undergoing different reaction pathways [44]. Therefore, the nature of homogenized active sites in Cu@ZIF-8 plays a vital role in improving the selectivity, attributed to their identical spatial and electronic interaction with reactant molecules. We also synthesized a composite Cu/ZIF-8 catalyst for comparison by a conventional solution impregnation method. Because there are substantial numbers of agglomerated Cu NPs on the outside of ZIF-8 with a broad size distribution, the styrene selectivity is very low (45.7%) (Figure 5(b), Figures S73–S75, Table S4). The catalytic properties are very sensitive to the Cu content in the composite materials, because the size of encapsulated Cu NPs is dependent on the Cu content, while the catalytic selectivity is related to the size of Cu NPs (Table S6). High Cu content resulted in low substrate conversion, while low Cu content led to overhydrogenation of styrene, due to the formation of large- and small-sized Cu NPs, respectively, revealing the structure-dependent characteristic of this catalytic reaction (Figure S76, Table S4) [36, 45].

To comprehensively understand the unique catalytic properties of Cu@ZIF-8 in the semihydrogenation reaction, the ZIF-8 component in Cu@ZIF-8 was digested in acetic acid (HAc) aqueous solution (Figures S77 and S78). The collected solid (denoted as Cu@ZIF-8-A) was used to catalyze the semihydrogenation reaction. Cu@ZIF-8-A exhibits relatively low activity (12.2% substrate conversion) under the same conditions, which was also observed for the annealed sample of HKUST-1 (26.2% conversion), indicating that the ZIF-8 matrix plays important roles in improving and stabilizing the catalytic efficiency of the encapsulated Cu NPs by confining and synergistic effect (Table S4) [46]. The ZIF-8 skeleton could be easily decomposed to form ZnO at 550°C (Figures S79–S82), which also resulted in decreased substrate conversion (85.6%) and styrene selectivity (48.1%) under the identical conditions, demonstrating that the microenvironment and molecular sieving effect offered by porous ZIF-8 are beneficial for improving the catalytic activity and selectivity (Table S4).

The very high selectivity catalyzed by Cu@ZIF-8 should also be ascribed to the unique molecular sieving function, which could selectively accumulate phenylacetylene molecules in the presence of styrene (Table S7). Cu@ZIF-8 could selectively absorb a large amount of phenylacetylene (3.46 mmol/g) from a solution of styrene and phenylacetylene in toluene (volume ratio of 1 : 1 : 100) at room temperature, while the adsorbed styrene is negligible. In contrast, the pristine HKUST-1/ZIF-8 exhibits low selectivity between phenylacetylene (3.58 mmol/g) and styrene (3.26 mmol/g). These results revealed that Cu@ZIF-8 could selectively enrich phenylacetylene and release styrene to improve the selectivity by avoiding overhydrogenation reaction during catalysis [38–40]. When Cu@ZIF-8 was used to hydrogenate styrene under the same catalytic conditions, no converted styrene could be detected, thus further proving its substrate specificity for phenylacetylene (Table S8). Cu@ZIF-8 was further evaluated in hydrogenation of a mixture of phenylacetylene and styrene (molar ratios of 1 : 1, 1 : 10, and 1 : 100), aimed at assessing its practical application for eliminating phenylacetylene from styrene (Figure 5(c), Table S9). Phenylacetylene is completely removed in the presence of excess styrene without occurring overhydrogenation, indicating that the molecular sieving function plays very important roles in the semihydrogenation reaction.

To understand the kinetic behaviors of Cu@ZIF-8 in the semihydrogenation reaction, we studied its catalytic properties at different temperatures. The semihydrogenation reaction catalyzed by Cu@ZIF-8 could be initiated at 80°C, which resulted in 20.1% styrene yield for 10 h and >99.9% styrene yield for 72 h, indicating that Cu@ZIF-8 is a highly active catalyst that could initiate the catalytic reaction under very mild conditions (Figure S83). The kinetic behaviors of Cu@ZIF-8 were investigated in the temperature ranges of 100-130°C by reducing the reaction time to 3 h, which would keep low conversions of phenylacetylene for the calculation of rational initial reaction rates (*r*_0_) (Figure 5(d)). The apparent activation energy (*E*_a_) for Cu@ZIF-8, calculated on the basis of the Arrhenius plot, is 43.3 kJ/mol, which is very close to 30–55 kJ/mol for Pd/C and much lower than that for Cu/Al_2_O_3_ [41, 47]. The low *E*_a_ value indicates that the special microenvironment provided by the porous ZIF-8 support would highly improve the hydrogenation activity of Cu NPs, which was proven by the high *E*_a_ value of Cu@ZIF-8-A (79.6 kJ/mol) without the porous ZIF-8 support. The highly improved catalytic activity of Cu@ZIF-8 could also be partially ascribed to the H_2_ trapping capability of the pores in ZIF-8, which serves as nanoreactors to speed up the hydrogenation process [48].

Cu@ZIF-8 also demonstrates high selectivity for semihydrogenation of a series of terminal alkynes (Figure 6). Ether, amine, pyridyl, halogen, and vinyl groups could be well tolerated, which resulted in the full conversion of the corresponding terminal alkynes with excellent selectivity of alkenes (>99.9%) (Figures S84–S103). It is worthy of note that Cu@ZIF-8 can also be extended to semihydrogenation of biorelated molecules, such as vitamin E, a class of biologically essential fat-soluble antioxidants. Vitamin E was traditionally synthesized from trimethylhydroquinone and isophytol, while isophytol was mainly derived from alkenols [49]. It is interesting that alkynol could be quantitatively semihydrogenated by Cu@ZIF-8 to yield the alkenol product (11) without overhydrogenation, showing great promise for practical applications.

X-ray photoelectron spectroscopy (XPS) was utilized to study the interfacial interaction between Cu NPs and ZIF-8 support (Figure S104). The Cu 2p_3/2_ binding energy for Cu^0^ species in Cu@ZIF-8 positively shifts to 933.0 eV, compared with the characteristic Cu 2p_3/2_ binding energy (932.6 eV) for Cu^0^ species without the ZIF-8 supporting matrix in pyrolyzed HKUST-1 at 350°C, while the binding energy of N 1s negatively shifts to 398.9 eV, compared with 399.4 eV for ZIF-8. The shift degree is also sensitive to the ratio of HKUST-1 to ZIF-8 in the dual-MOFs. These results indicate that there occurred electron transfer from encapsulated Cu NPs to the ZIF-8 supporting matrix, leading to positively charged Cu^*δ*+^ species located on the surface of Cu NPs inside the negatively charged ZIF-8 supporting matrix. The unique interfacial interaction between Cu NPs and ZIF-8 support would weaken its combination with styrene molecules to improve the styrene selectivity in catalysis, which has been confirmed by the selective sorption experiment between alkyne and alkene molecules (Table S7) [41, 45, 50].

**Scheme 2:**
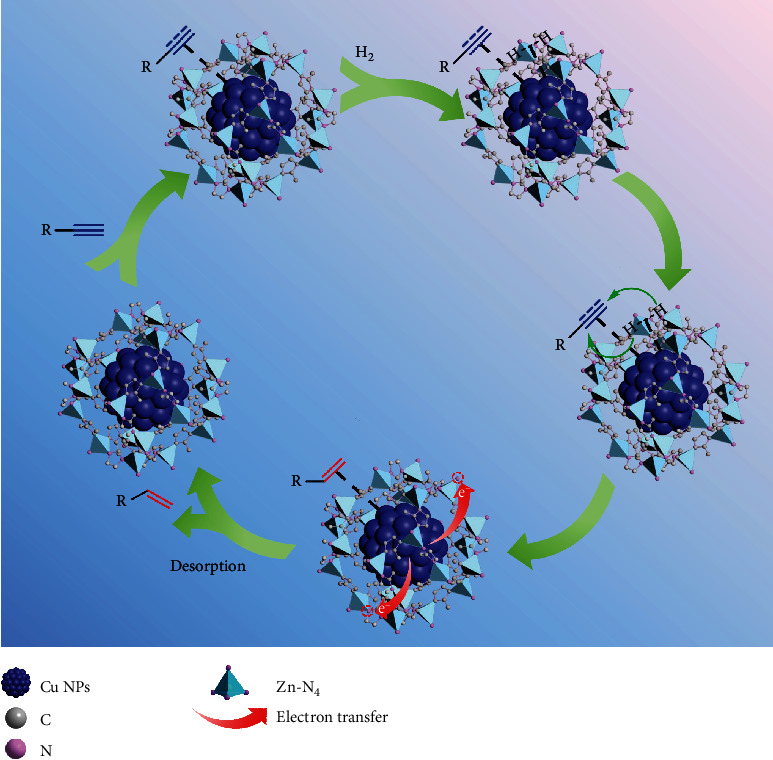
Proposed mechanism for semihydrogenation of terminal alkyne to produce terminal alkene over Cu@ZIF-8.

Based on the results obtained in this work and the literature, we proposed a possible catalytic pathway for semihydrogenation of terminal alkyne over Cu@ZIF-8 (Scheme 2) [37, 40, 51]. It has been known that terminal alkyne is firstly absorbed on the surface of the Cu active site to form an intermediate. Meanwhile, the Cu species in Cu@ZIF-8 is able to adsorb, activate, and dissociate molecular H_2_, which would react with the C≡C bond in alkyne. As a result, alkyne is converted into alkene, which is subsequently desorbed and released to avoid overhydrogenation of the alkene product on the surface of Cu NPs, owing to the weak sorption capability of positively charged Cu^*δ*+^ species located on the surface of Cu NPs to alkene moiety.

### 2.6. Recyclability of the Cu@ZIF-8 Catalyst

The composite catalyst Cu@ZIF-8 can be simply recovered by centrifugation and subsequently reused for catalyzing semihydrogenation of phenylacetylene with retained high catalytic efficiency and selectivity for six successive runs (Figure S105). The PXRD pattern and ICP-OES analysis results revealed that the catalyst is very stable (Figure S106, Table S3). The sharpened diffraction peaks of the recovered ZIF-8 after catalysis indicate that there occurred self-healing of the ZIF-8 skeleton under the catalytic conditions. The TEM image shows no obvious particle agglomeration in the recovered catalyst, demonstrating the important roles of the ZIF-8 matrix in stabilizing the Cu NPs by serving as the physical barriers (Figure S107). Compared with those reported catalysts, Cu@ZIF-8 exhibits high stability and unprecedented catalytic properties for quantitative transformation of alkynes into alkenes, which is able to simplify the postprocesses and save energy to meet the standard of practical applications (Table S5).

## 3. Summary

In summary, we established a facile strategy for incorporating lattice-mismatched MOFs to fabricate hybrid “dual-MOFs,” consisting of homogeneously distributed different MOFs with distinct thermal stability, which could be partially pyrolyzed to prepare MNPs@MOF composite materials under controlled annealing conditions. The size, distribution, and microenvironment of encapsulated Cu NPs in Cu@ZIF-8 can be well controlled to improve the catalytic performance. By combining the advantages of size-tunable Cu NPs and excellent molecular sieving ZIF-8, Cu@ZIF-8 could prompt quantitative transformation of terminal alkynes into alkenes without overhydrogenation. Therefore, this work provides a generally applicable strategy for control over homogeneous distribution of different MOFs in dual-MOFs, which should open up a new pathway for rational preparation of high-performance catalysts with unique catalytic properties.

## 4. Materials and Methods

### 4.1. Materials

All reagents and solvents were commercially available, which were directly used without purification, unless otherwise indicated. Zinc(II) nitrate hexahydrate (Zn(NO_3_)_2_·6H_2_O) (99%), copper(II) nitrate trihydrate (Cu(NO_3_)_2_·3H_2_O) (99%), acetone (≥99.5%), isopropanol (≥99.7%), methanol (≥99.5%), ethanol (≥99.7%), triethylamine (TEA) (≥99.5%), toluene (≥99.5%), *N*,*N*-dimethylformamide (DMF) (≥99.5%), ammonium hydroxide (NH_3_·H_2_O) (25–28%), and acetic acid (HAc) (≥99.8%) were purchased from Sinopharm. 1,3,5-Benzenetricarboxylic acid (H_3_BTC) (98%), styrene (99.5%), phenylacetylene (98%), and 2-methylimidazole (98%) were obtained from Energy Chemical.

### 4.2. Characterization

Powder X-ray diffraction (PXRD) patterns were collected on a RIGAKU D/MAX 2550/PC for Cu-K*α* radiation (*λ* = 1.5406 Å) in the 2*θ* range of 5–55° with an exposure time of 1 s and step size of 0.02°. Thermogravimetric-mass spectrometry (TG-MS) experiments were performed on a TGA/DSC1 1100S (Mettler Toledo) coupled with a ThermoStar GSD320 mass spectrometry (Pfeiffer Vacuum) under 1 atm N_2_ atmosphere. ^1^H NMR spectra were collected on a 400 MHz Bruker spectrometer. Fourier transform infrared (FT-IR) spectra were recorded from KBr pellets on a Nicolet NEXUS 470 spectrometer in the range of 400–4000 cm^−1^. X-ray Auger electron spectroscopy (XAES) and X-ray photoelectron spectroscopy (XPS) were measured on a Thermo ESCALAB 250Xi with Al-K*α* irradiation (1486.6 eV), and the C1s peak at 284.8 eV was used to calibrate the binding energies. The morphology of the composite materials was studied by field emission scanning electron microscopy (FESEM) on a Hitachi SU-8010, transmission electron microscopy (TEM) on a Hitachi HT-7700, and high-resolution TEM (HRTEM) on a JEOL 2100F system equipped with energy-dispersive X-ray (EDX) spectroscopy. The N_2_ adsorption/desorption isotherms were performed on a Micromeritics ASAP 2020 surface area analyzer. The solid samples were degassed in vacuum at 80°C for 6 h prior to analysis. Copper and zinc contents were determined by using a Varian 730-ES ICP-OES Spectrometer. The catalytic results for semihydrogenation of phenylacetylene were analyzed by GC (FULI, 9790 II) equipped with an FID detector and a PEG-20M capillary column using N_2_ as carrier gas. GC-MS spectra were recorded on a SHIMADZU GCMS-QP 2010.

### 4.3. Preparation of the Dual-MOF HKUST-1/ZIF-8

Cu(NO_3_)_2_·3H_2_O (1.21 g, 5.0 mmol) and Zn(NO_3_)_2_·6H_2_O (1.49 g, 5.0 mmol) were added into 10 mL methanol under stirring, resulting in a homogeneous blue solution. 2-Methylimidazole (1.64 g, 20.0 mmol) in 5 mL methanol was subsequently added into the above solution under stirring at room temperature, which was further reacted for 10 min to form a homogeneous dark blue solution. A solution of H_3_BTC (700 mg, 3.33 mmol) and TEA (3.0 mL) in 10 mL methanol was added into the above solution at room temperature, which was further reacted for 3 h under stirring. The green solid of HKUST-1/ZIF-8 was collected by centrifuging and washing with methanol several times, which was subsequently activated at 80°C in an oven overnight.

### 4.4. Preparation of Etched Samples of the Dual-MOF HKUST-1/ZIF-8

In a typical experimental procedure, 200 mg activated HKUST-1/ZIF-8 and HAc (0.5 mL) were added into 5 mL methanol, which was stirred for 30 min at room temperature. The blue solid was collected by centrifuging, washing with methanol several times, and drying at 80°C overnight, denoted as HKUST-1/ZIF-8-A, where A represents the acetic acid.

200 mg activated HKUST-1/ZIF-8 and NH_3_·H_2_O (0.5 mL) were immersed in 5 mL methanol, which was stirred for 30 min at room temperature. The white solid was collected by centrifuging, washing with methanol several times, and drying at 80°C overnight, denoted as HKUST-1/ZIF-8-B, where B represents the ammonium hydroxide.

### 4.5. Synthesis of the Cu@ZIF-8 Composite

In a typical experimental procedure, 200 mg activated HKUST-1/ZIF-8 solid sample was put in a furnace, which was pyrolyzed at 350°C for 2 h under continuous nitrogen flow. The annealed product was denoted as Cu@ZIF-8.

### 4.6. Preparation of the Etched Sample of the Cu@ZIF-8 Composite

200 mg Cu@ZIF-8 and HAc (0.5 mL) were added into 5 mL methanol, and the mixture was reacted for 30 min under stirring at room temperature. The solid was collected by centrifugation and washed with methanol several times, which was dispersed in 2 mL toluene for the catalytic test, denoted as Cu@ZIF-8-A, where A represents the acetic acid.

### 4.7. Semihydrogenation of Alkynes

Semihydrogenation of alkynes was performed in a 50 mL stainless high-pressure autoclave. Alkyne (0.2 mmol), solvent (2 mL), and catalyst (10 mol% based on Cu) were loaded to the autoclave, which was sealed, flushed five times with H_2_ at 1 MPa, and subsequently pressurized with H_2_ to the desired pressure. The reaction mixture was heated to the targeted temperature, reacted at the temperature for a certain time, and cooled down to room temperature. The reaction mixture was centrifuged, and the liquid phase was subjected to GC-MS analysis by using DMF as an internal standard.

## Figures and Tables

**Figure 6:**
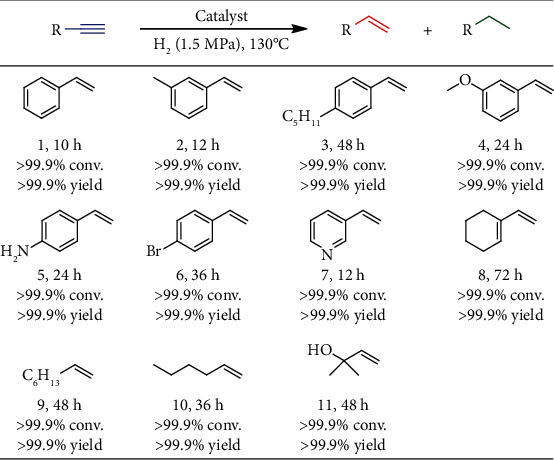
Semihydrogenation of various terminal alkynes catalyzed by Cu@ZIF-8. Reaction conditions: 0.2 mmol alkyne, Cu@ZIF-8 (10 mol% based on Cu), 2 mL toluene, 1.5 MPa H_2_, 130°C, and 10 h. Conversions and yields were calculated on the basis of GC-MS analysis results.

## Data Availability

All data needed to evaluate the conclusions in the paper are present in the paper and Supplementary Materials. Additional data which are related to this paper may be requested from the authors.

## Supplementary Materials

Supplementary Materials This includes details of synthesis and characterizations, PXRD patterns, NMR spectra, TGA curves, N_2_ adsorption/desorption isotherms, FT-IR spectra, SEM and TEM images, EDX element mapping images, XPS spectra, GC-MS spectra, and catalytic tests. Correspondence and requests for materials should be addressed to C.D.W. (cdwu@zju.edu.cn).
