# Supplementary material for: Interwrapping Distinct Metal-Organic Frameworks in Dual-MOFs for the Creation of Unique Composite Catalysts
Source: Research (Wash D C). 2021 Jul 9;2021:9835935. doi: 10.34133/2021/9835935 (PMC8286356; doi:10.34133/2021/9835935)
Supplement: Supplementary Materials — This includes details of synthesis and characterizations, PXRD patterns, NMR spectra, TGA curves, N2 adsorption/desorption isotherms, FT-IR spectra, SEM and TEM images, EDX element mapping images, XPS spectra, GC-MS spectra, and catalytic tests. Correspondence and requests for materials should be addressed to C.D.W. (cdwu@zju.edu.cn). [file 9835935.f1.docx]

Supplementary Materials

**Inter-Wrapping Distinct Metal-Organic Frameworks in Dual-MOFs for the Creation of Unique Composite Catalysts**

Jia-Long Ling, Kai Chen and Chuan-De Wu*

*State Key Laboratory of Silicon Materials, Department of Chemistry, Zhejiang University, Hangzhou, 310027, P. R. China*

## Supplementary methods

### Synthesis of HKUST-1

Cu(NO_3_)_2_·3H_2_O (1.81 g, 7.5 mmol) was dissolved in 10 mL methanol to form a homogeneous blue solution. A solution of H_3_BTC (1.05 g, 5.0 mmol) and TEA (1 mL) in 10 mL methanol was added into the above solution, which was stirred at room temperature for 3 h. Blue solid of HKUST-1 was collected by centrifugation, washed with methanol three times, and activated at 80 ^o^C overnight in an oven.

### Synthesis of ZIF-8

Zn(NO_3_)_2_·6H_2_O (0.74 g, 2.5 mmol) was dissolved in 10 mL methanol to form a homogeneous solution. A solution of 2-methylimidazole (0.82 g, 10.0 mmol) in 10 mL methanol was added into the above solution, which was stirred at room temperature for 3 h. White solid of ZIF-8 was collected by centrifugation, washed with methanol three times, and activated at 80 ^o^C overnight in an oven.

### Synthesis of nHKUST-1@ZIF-8

nHKUST-1@ZIF-8 was synthesized according to the literature [S1]. Cu(NO_3_)_2_·3H_2_O (0.435 g, 1.8 mmol), HAc (0.62 mL) and TEA (0.50 mL) were added into 12 mL ethanol under stirring for 1 h. H_3_BTC (0.210 g, 1.0 mmol) was added into the above solution, which was stirred for 2 h to form a homogeneous solution. The above solution was transferred into a Teflon-lined autoclave, and further reacted at room temperature for 24 h. Blue solid of nHKUST-1 was collected by centrifugation and washed with methanol three times, which was dispersed in 25 mL methanol. The above nHKUST-1 suspension (5 mL) was diluted to 50 mL with methanol. A solution of PVP (0.350 g) in 10 mL methanol was added into the above solution, which was stirred at room temperature for 12 h. The PVP-stabilized nHKUST-1 was collected by centrifugation, washed with methanol three times, and dispersed in 5 mL methanol. The PVP-stabilized nHKUST-1 suspension (1 mL) and Zn(NO_3_)_2_·6H_2_O (0.372 g, 1.25 mmol) were dispersed in 50 mL methanol. A solution of 2-methylimidazole (0.103 g, 1.25 mmol) in 50 mL methanol was added into the above solution, which was reacted at room temperature for 24 h. Blue solid of nHKUST-1@ZIF-8 was collected by centrifugation, washed with methanol three times, and activated at 80 ^o^C in an oven overnight.

### Synthesis of Cu-doped ZIF-8

Cu-doped ZIF-8 was synthesized according to the literature with a slight modification [S2]. Activated ZIF-8 (0.60 g) was added into 60 mL methanol under ultrasonication conditions. Cu(NO_3_)_2_·3H_2_O (0.60 g, 2.5 mmol) was added into the above ZIF-8 suspension, which was stirred at room temperature for 3 h. Blue solid of Cu-doped ZIF-8 was collected by centrifugation, washed with methanol three times, and activated by drying at 80 ^o^C in an oven overnight.

### Preparation of nHKUST-1@ZIF-8 derived core-shell Cu@ZIF-8 composite

In a typical experimental procedure, 200 mg activated nHKUST-1@ZIF-8 was placed in a furnace and heated to 350 ^o^C with a ramping rate of 5 ^o^C/min, and maintained at the destination temperature for 2 h under continuous nitrogen flow. The annealed product was denoted as nHKUST-1@ZIF-8-350.

### Preparation of Cu-doped ZIF-8 derived Cu/ZIF-8 composite

In a typical experimental procedure, 200 mg activated Cu-doped ZIF-8 was placed in a furnace and heated to 350 ^o^C with a ramping rate of 5 ^o^C/min, and maintained at the destination temperature for 2 h under continuous nitrogen flow. The annealed product was denoted as Cu/ZIF-8.

### Catalytic tests

### A typical procedure for semihydrogenation of terminal alkynes

Catalytic experiments were carried out in a 50 mL stainless high-pressure autoclave equipped with a magnetic stirrer. For a typical reaction, alkyne (0.2 mmol), 2 mL solvent and catalyst (10 mol% based on Cu) were loaded to the reactor. The sealed reactor was flushed five times with H_2_ at 1 MPa, then pressurized with H_2_ to the desired pressure, which was heated to the targeted temperature, and maintained at the temperature for a certain reaction time. After catalytic reaction, the autoclave was cooled down to room temperature. The liquid phase was separated from the reaction mixture by centrifugation. The identity of products was determined by GC-MS, and compared with the authentic samples, while the conversion and selectivity were determined by gas chromatography (GC) with a flammable ionized detector (FID) using DMF as an internal standard. The conversion, selectivity and product yield were estimated by the following formulae:

| Conversion of alkyne = (1 − | | moles of alkyne after reaction | | | ) × 100% | |
| --- | --- | --- | --- | --- | --- | --- |
|  |  | moles of alkyne added | | |  |  |
| Yield of alkene = | | | moles of alkene yield | × 100% | | |
|  |  |  | moles of alkyne added |  |  |  |
| Selectivity of alkene = | moles of alkene yield | | | | | × 100% |
|  | moles of alkyne added − moles of alkyne after catalysis | | | | |  |

### A typical procedure for semihydrogenation of styrene

A mixture of styrene (22 μL, 0.2 mmol), toluene (2 mL) and Cu@ZIF-8 (10 mol% based on Cu) was stirred at 130 ^o^C under 1.5 MPa H_2_ for 10 h, and centrifugated. The supernatant liquid was subjected to GC analysis and compared with the authentic samples analyzed under the same conditions.

### A typical procedure for hydrogenation of the mixture of phenylacetylene and styrene by Cu@ZIF-8

A mixture of phenylacetylene (22 μL, 0.2 mmol), styrene (22 μL, 0.2 mmol), toluene (2 mL) and Cu@ZIF-8 (10 mol% based on Cu) was stirred at 130 ^o^C under 1.5 MPa H_2_ for 10 h, and centrifugated. The supernatant liquid was subjected to GC analysis and compared with the authentic samples analyzed under the same conditions.

### A typical procedure for adsorption of phenylacetylene and styrene

An activated sample of Cu@ZIF-8 (10 mg) was stirred in 2 mL toluene containing phenylacetylene (22 μL, 0.2 mmol) and styrene (22 μL, 0.2 mmol) at room temperature under N_2_ atmosphere for 10 h, and centrifugated. The supernatant liquid was subjected to GC analysis and compared with the authentic samples analyzed under the same conditions.

### Kinetic study experiment procedures for semihydrogenation of phenylacetylene catalyzed by Cu@ZIF-8

A mixture of phenylacetylene (22 μL, 0.2 mmol), toluene (2 mL) and Cu@ZIF-8 (10 mol% based on Cu) was stirred at 100–130 ^o^C under 1.5 MPa H_2_ for 3 h, and centrifugated. The supernatant liquid was subjected to GC analysis and compared with the authentic samples analyzed under the same conditions.

### Kinetic study experiment procedures for semihydrogenation of phenylacetylene catalyzed by Cu@ZIF-8-A

A mixture of phenylacetylene (22 μL, 0.2 mmol), toluene (2 mL) and Cu@ZIF-8-A (10 mol% based on Cu) was stirred at 100–130 ^o^C under 1.5 MPa H_2_ for 10 h, and centrifugated. The supernatant liquid was subjected to GC analysis and compared with the authentic samples analyzed under the same conditions.

### Recycling experimental procedures for semihydrogenation of phenylacetylene catalyzed by Cu@ZIF-8

A mixture of phenylacetylene (22 μL, 0.2 mmol), toluene (2 mL) and Cu@ZIF-8 (20 mol% based on Cu) was stirred at 130 ^o^C under 1.5 MPa H_2_ for 10 h. The catalyst was recovered by centrifugation and washed with methanol three times. The recovered catalyst was reused in the successive run. The supernatant was analyzed by GC to determine the conversion, selectivity and product yield.

**
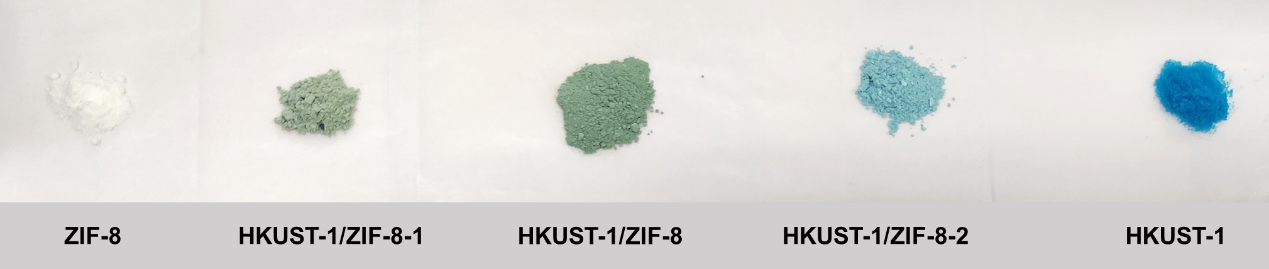
**

**Figure S1.** Photographs of ZIF-8, HKUST-1/ZIF-8-1, HKUST-1/ZIF-8, HKUST-1/ZIF-8-2 and HKUST-1. The dual-MOFs with different contents of HKUST-1 and ZIF-8 were synthesized by the same procedure of HKUST-1/ZIF-8. The ratios of HKUST-1 to ZIF-8 were quantified to be 0.25, 0.56 and 2.77 in the samples of HKUST-1/ZIF-8-1, HKUST-1/ZIF-8 and HKUST-1/ZIF-8-2, respectively, as revealed by ICP-OES and ^1^H NMR spectra.


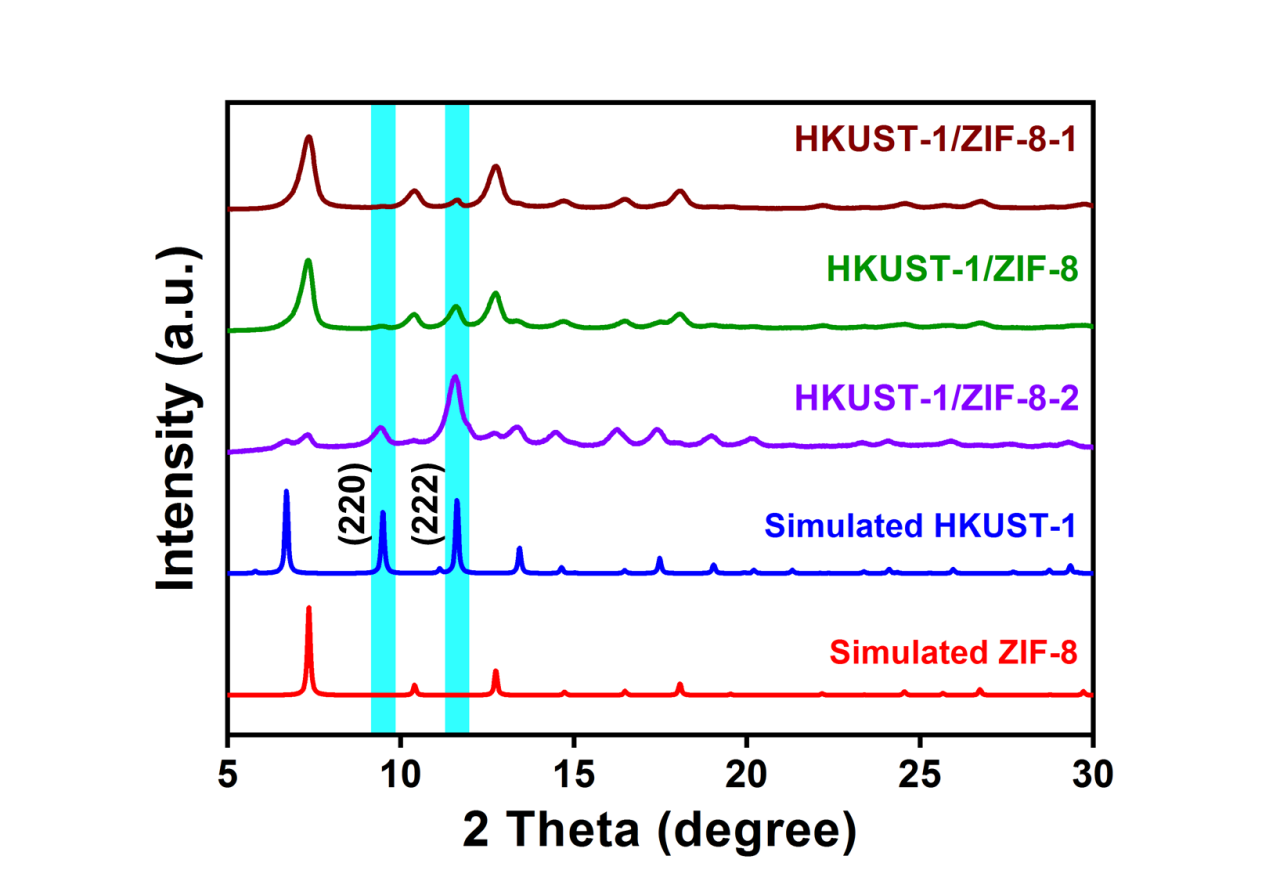


**Figure S2.** Comparison of the PXRD patterns of HKUST-1/ZIF-8, HKUST-1/ZIF-8-1 and HKUST-1/ZIF-8-2. The samples of HKUST-1/ZIF-8 and HKUST-1/ZIF-8-1 mainly show the structural features of ZIF-8, and there are only two obvious diffraction peaks ((220) and (222)) ascribed to HKUST-1, due to the low content of HKUST-1 and peak overlapping. Upon increasing the HKUST-1 content in HKUST-1/ZIF-8-2, the diffraction peaks for HKUST-1 became predominant, along with weakened diffraction signals for ZIF-8.

**
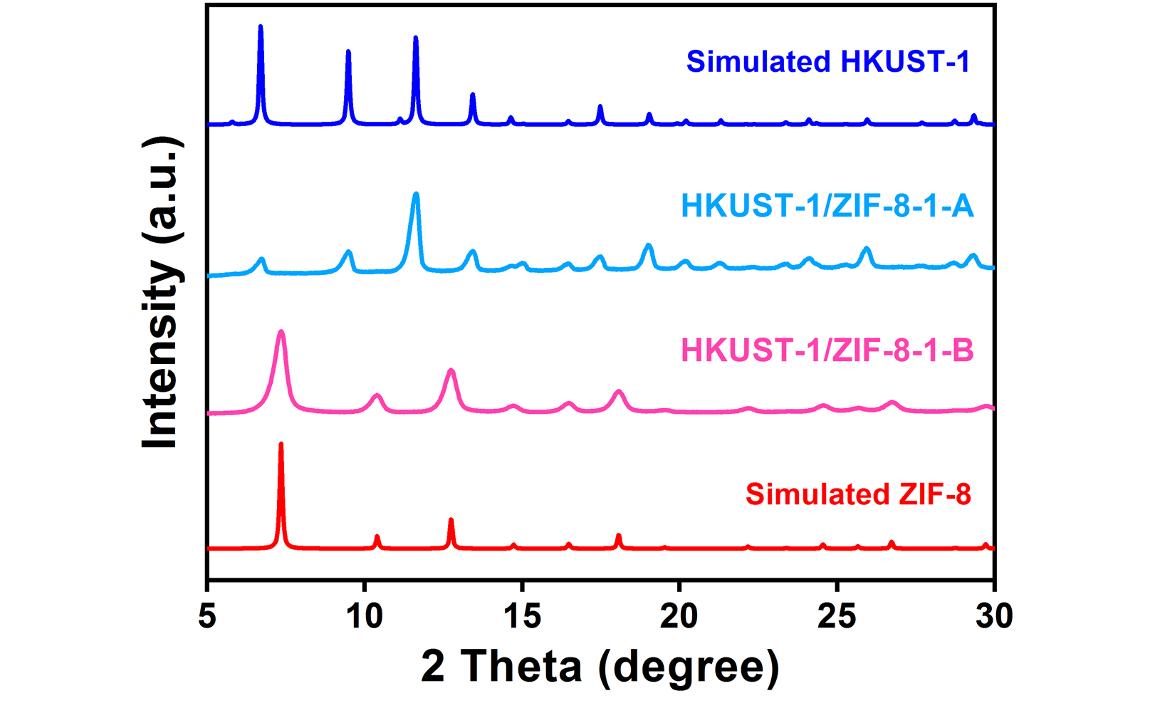
**

**Figure S3.** PXRD patterns of HKUST-1/ZIF-8-1-A and HKUST-1/ZIF-8-1-B, which are the etched samples of HKUST-1/ZIF-8-1 by HAc and NH_3_·H_2_O, respectively. The PXRD peaks of HKUST-1/ZIF-8-1-A and HKUST-1/ZIF-8-1-B match well with those of HKUST-1 and ZIF-8, respectively.

**
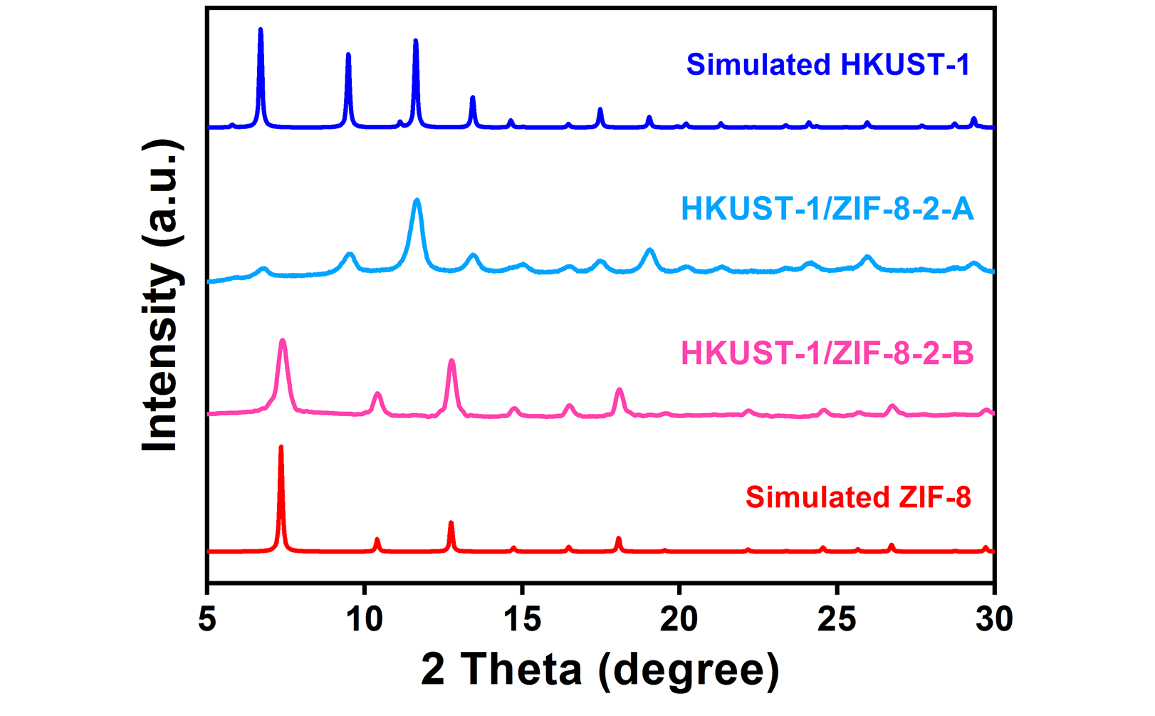
**

**Figure S4.** PXRD patterns of HKUST-1/ZIF-8-2-A and HKUST-1/ZIF-8-2-B, which are the etched samples of HKUST-1/ZIF-8-2 by HAc and NH_3_·H_2_O, respectively. The PXRD peaks of HKUST-1/ZIF-8-2-A and HKUST-1/ZIF-8-2-B match well with those of HKUST-1 and ZIF-8, respectively.


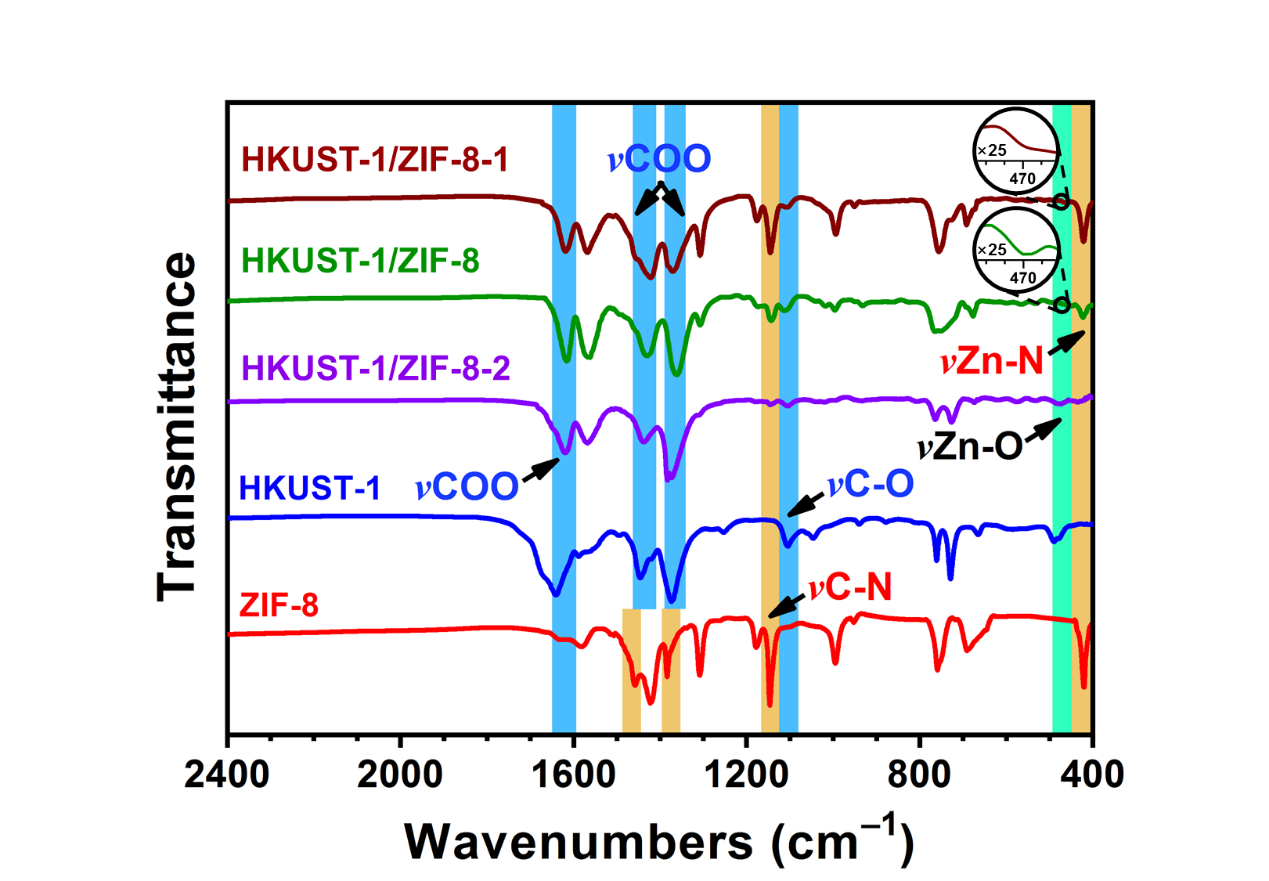


**Figure S5.** Comparison of the FT-IR spectra of HKUST-1/ZIF-8, HKUST-1/ZIF-8-1 and HKUST-1/ZIF-8-2. FT-IR spectra display the main characteristic signals of HKUST-1 and ZIF-8 in the samples of dual-MOFs, and the absorption peak intensity is variable depending on the contents of HKUST-1 and ZIF-8.


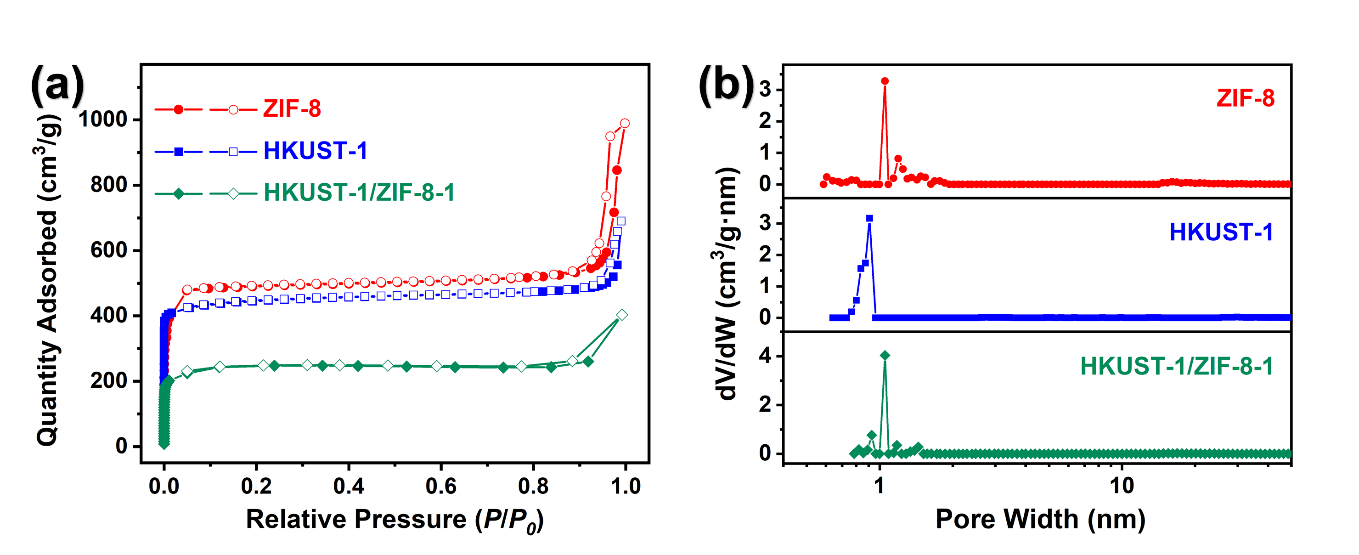


**Figure S6.** (a) N_2_ adsorption/desorption isotherms and (b) pore size distribution of HKUST-1/ZIF-8-1.


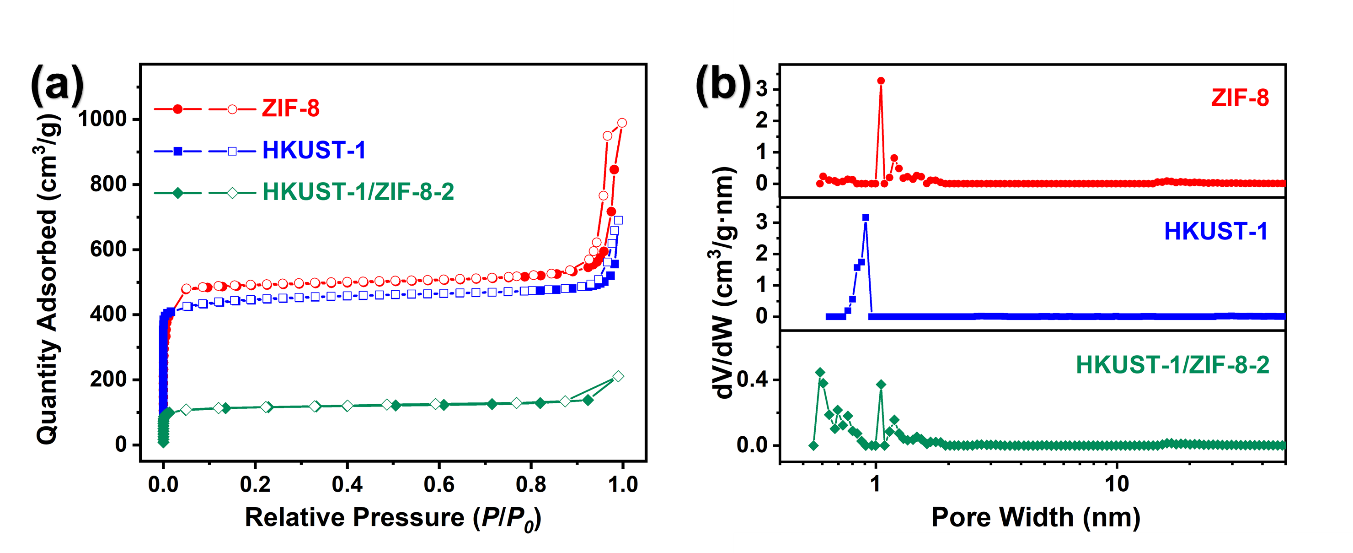


**Figure S7.** (a) N_2_ adsorption/desorption isotherms and (b) pore size distribution of HKUST-1/ZIF-8-2.

**
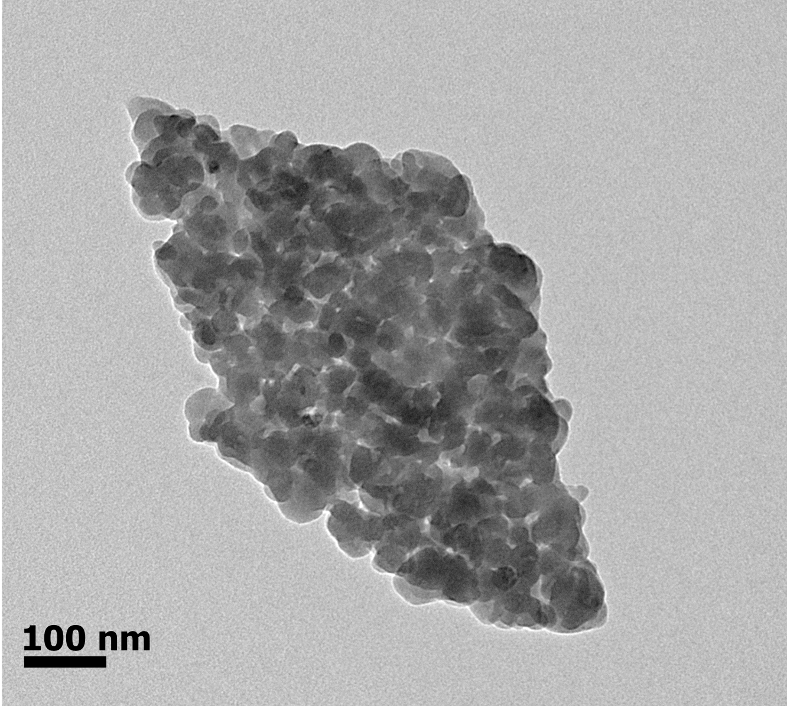
**

**Figure S8.** TEM image of HKUST-1/ZIF-8-1.

**
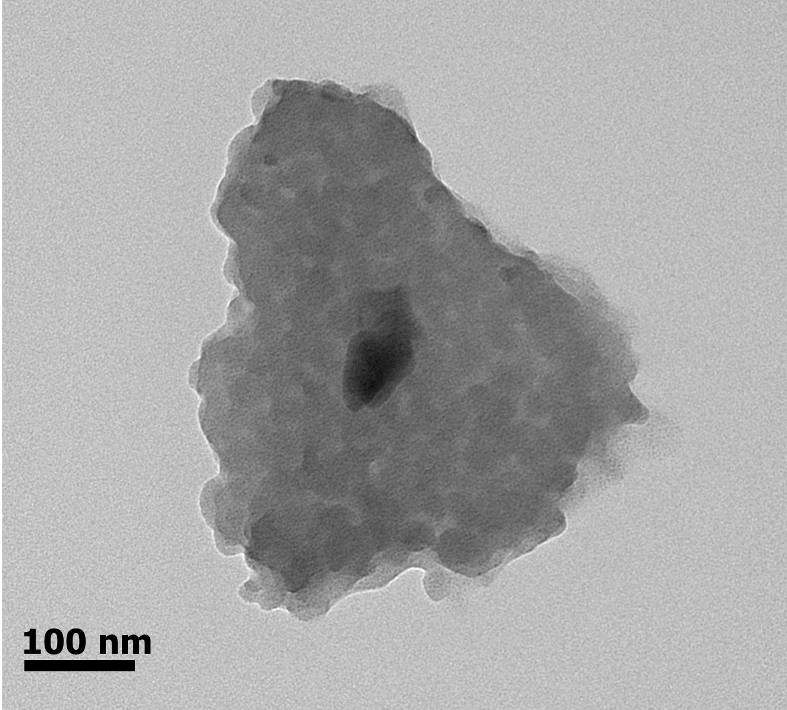
**

**Figure S9.** TEM image of HKUST-1/ZIF-8-2.


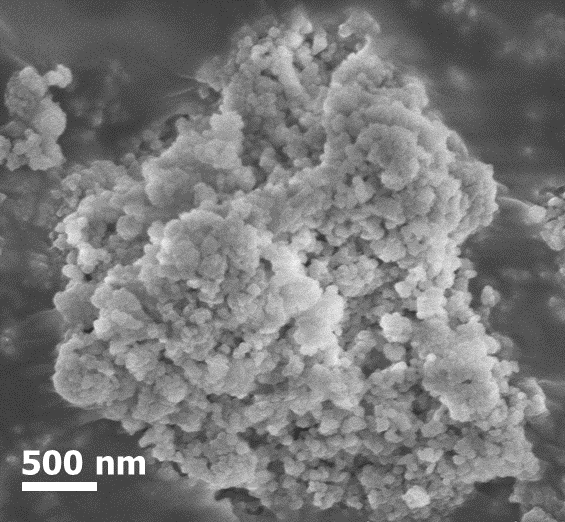


**Figure S10.** SEM image of HKUST-1/ZIF-8.


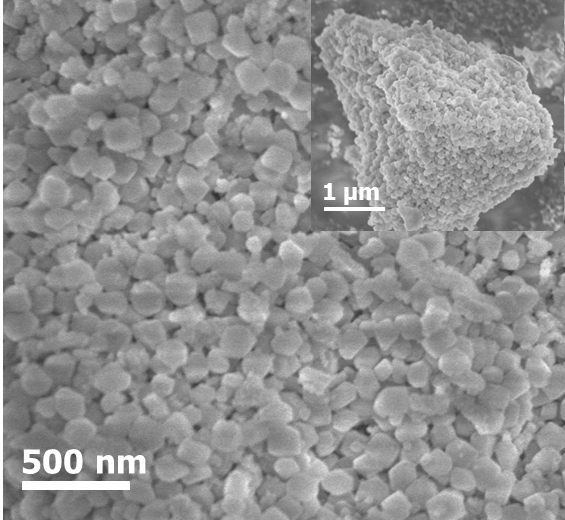


**Figure S11.** SEM image of HKUST-1/ZIF-8-1. The inset shows a low-magnification SEM image.

**
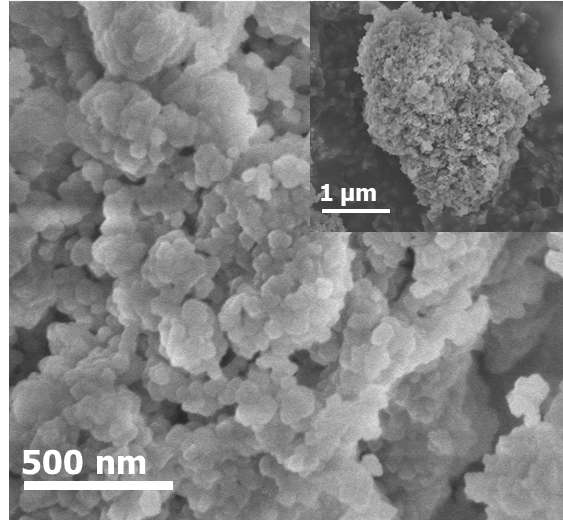
**

**Figure S12.** SEM image of HKUST-1/ZIF-8-2. The inset shows a low-magnification SEM image.


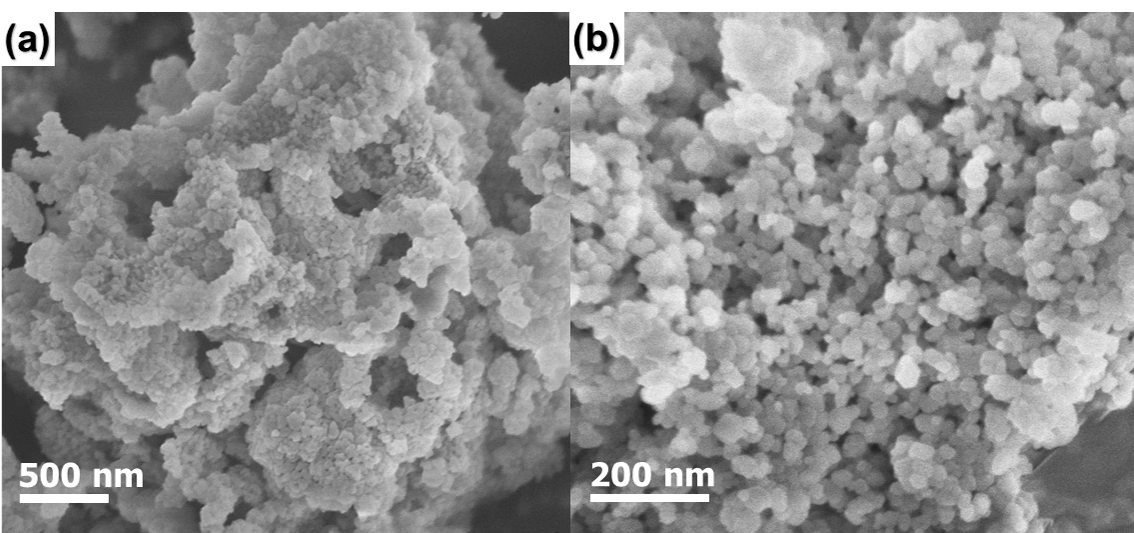


**Figure S13.** SEM images of (a) HKUST-1/ZIF-8-A and (b) HKUST-1/ZIF-8-B.


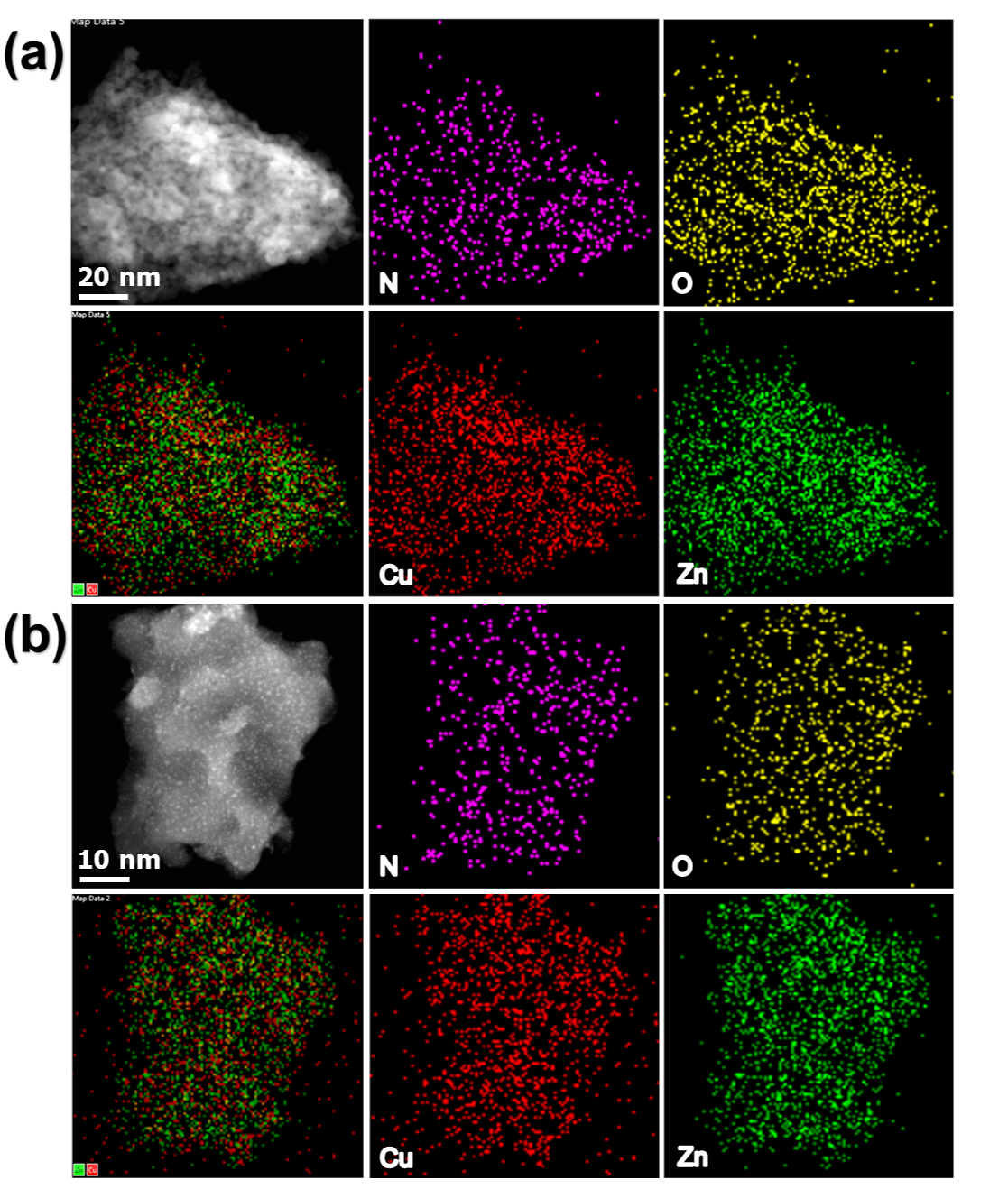


**Figure S14.** HAADF-STEM and EDX element mapping images of HKUST-1/ZIF-8-1.

**
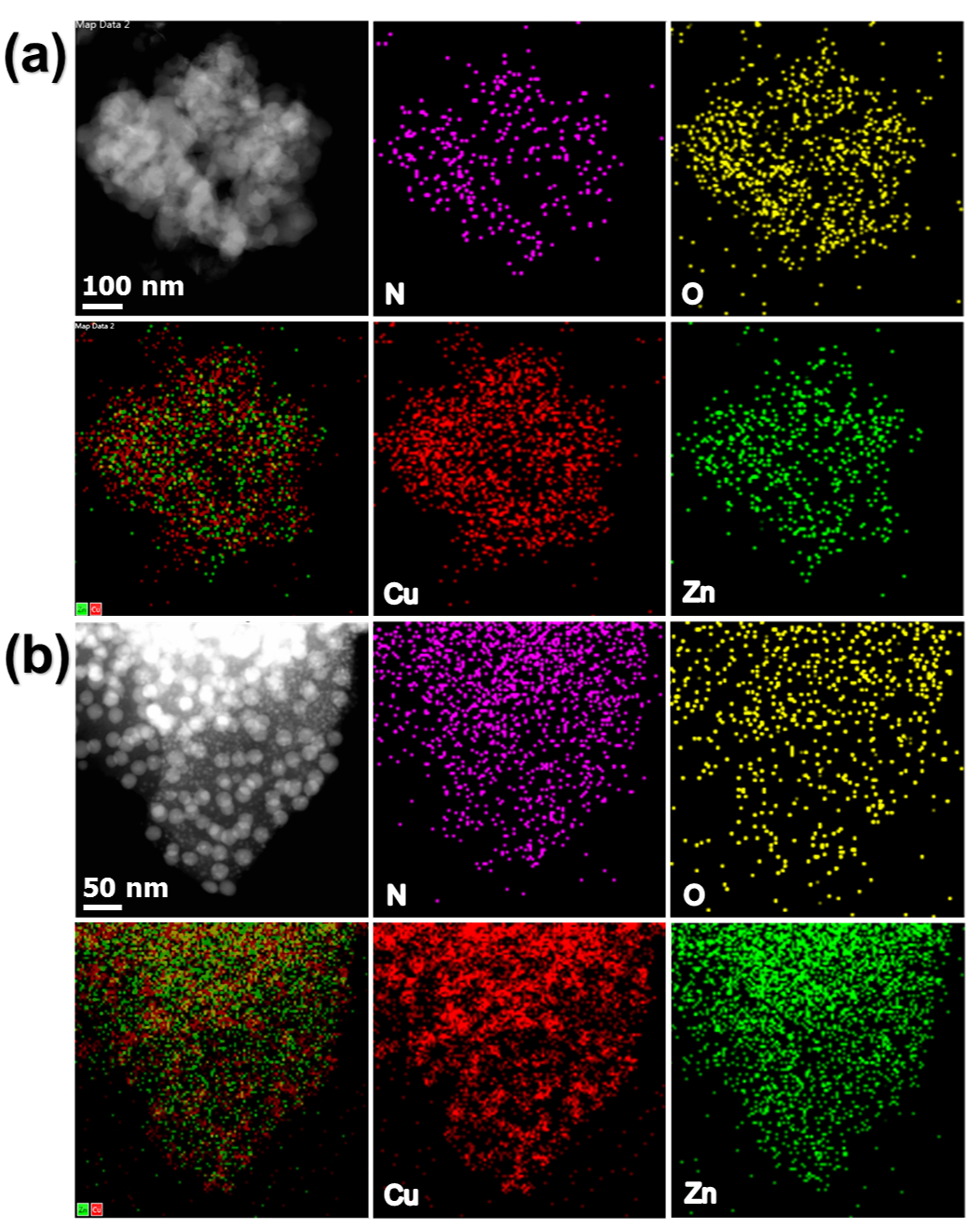
**

**Figure S15.** HAADF-STEM and EDX element mapping images of HKUST-1/ZIF-8-2.

**
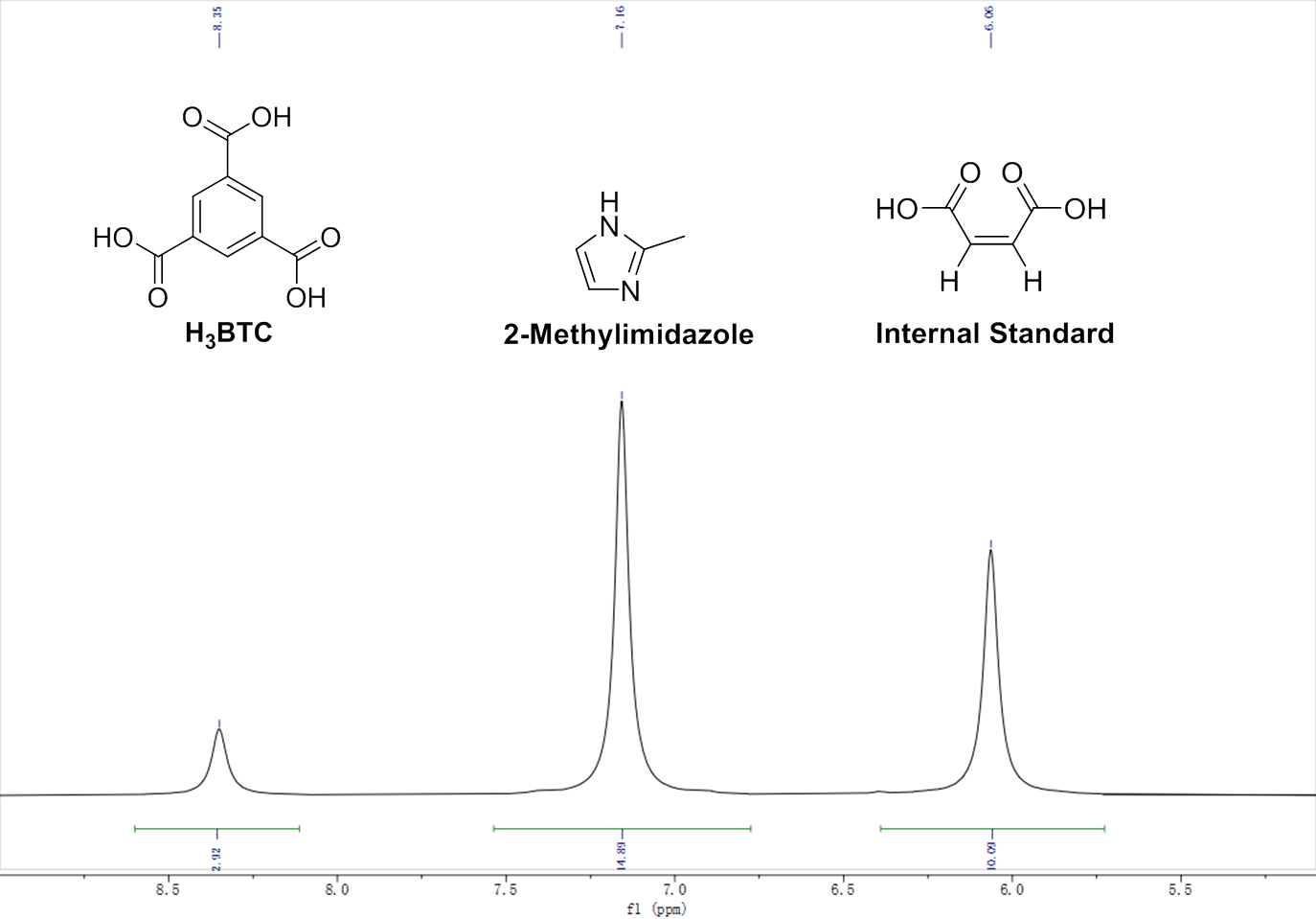
**

**Figure S16.** ^1^H NMR spectrum of digested HKUST-1/ZIF-8 (20.0 mg) in D_2_SO_4_/DMSO-d_6_ solution. The amounts of H_3_BTC and 2-methylimidazole were quantified to be 0.017 and 0.122 mmol, respectively, by using maleic acid as an internal standard. The contents of Cu and Zn are 0.034 and 0.059 mmol, respectively, as determined by ICP-OES, affording about one H_3_BTC ligand per [Cu_2_(COO)_4_] unit and two 2-methylimidazole ligands per Zn center.


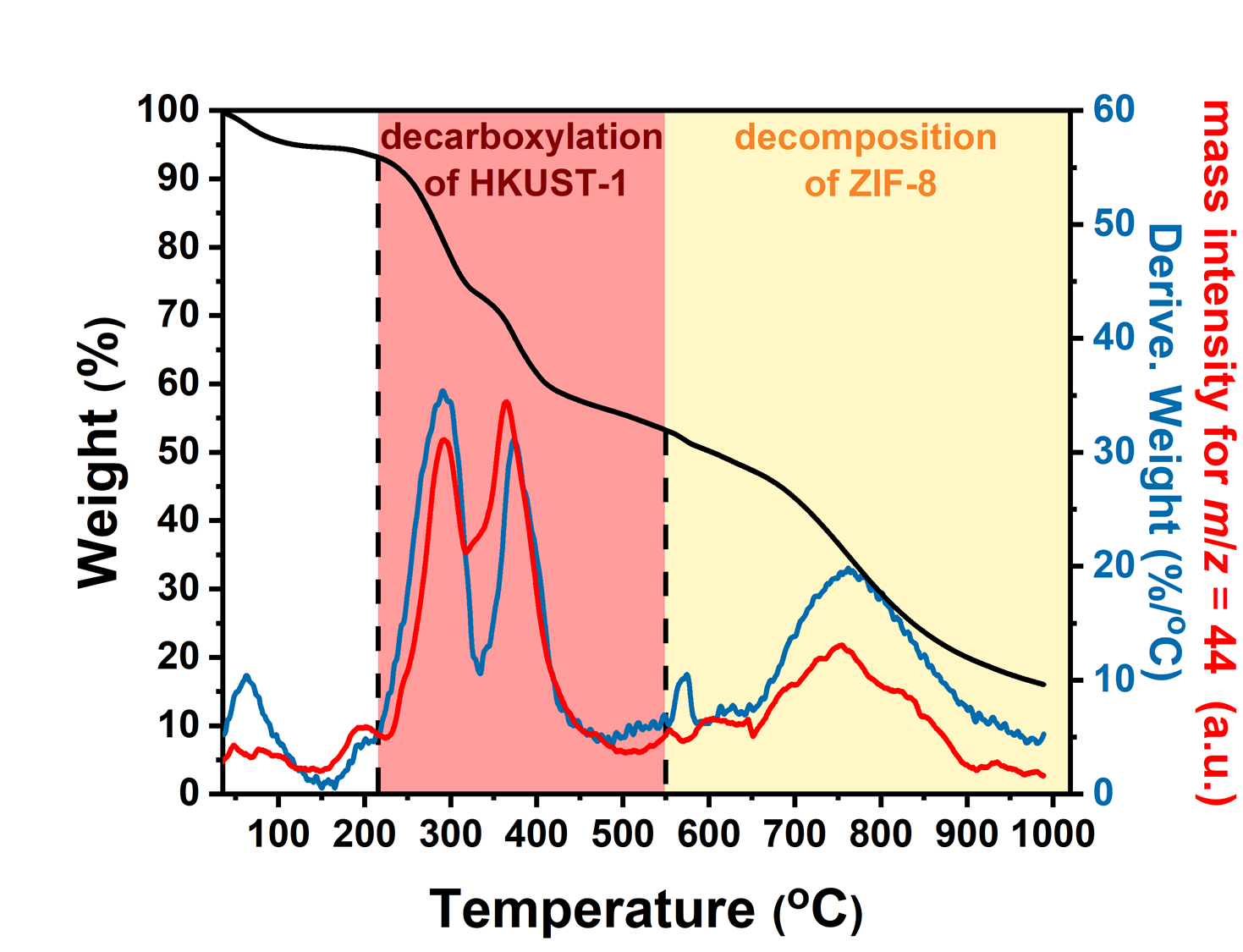


**Figure S17.** TG and DTG curves for HKUST-1/ZIF-8.


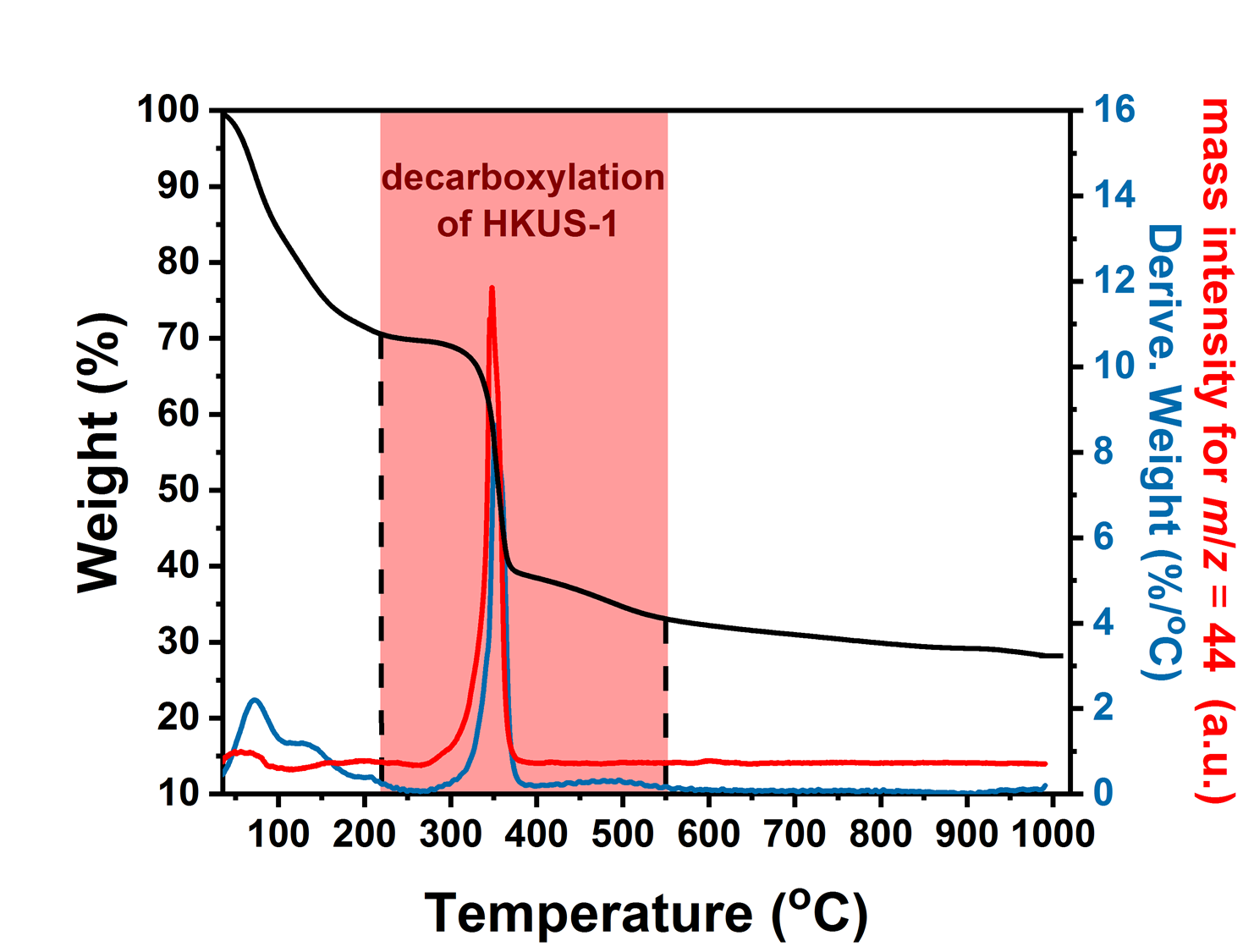


**Figure S18.** TG and DTG curves for HKUST-1.

**
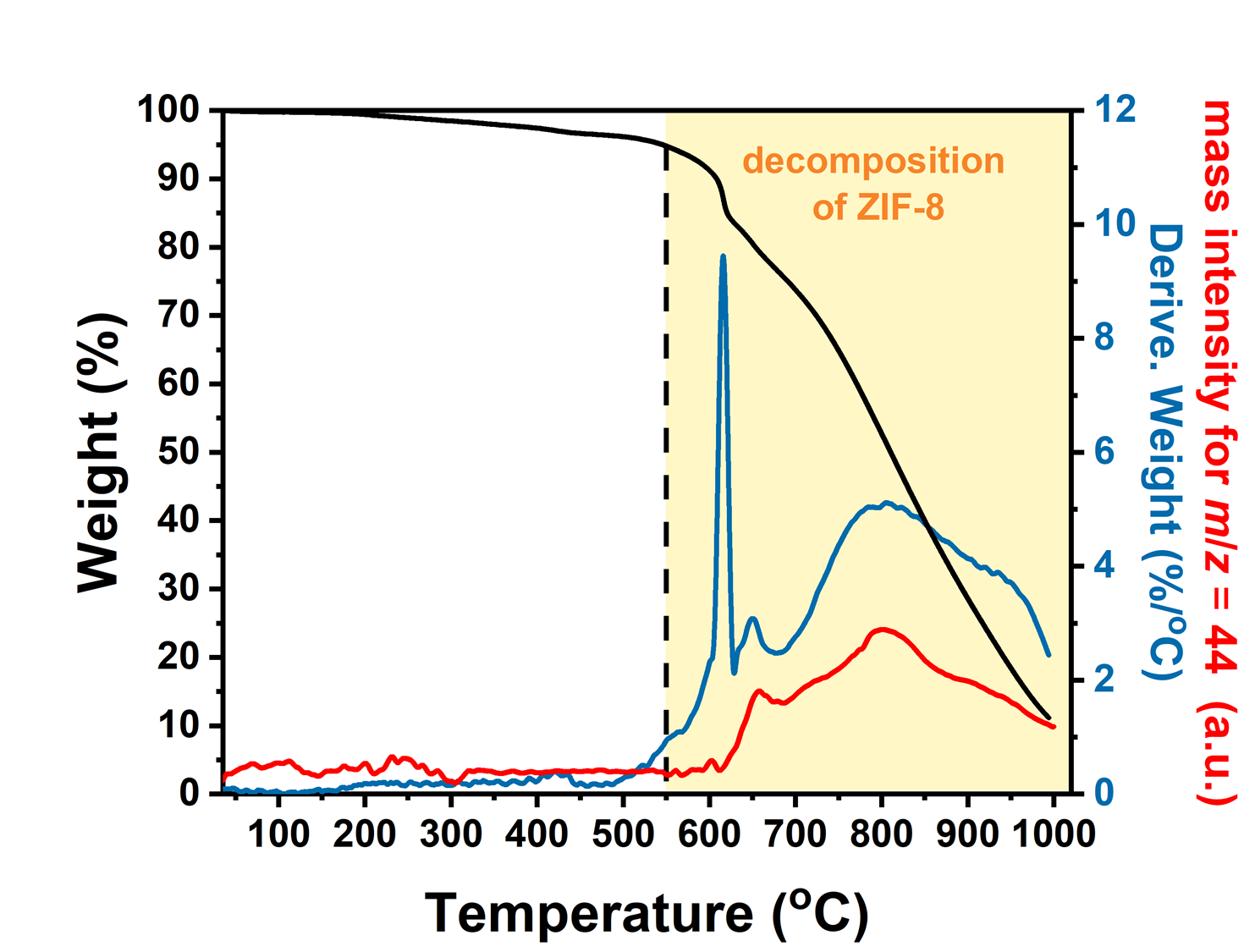
**

**Figure S19.** TG and DTG curves for ZIF-8.


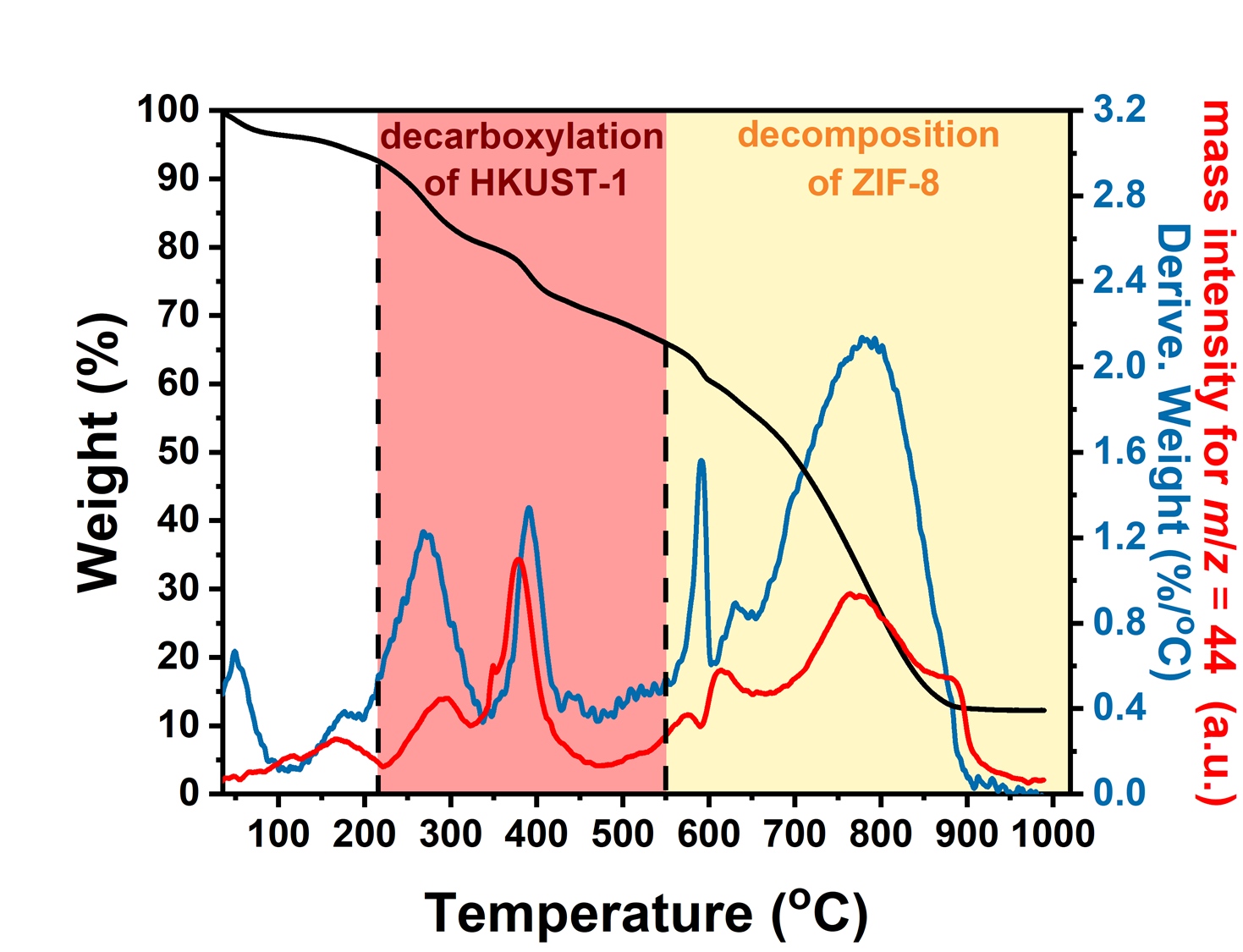


**Figure S20.** TG and DTG curves for HKUST-1/ZIF-8-1.


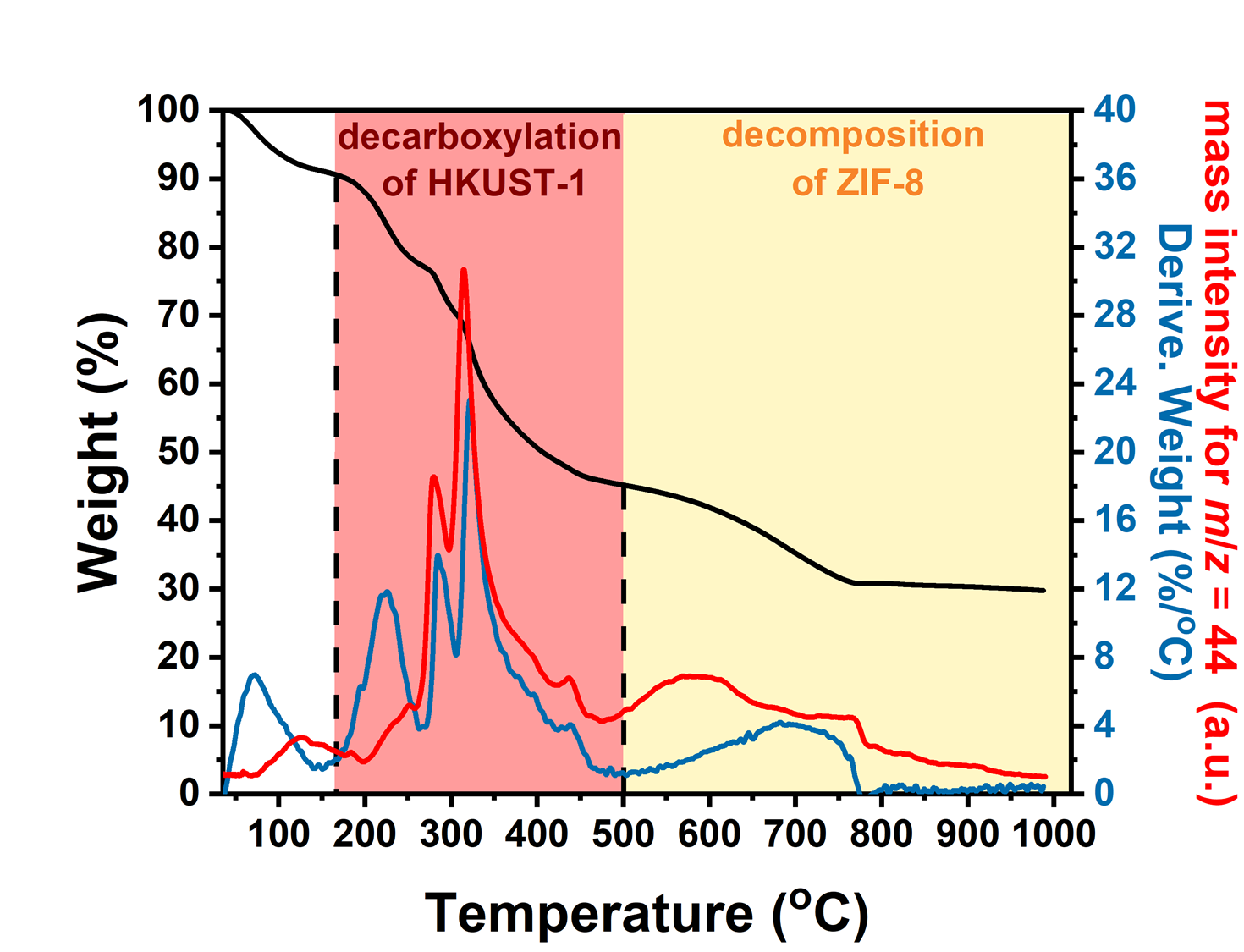


**Figure S21.** TG and DTG curves for HKUST-1/ZIF-8-2.


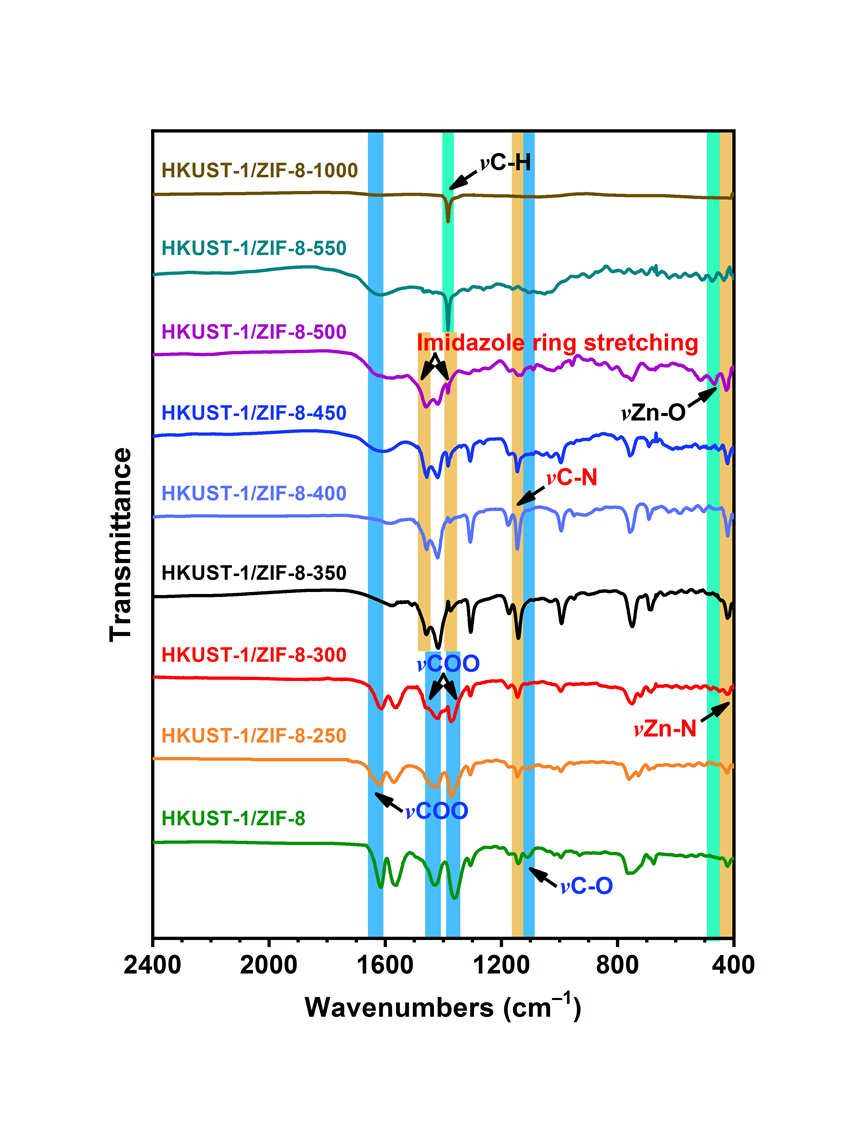


**Figure S22.** FT-IR spectra of HKUST-1/ZIF-8-T, where T represents the annealing temperature.

FT-IR spectra were used to monitor the organic moieties in the annealed samples of HKUST-1/ZIF-8. The characteristic signals of HKUST-1 appeared at 1620, 1439 and 1363 cm^−1^ should be assigned to the COO–Cu_2_ stretching vibration, and the absorption band at 1110 cm^−1^ is observed for the C–O stretching vibration. No obvious change of the absorption peaks in the FT-IR spectrum of HKUST-1/ZIF-8-250 suggests that the main structure of HKUST-1 remains intact. When the annealing temperature was raised to 300 ^o^C, the stretching vibration intensities for the carboxylate groups are significantly weakened, indicating that decarboxylation reaction occurred. Upon raising the annealing temperature to 350 ^o^C, the vibration bands for the carboxylate groups almost disappeared, which should be originated from decarboxylation of the carboxylate moieties in HKUST-1. In the meantime, the characteristic entire ring stretching vibrations of imidazole ligands at 1460 and 1380 cm^−1^ became predominant. The characteristic absorption peaks of ZIF-8, C–N stretching vibration at 1148 cm^−1^ and Zn–N stretching vibration at 421 cm^−1^, remain intact when the annealing temperature was raised to 500 ^o^C, indicating that the ZIF-8 skeleton retained. These characteristic peaks for ZIF-8 disappeared upon raising the temperature to 550 ^o^C, due to decomposition of ZIF-8. At this stage (500–550 ^o^C), there appeared the Zn–O stretching vibration at 470 cm^−1^, indicating the formation of ZnO, which confirmed the occurrence of decomposition of the ZIF-8 skeleton. Further raising the annealing temperature to 1000 ^o^C, the organic residues suffered from severe carbonization.


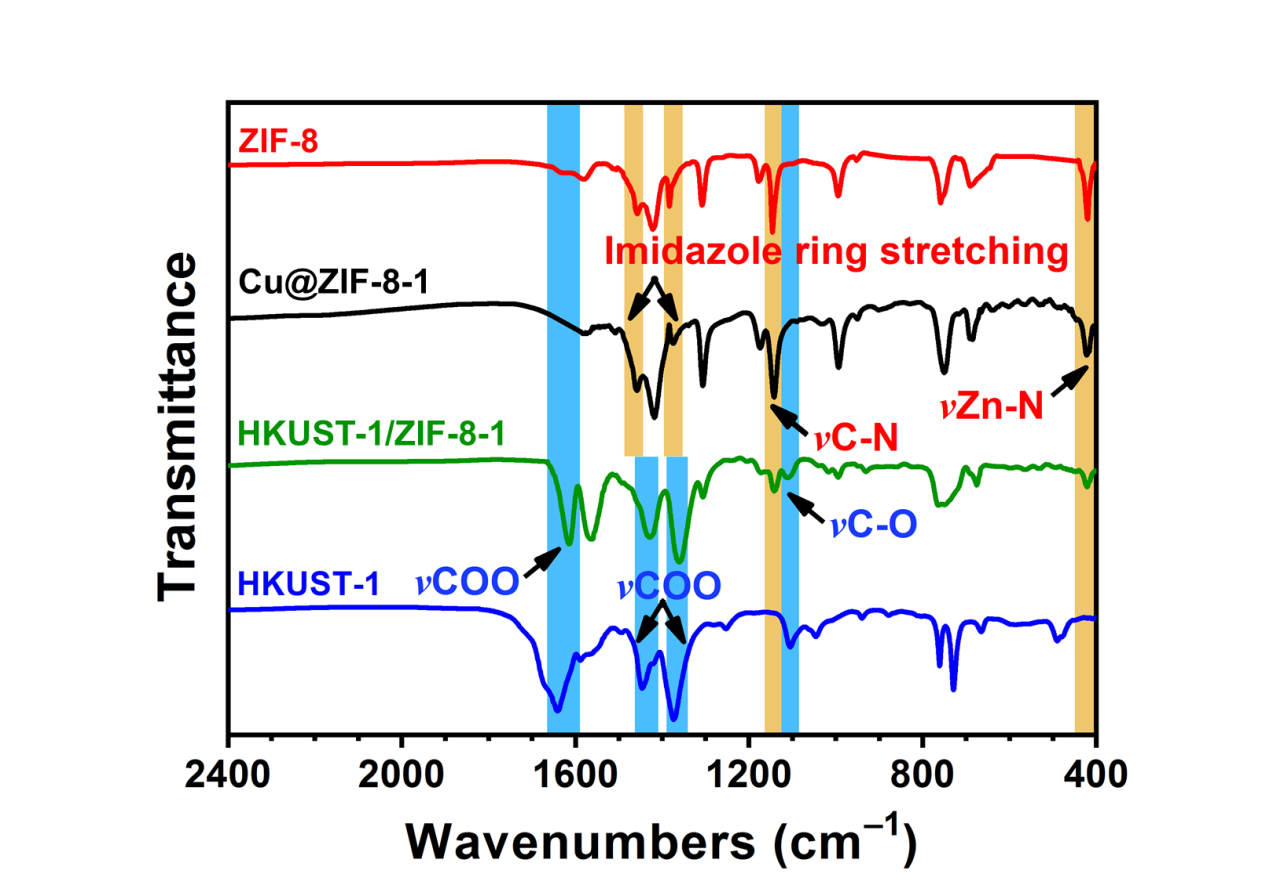


**Figure S23.** Comparison of the FT-IR spectra of HKUST-1/ZIF-8-1 and Cu@ZIF-8-1. Cu@ZIF-8-1 is the annealed product of HKUST-1/ZIF-8-1 at 350 ^o^C.


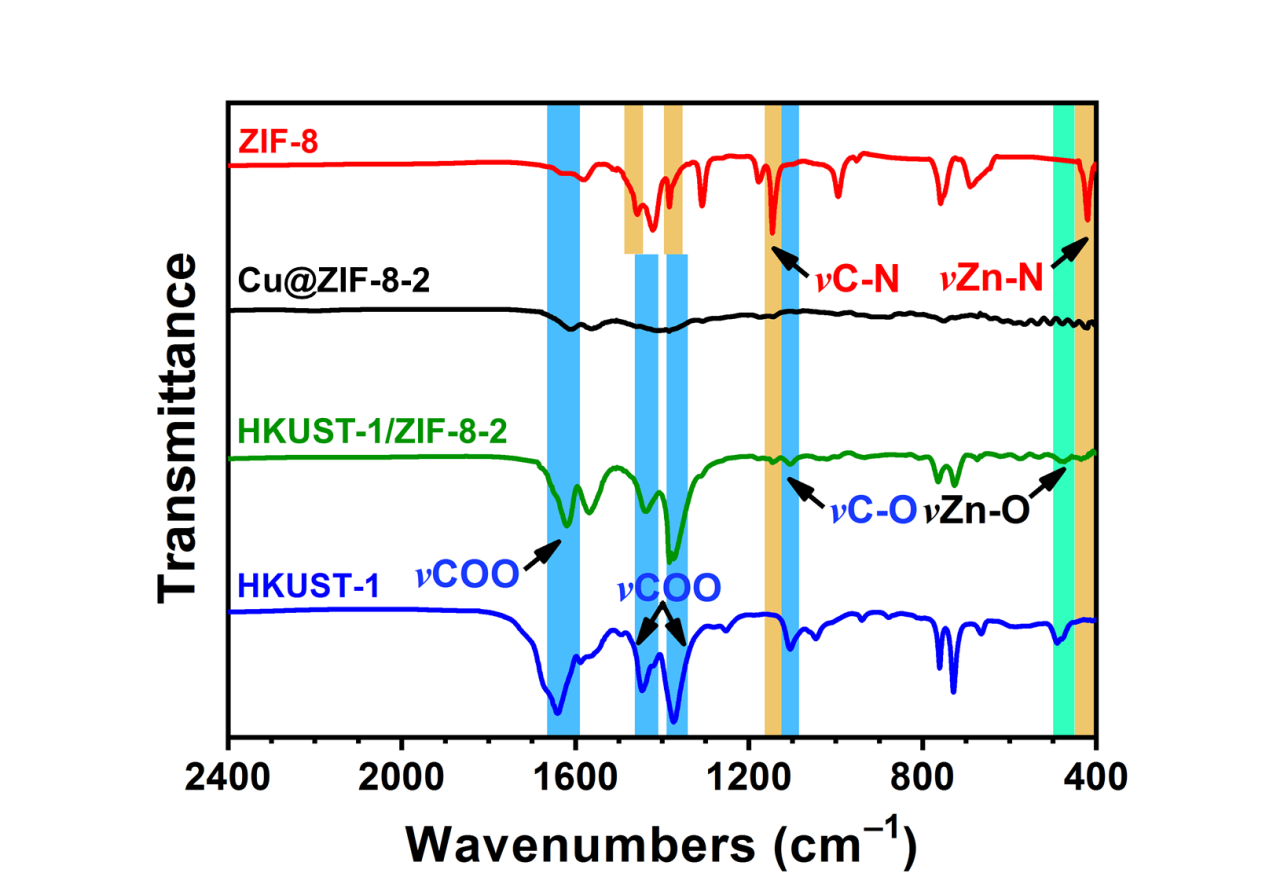


**Figure S24.** Comparison of the FT-IR spectra of HKUST-1/ZIF-8-2 and Cu@ZIF-8-2. Cu@ZIF-8-2 is the annealed product of HKUST-1/ZIF-8-2 at 350 ^o^C.


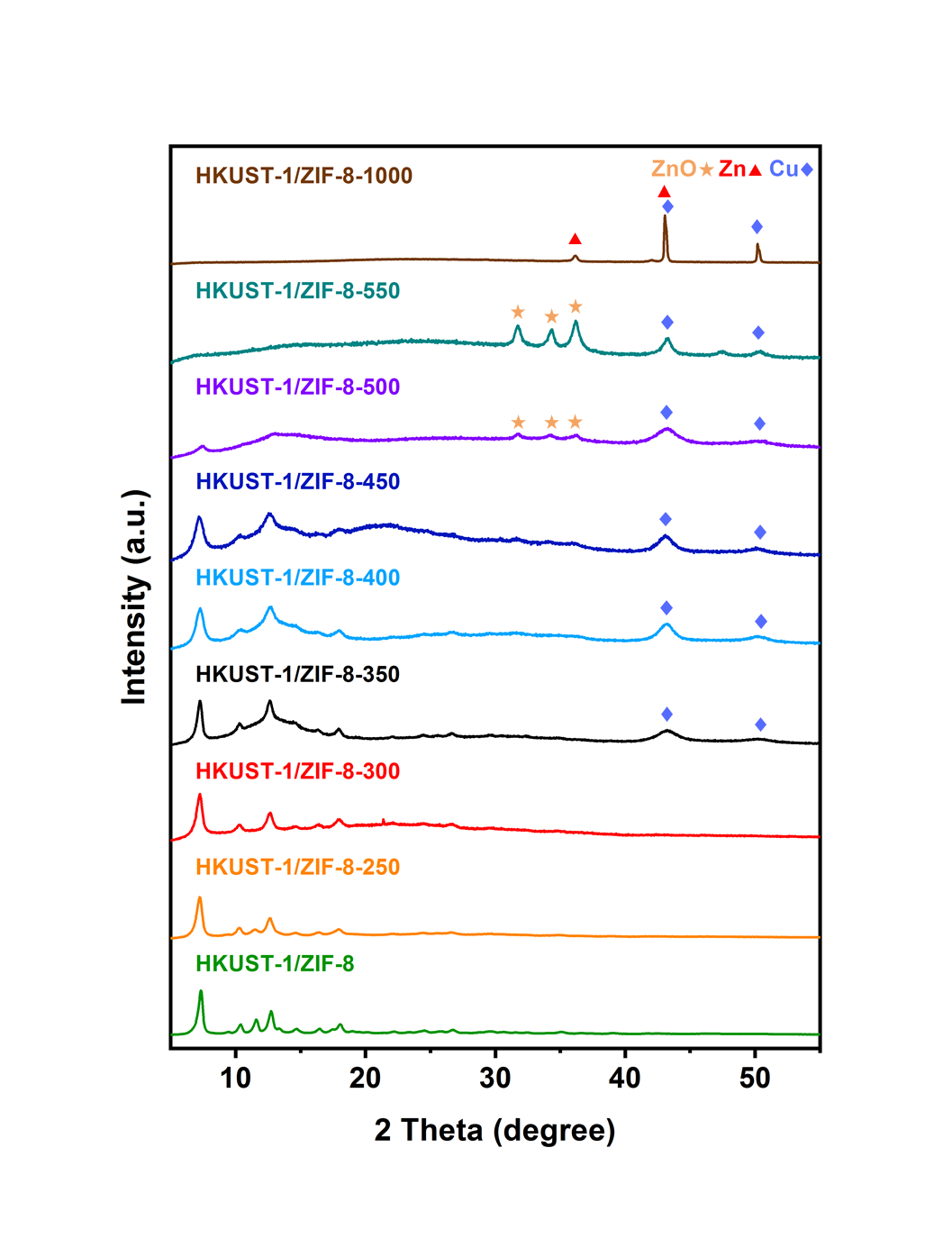


**Figure S25.** PXRD patterns of HKUST-1/ZIF-8-T, where T represents the annealing temperature.

The nature of annealed samples of HKUST-1/ZIF-8 was monitored by power X-ray diffraction (PXRD) studies. As shown in Figure S25, decomposition of the HKUST-1 skeleton was initiated at 300 ^o^C, as the diffraction peaks ((220) and (222)) of HKUST-1 disappeared. When the annealing temperature was raised to 350 ^o^C, there appeared broad characteristic peaks ascribed to metallic Cu (JCPDS, Card No. 04-0836, 43.3 and 50.4^o^), while the diffraction peaks for ZIF-8 retained, indicating successful decomposition of the HKUST-1 skeleton and retaining of the phase of ZIF-8. When the temperature was raised to 500 ^o^C, the diffraction peaks of ZIF-8 disappeared, and there appeared the characteristic diffraction peaks of ZnO (JCPDS, Card No. 21-1486, 31.8, 34.4 and 36.3^o^), indicating decomposition of ZIF-8. Further raising the temperature to 550 ^o^C, the characteristic diffraction peak intensity of ZnO increased. At a higher annealing temperature of 1000 ^o^C, distinct phase transformation was observed, as the ZnO species was gradually reduced to form metallic Zn (JCPDS, Card No. 04-0831, 36.3 and 43.2^o^).


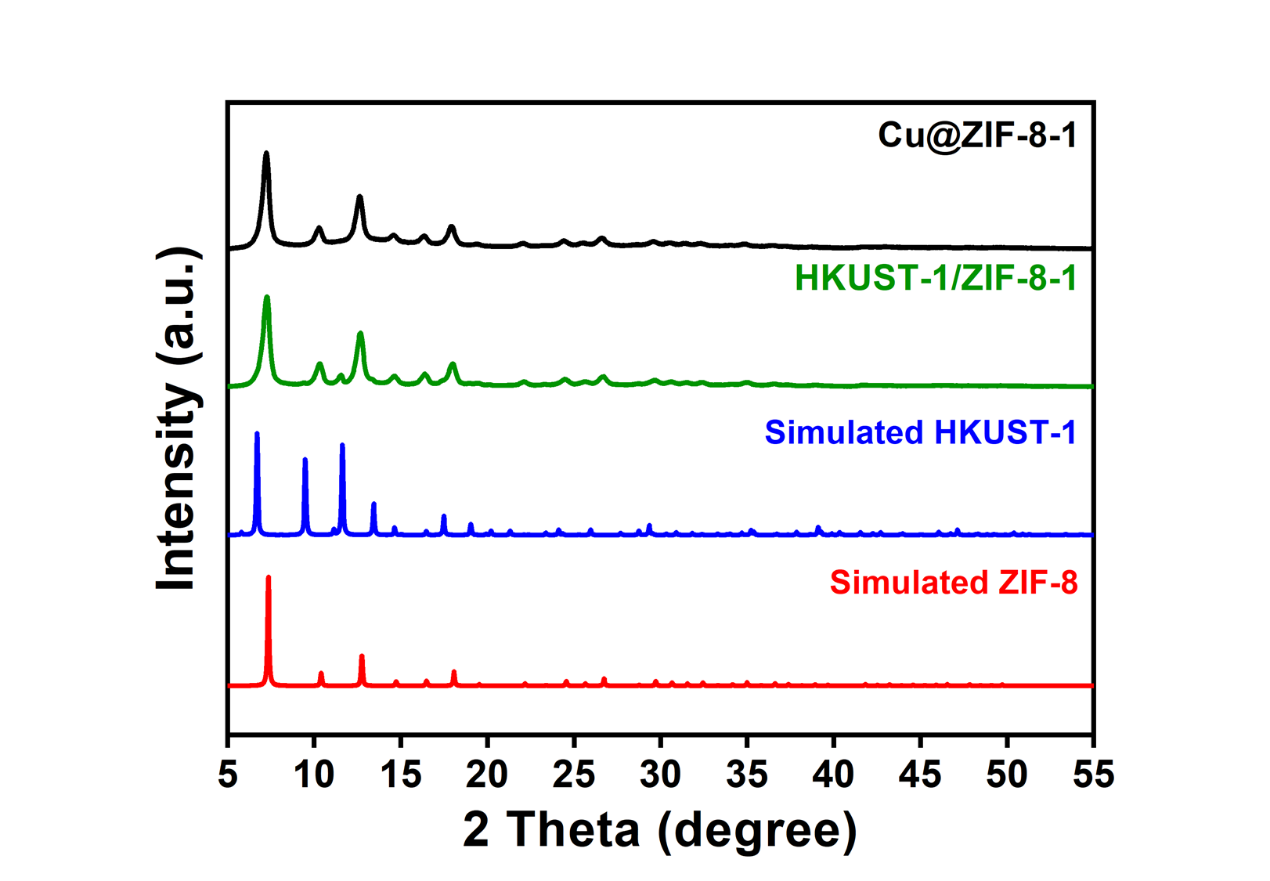


**Figure S26.** Comparison of the PXRD patterns of HKUST-1/ZIF-8-1 and Cu@ZIF-8-1.

**
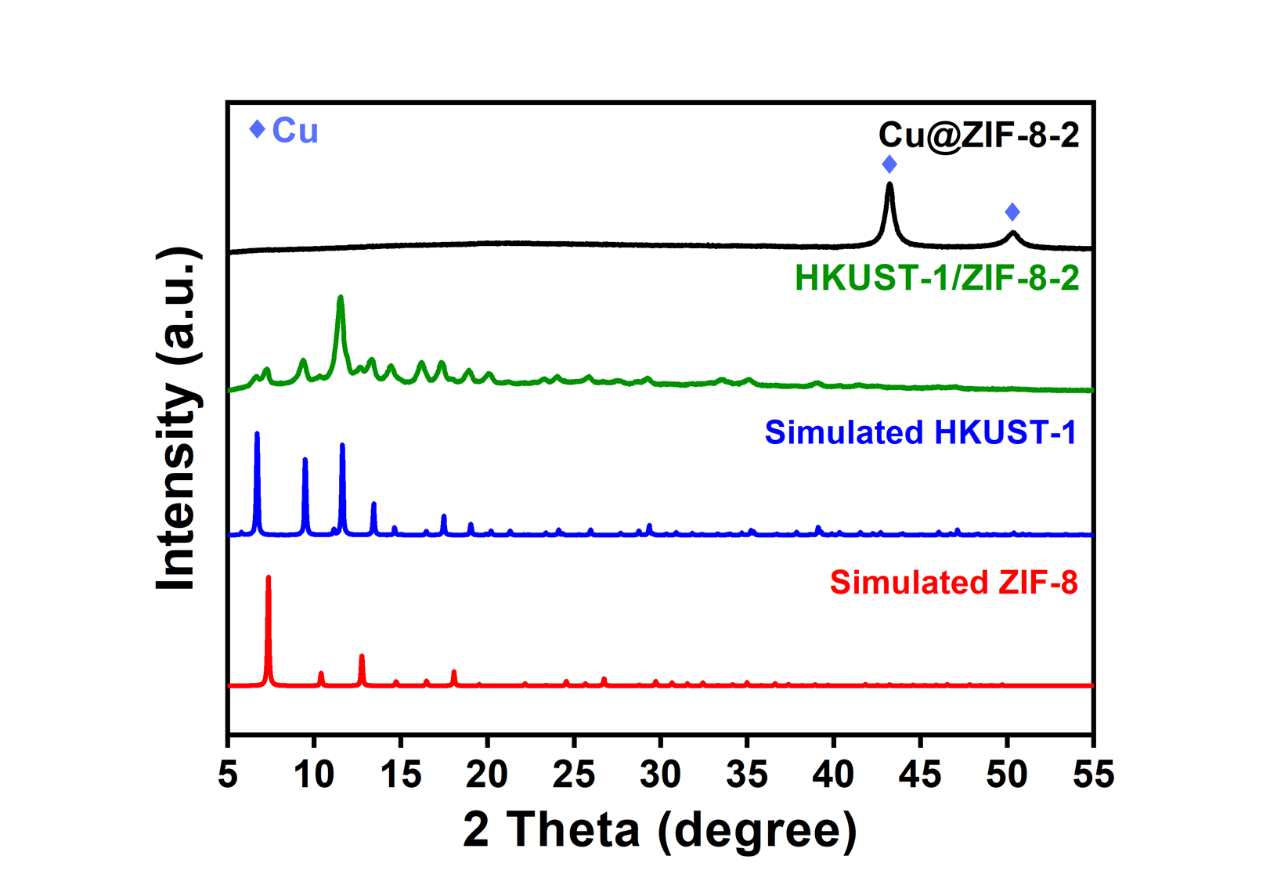
**

**Figure S27.** Comparison of the PXRD patterns of HKUST-1/ZIF-8-2 and Cu@ZIF-8-2.

**
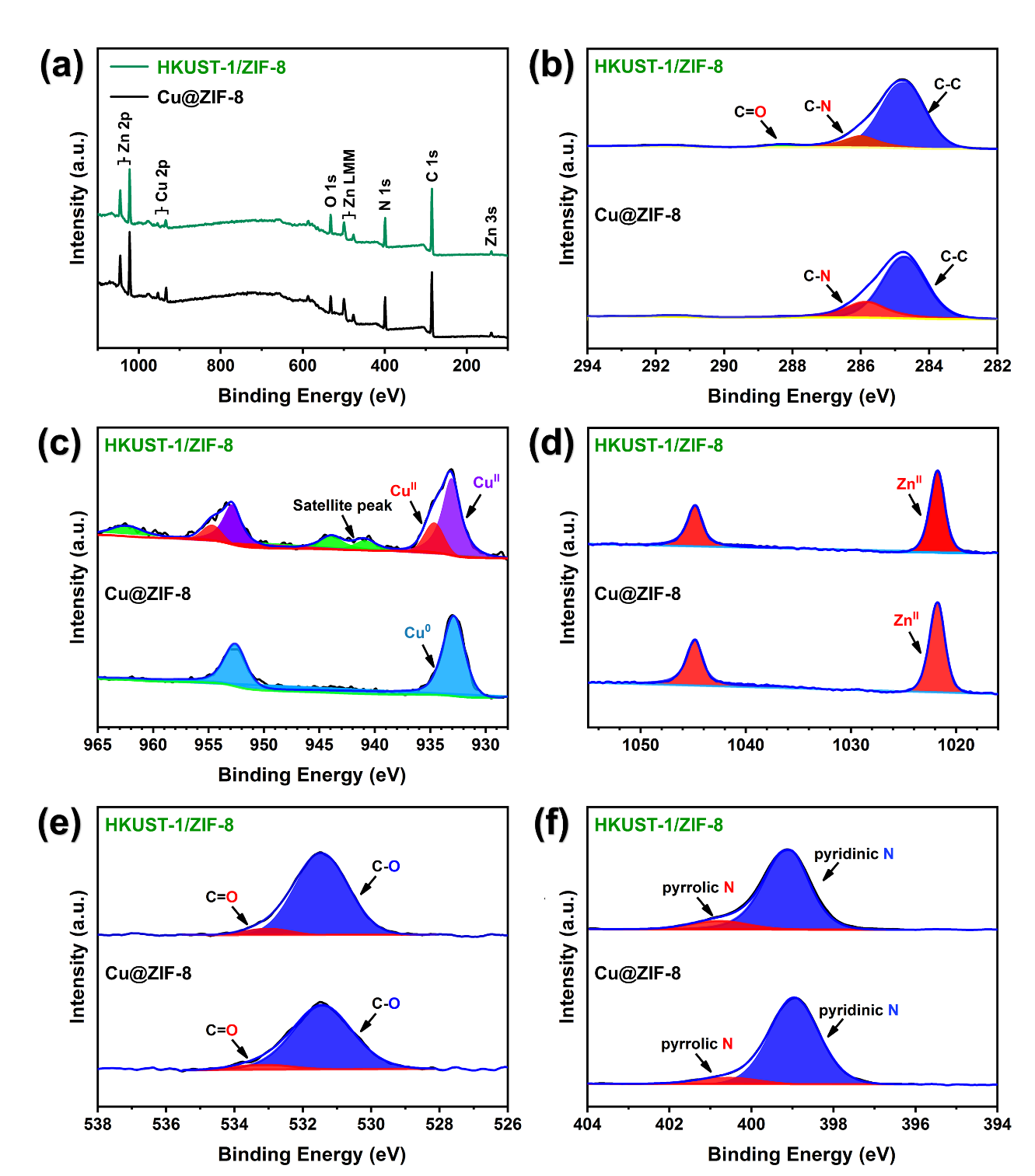
**

**Figure S28.** (a) Survey XPS spectra. (b) C 1s, (c) Cu 2p, (d) Zn 2p, (e) O 1s and (f) N 1s high resolution XPS spectra for HKUST-1/ZIF-8 and Cu@ZIF-8.


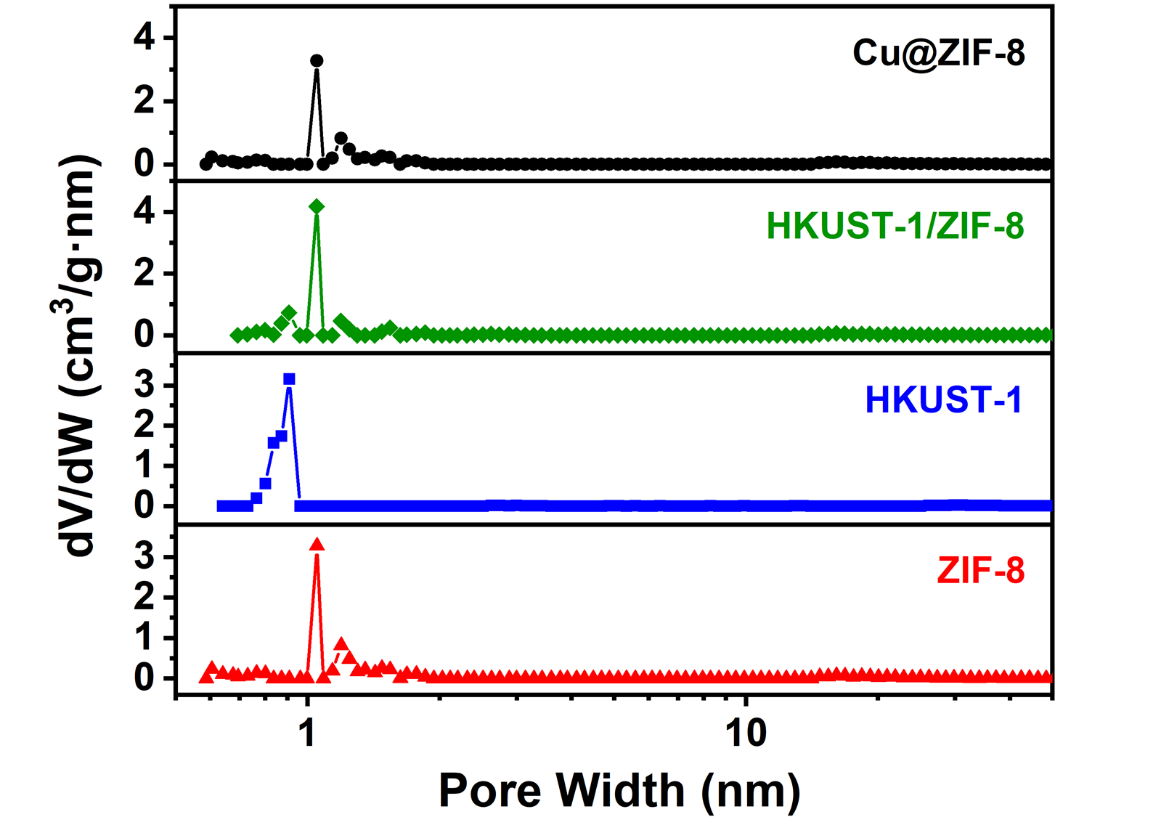


**Figure S29.** Comparison of the pore size distributions for HKUST-1/ZIF-8 and Cu@ZIF-8.


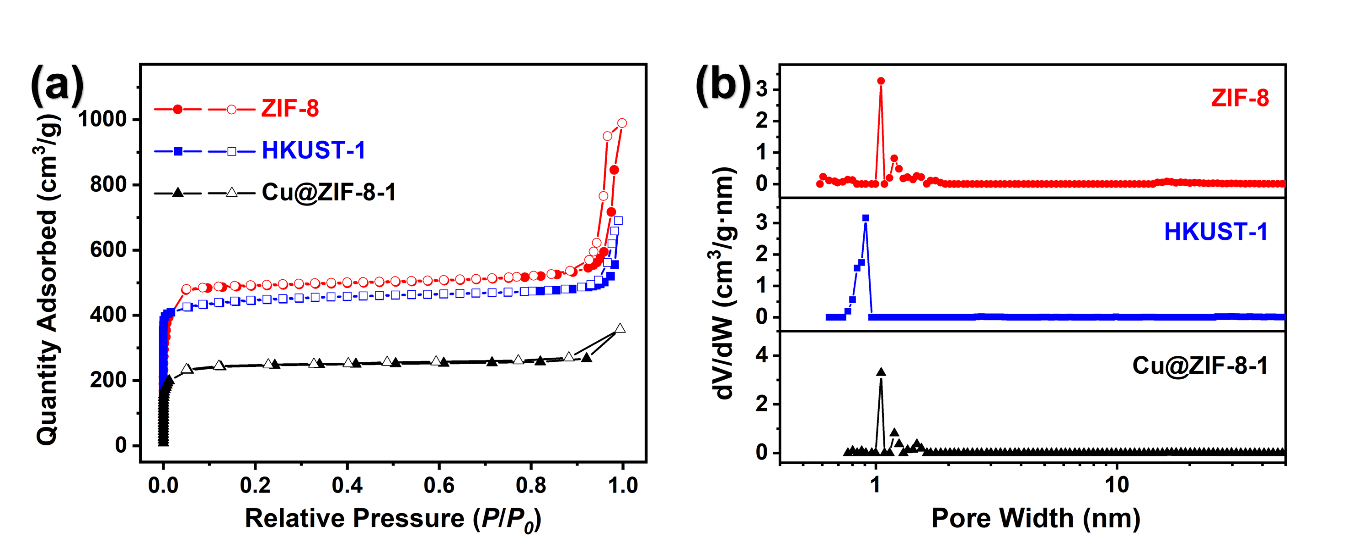


**Figure S30.** (a) N_2_ adsorption/desorption isotherms and (b) pore size distribution of Cu@ZIF-8-1.


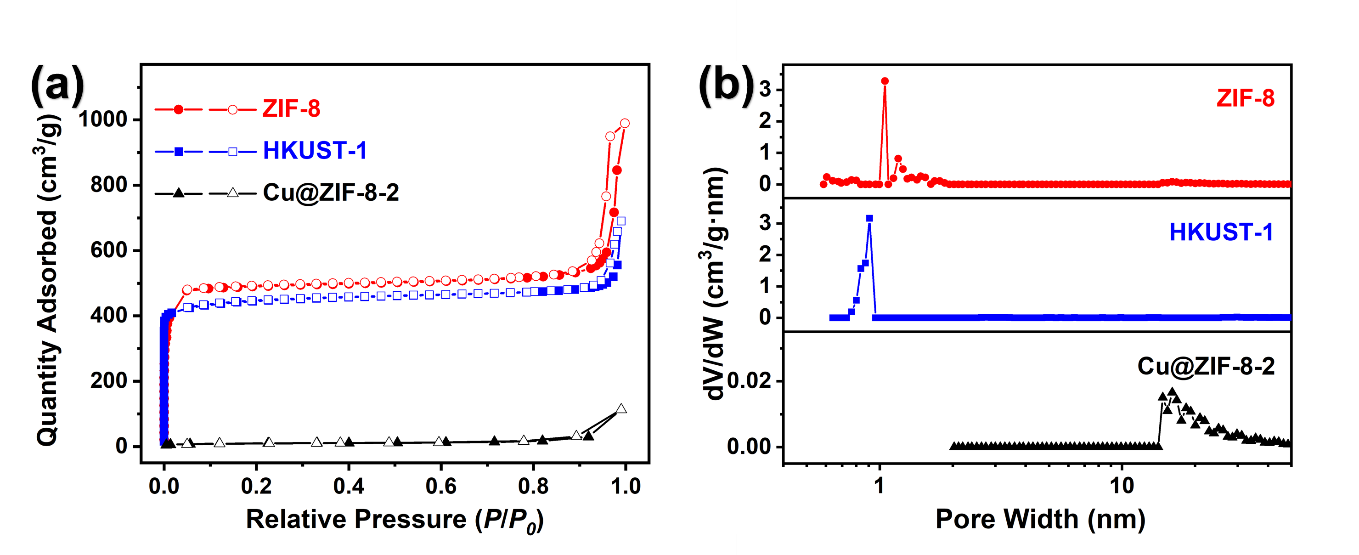


**Figure S31.** (a) N_2_ adsorption/desorption isotherms and (b) pore size distribution of Cu@ZIF-8-2.


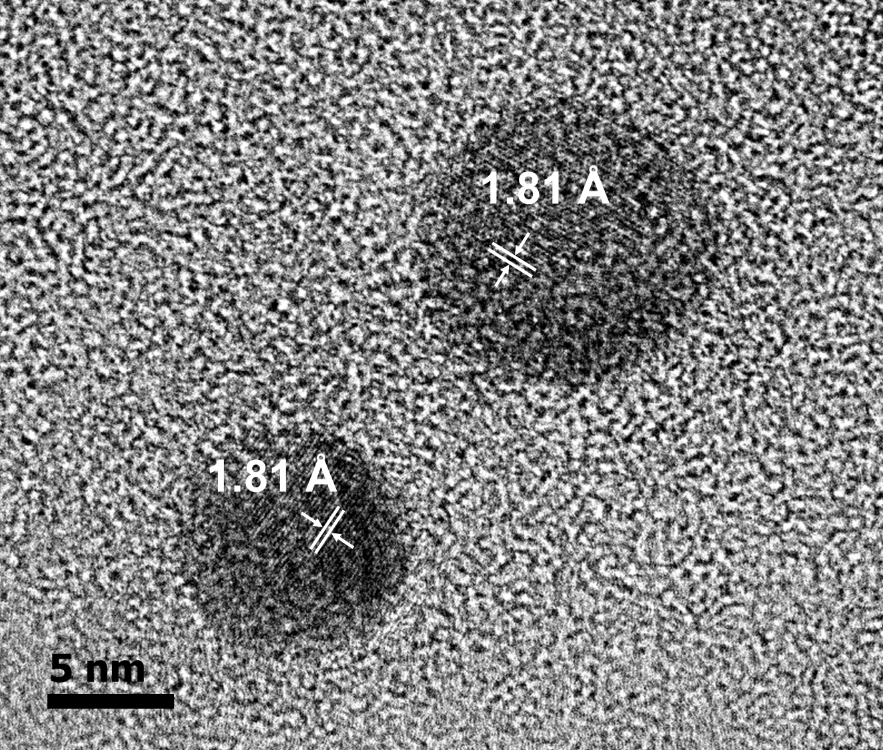


**Figure S32.** HRTEM images of Cu@ZIF-8. The lattice-fringe spacing of the NPs is of 0.181 nm, ascribed to the (200) facet of metallic Cu.


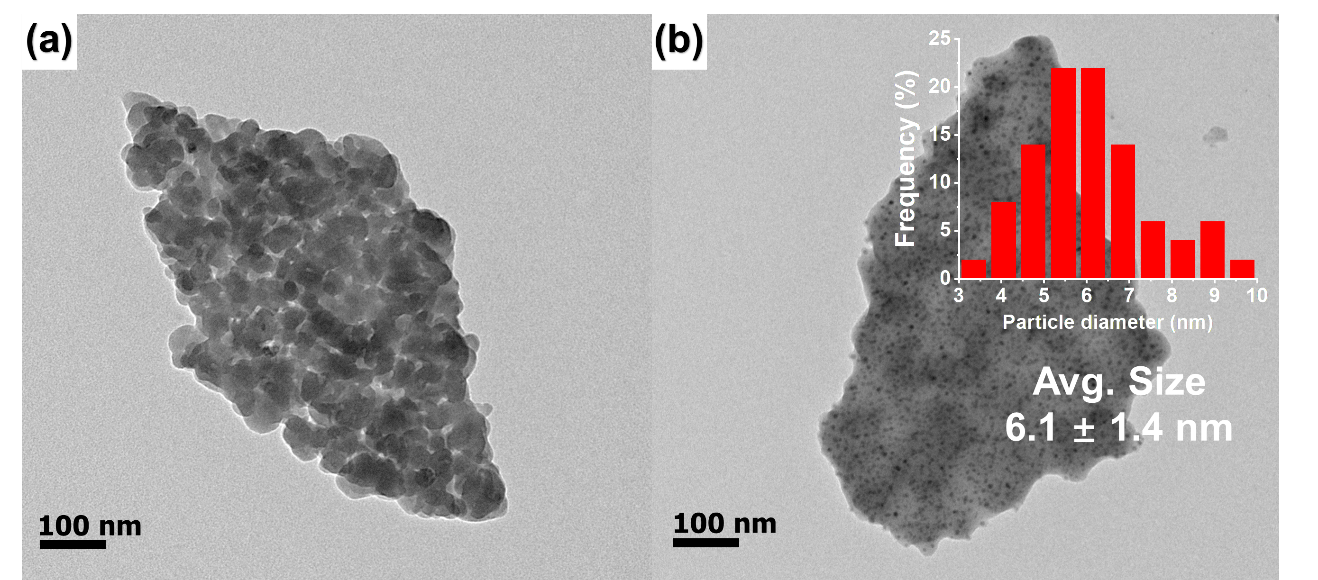


**Figure S33.** TEM images of (a) HKUST-1/ZIF-8-1 and (b) Cu@ZIF-8-1. The inset shows a histogram of metal particle size distribution.

**
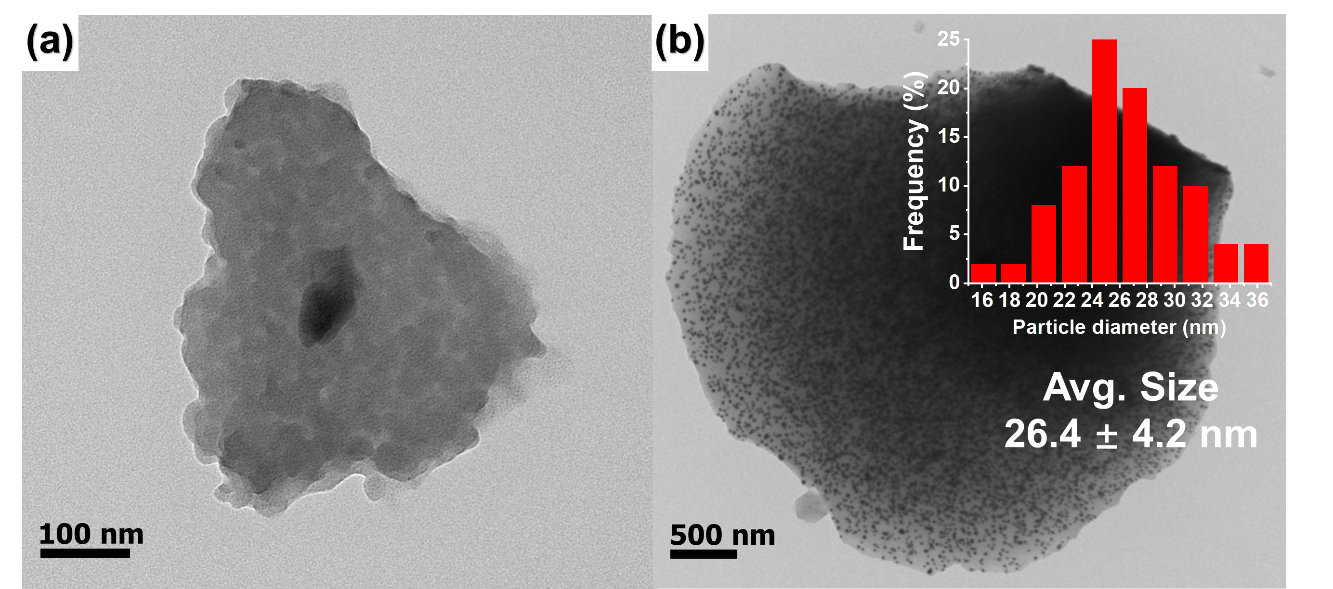
**

**Figure S34.** TEM images of (a) HKUST-1/ZIF-8-2 and (b) Cu@ZIF-8-2. The inset shows a histogram of metal particle size distribution.


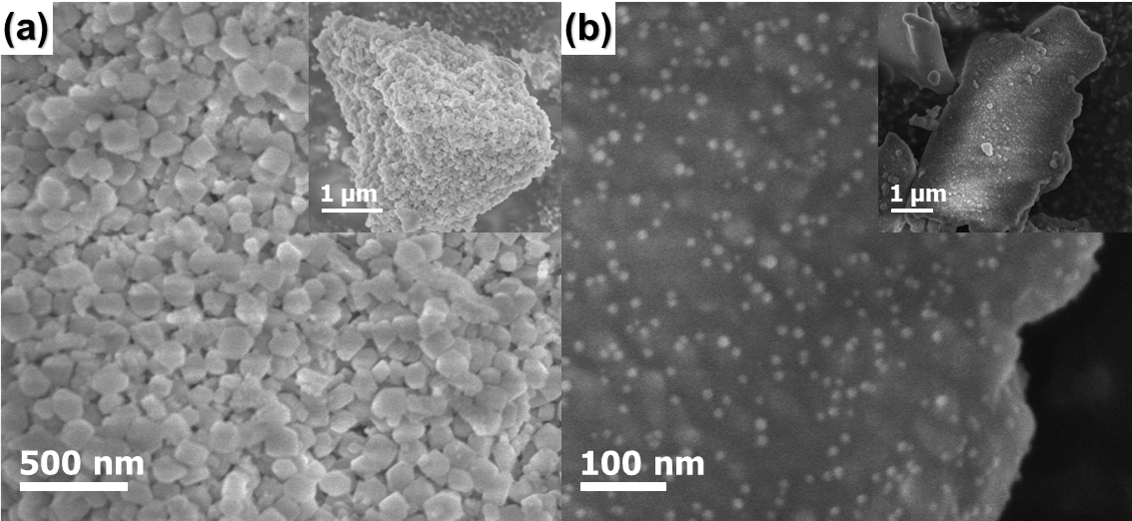


**Figure S35.** SEM images of (a) HKUST-1/ZIF-8-1 and (b) Cu@ZIF-8-1. The insets show the low-magnification SEM images.

**
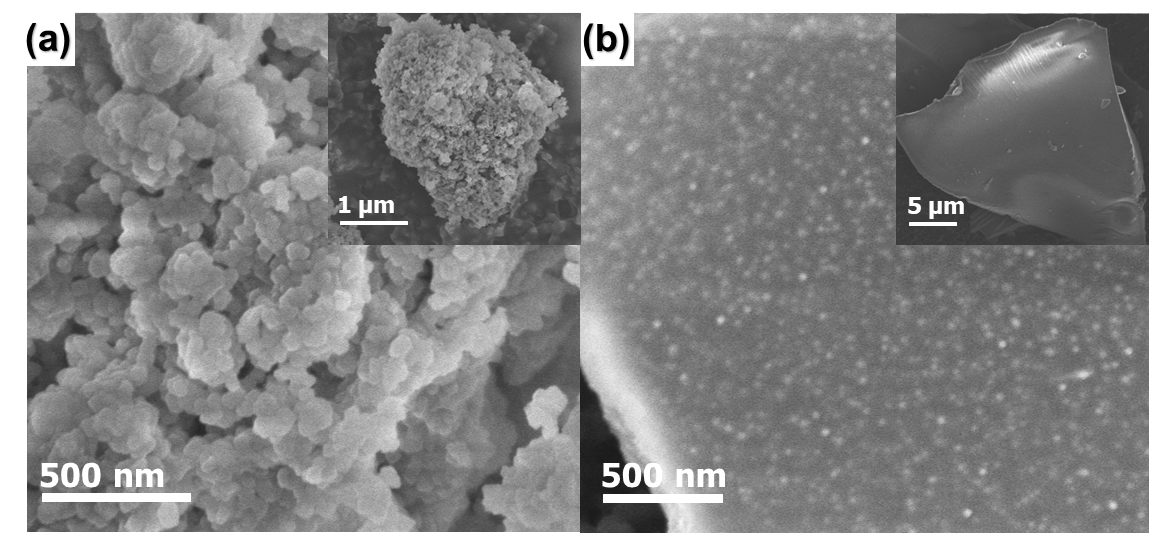
**

**Figure S36.** SEM images of (a) HKUST-1/ZIF-8-2 and (b) Cu@ZIF-8-2. The insets show the low-magnification SEM images.

**
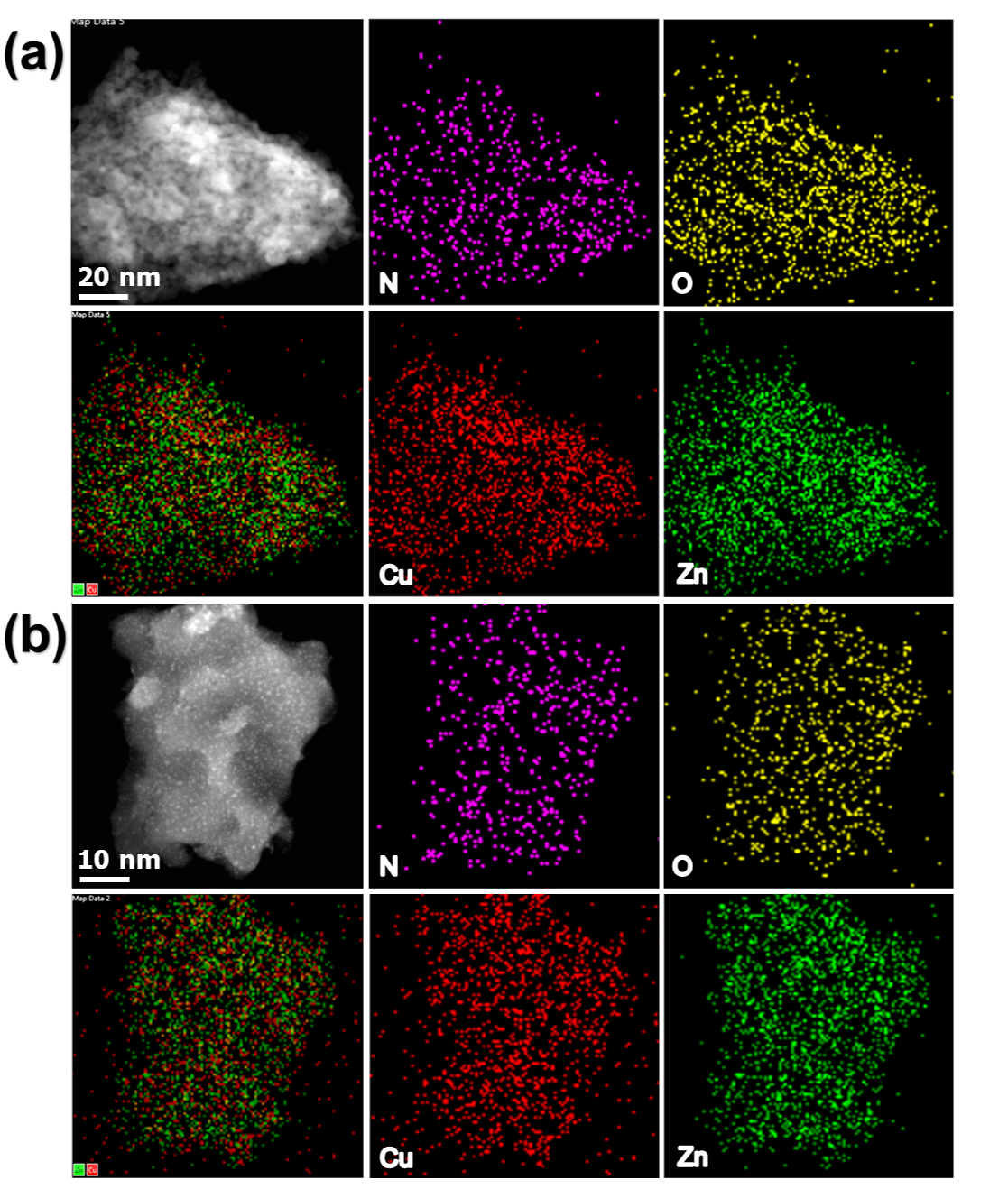
**

**Figure S37.** HAADF-STEM and EDX element mapping images for (a) HKUST-1/ZIF-8-1 and (b) Cu@ZIF-8-1.

**
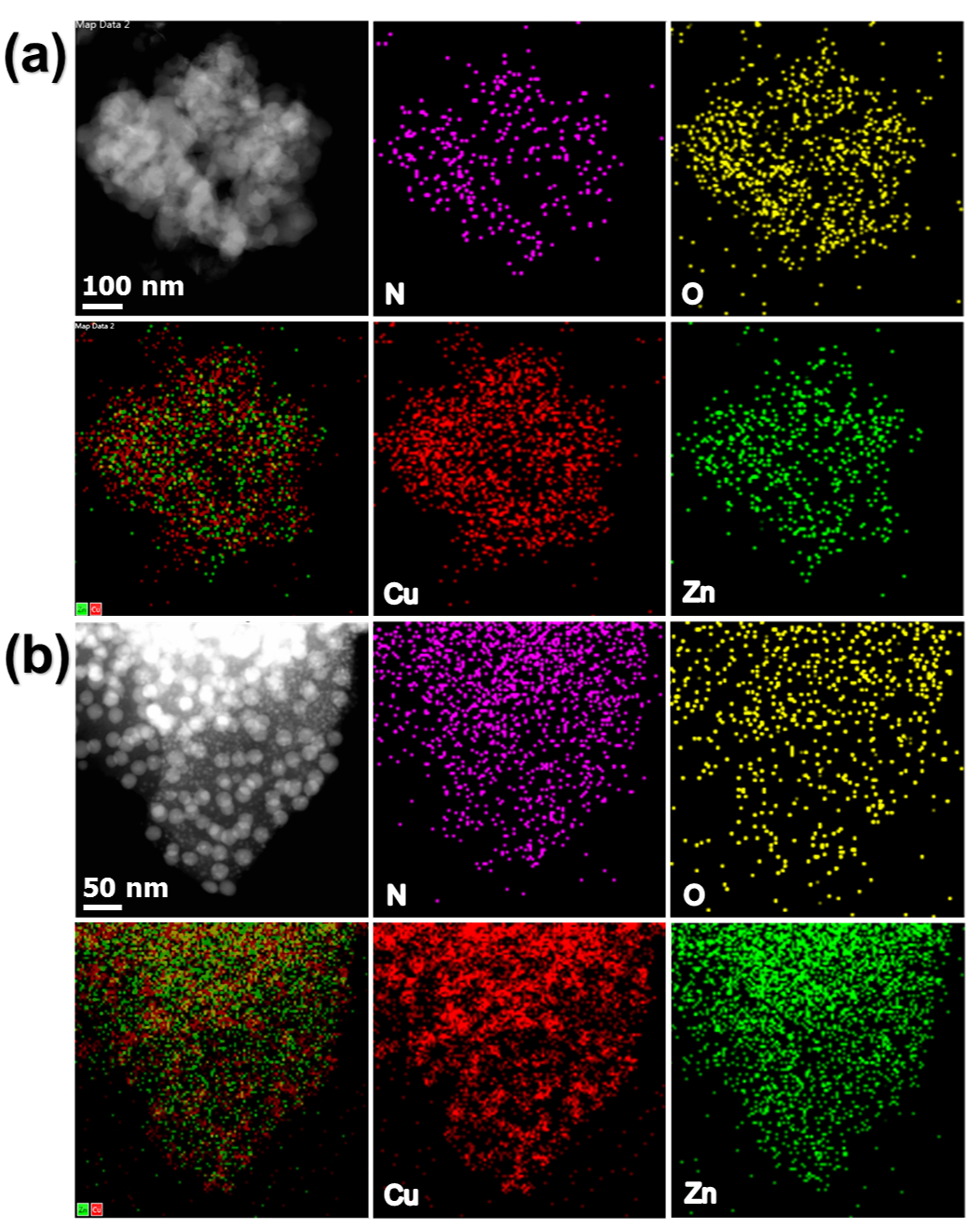
**

**Figure S38.** HAADF-STEM and EDX element mapping images for (a) HKUST-1/ZIF-8-2 and (b) Cu@ZIF-8-2.


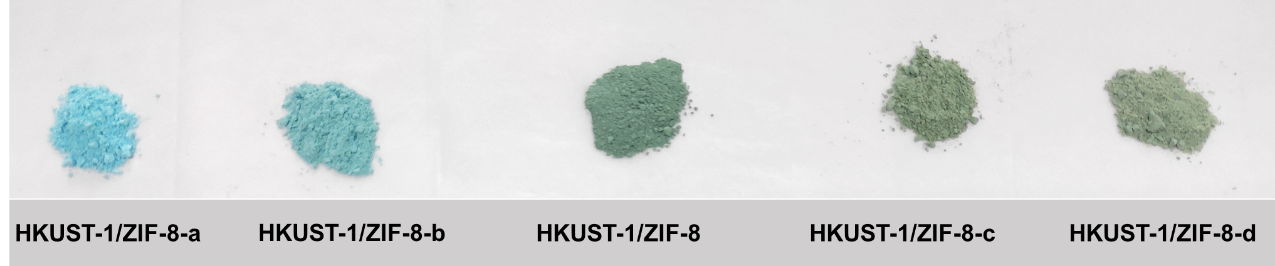


**Figure S39.** Photographs of HKUST-1/ZIF-8-a, HKUST-1/ZIF-8-b, HKUST-1/ZIF-8, HKUST-1/ZIF-8-c and HKUST-1/ZIF-8-d. The dual-MOFs with different proportion of organic ligands were synthesized by the same procedure of HKUST-1/ZIF-8, except for the 2-methylimidazole content. The feed ratios of 2-methylimidazole to H_3_BTC are 1.5, 3, 6, 9 and 12 in the samples of HKUST-1/ZIF-8-a, HKUST-1/ZIF-8-b, HKUST-1/ZIF-8, HKUST-1/ZIF-8-c and HKUST-1/ZIF-8-d, respectively.


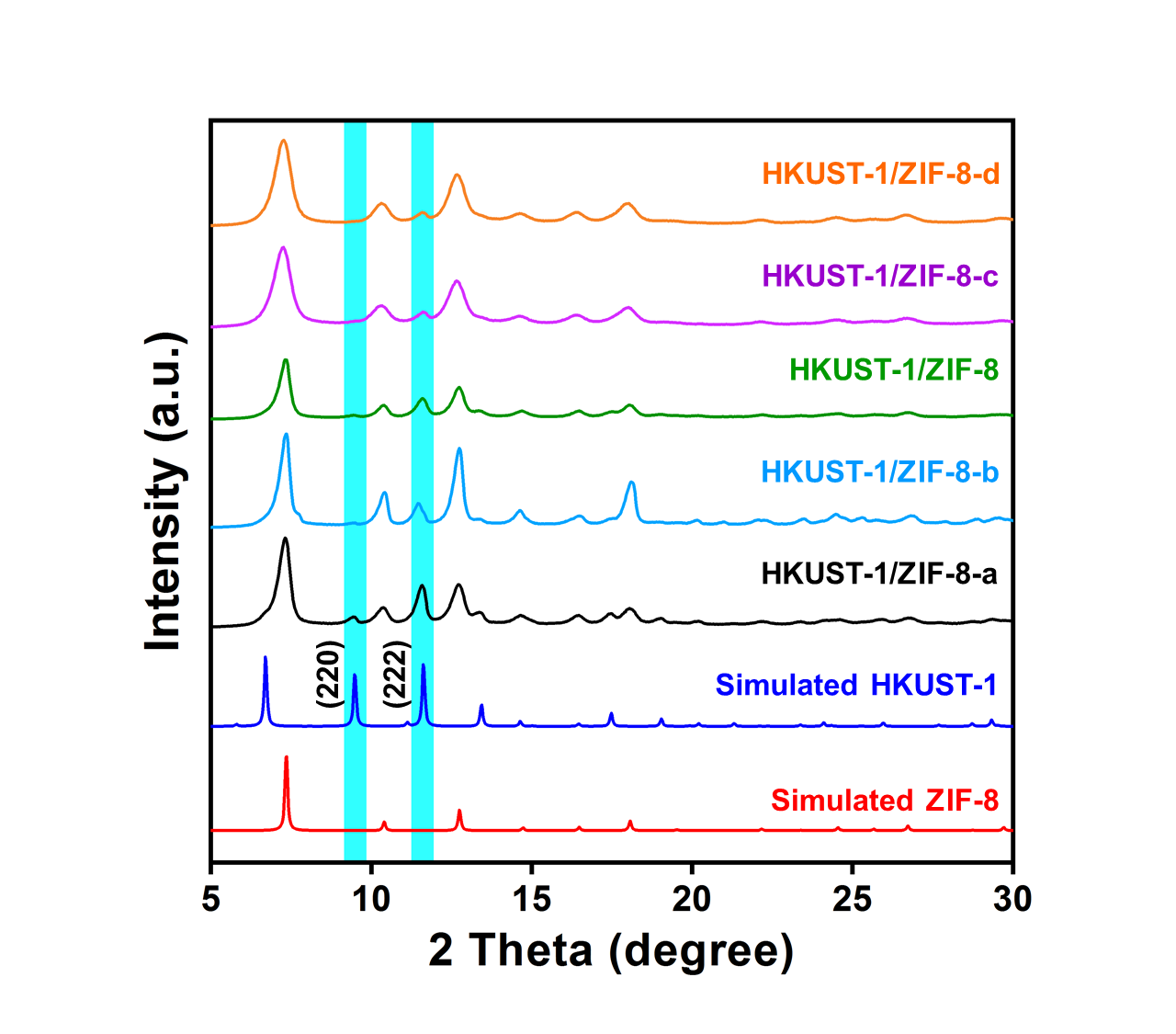


**Figure S40.** PXRD patterns of HKUST-1/ZIF-8 with different proportions of organic ligands.

**
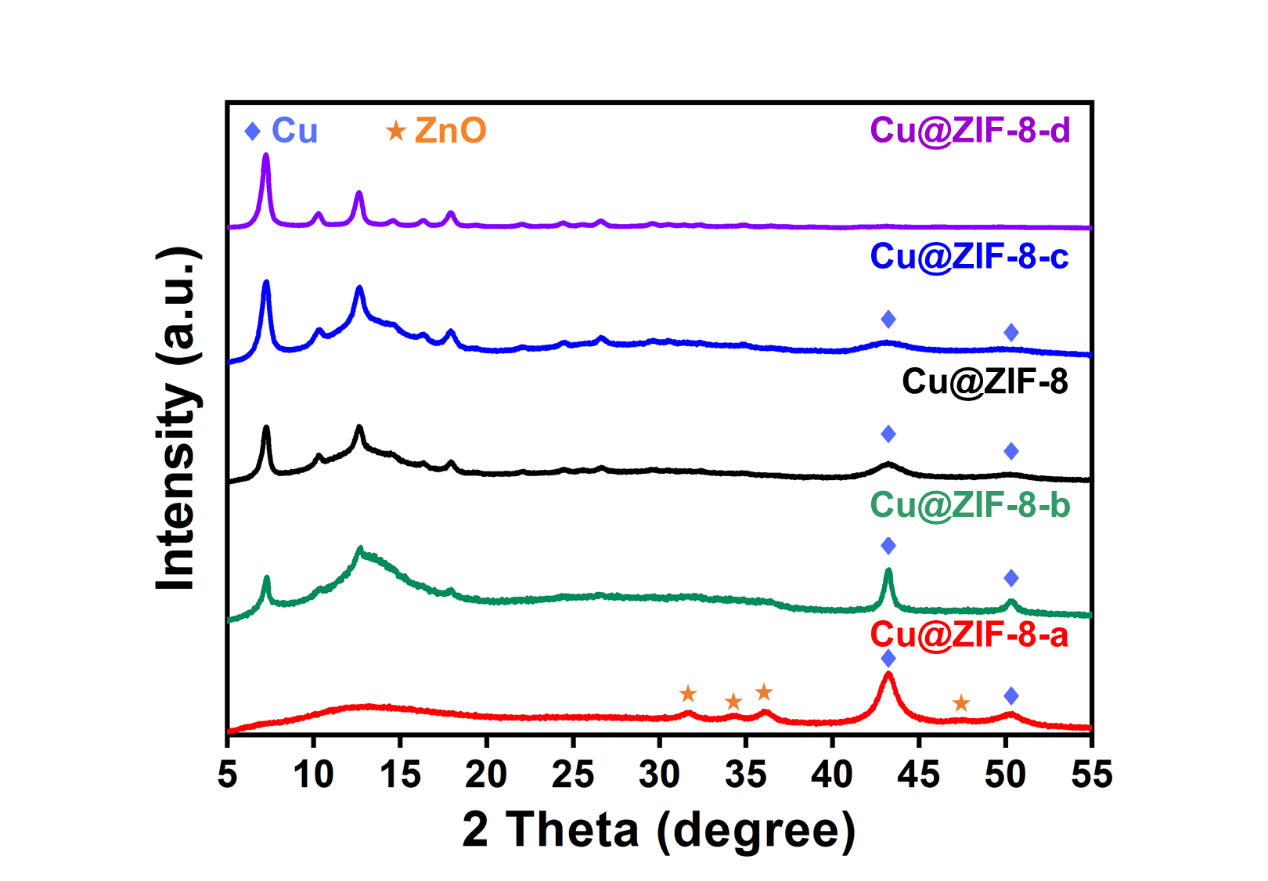
**

**Figure S41.** PXRD patterns of Cu@ZIF-8 with different proportions of organic ligands. Cu@ZIF-8-a, Cu@ZIF-8-b, Cu@ZIF-8-c and Cu@ZIF-8-d are the 350 ^o^C annealed products of HKUST-1/ZIF-8-a, HKUST-1/ZIF-8-b, HKUST-1/ZIF-8-c and HKUST-1/ZIF-8-d, respectively.


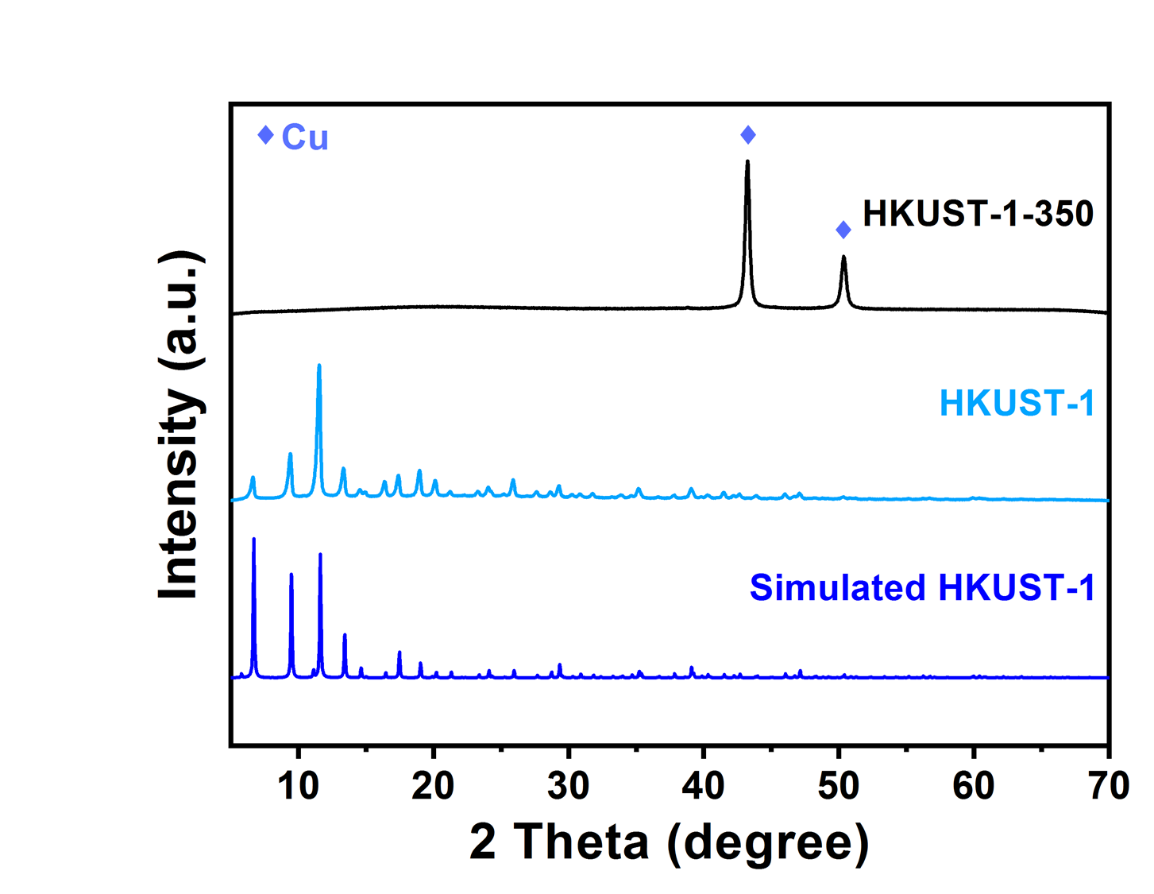


**Figure S42.** Comparison of the PXRD patterns of HKUST-1 and HKUST-1-350. HKUST-1-350 is the annealed product of HKUST-1 at 350 ^o^C.

**
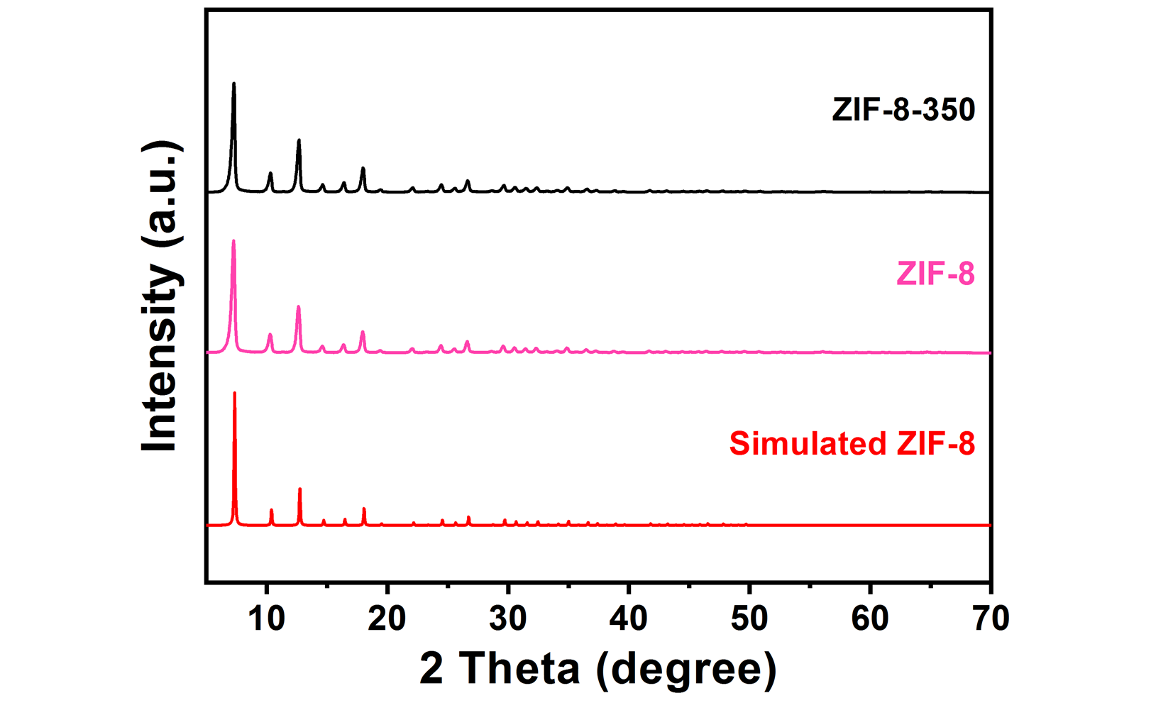
**

**Figure S43.** Comparison of the PXRD patterns of ZIF-8 and ZIF-8-350. ZIF-8-350 is the annealed product of ZIF-8 at 350 ^o^C.

**
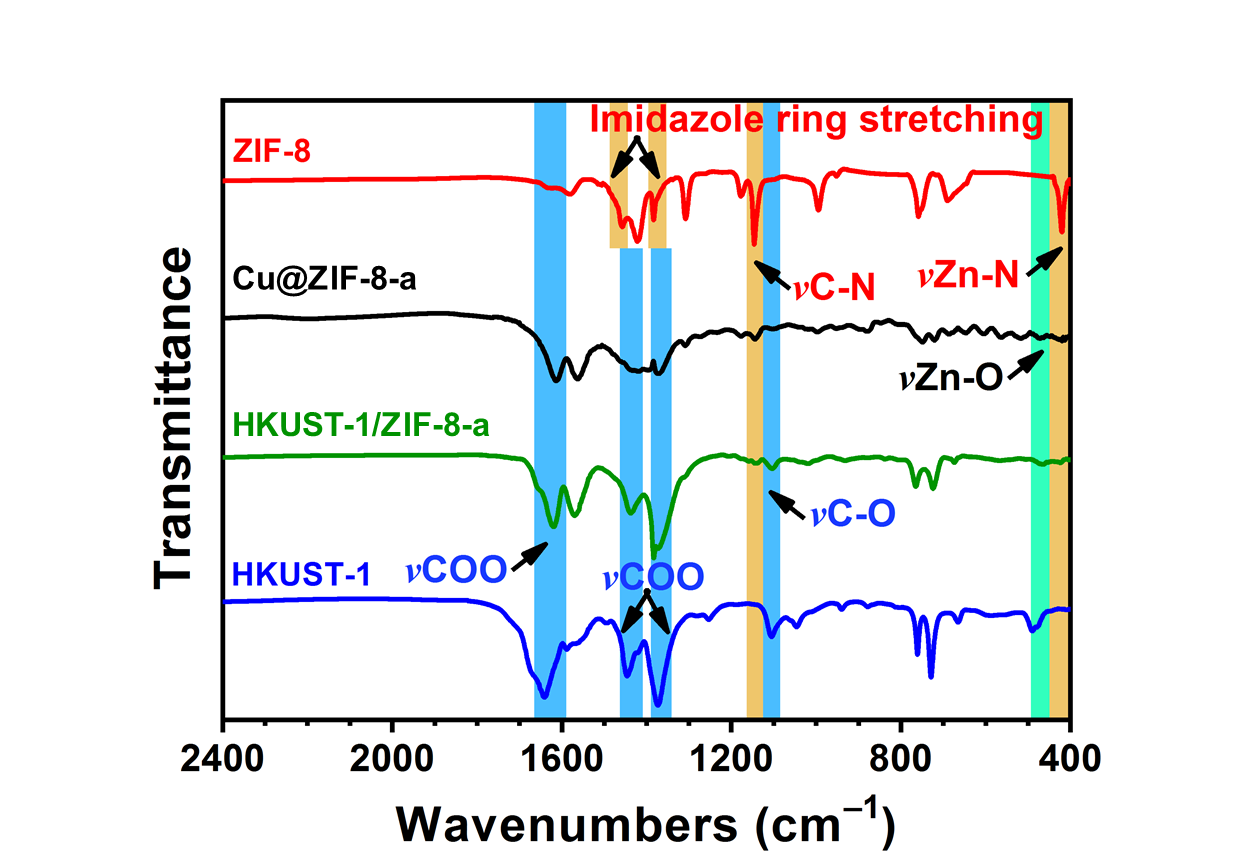
**

**Figure S44.** Comparison of the FT-IR spectra of HKUST-1/ZIF-8-a and Cu@ZIF-8-a.


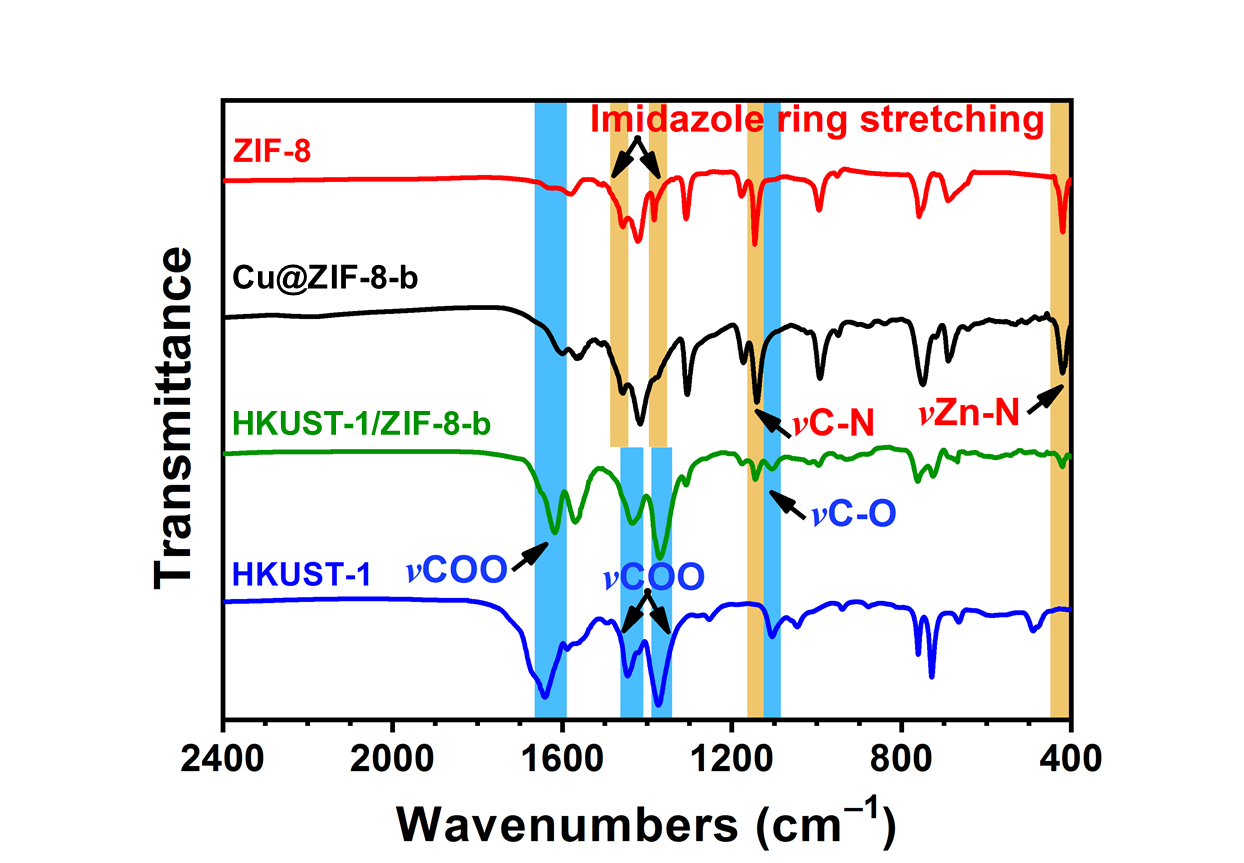


**Figure S45.** Comparison of the FT-IR spectra of HKUST-1/ZIF-8-b and Cu@ZIF-8-b.

**
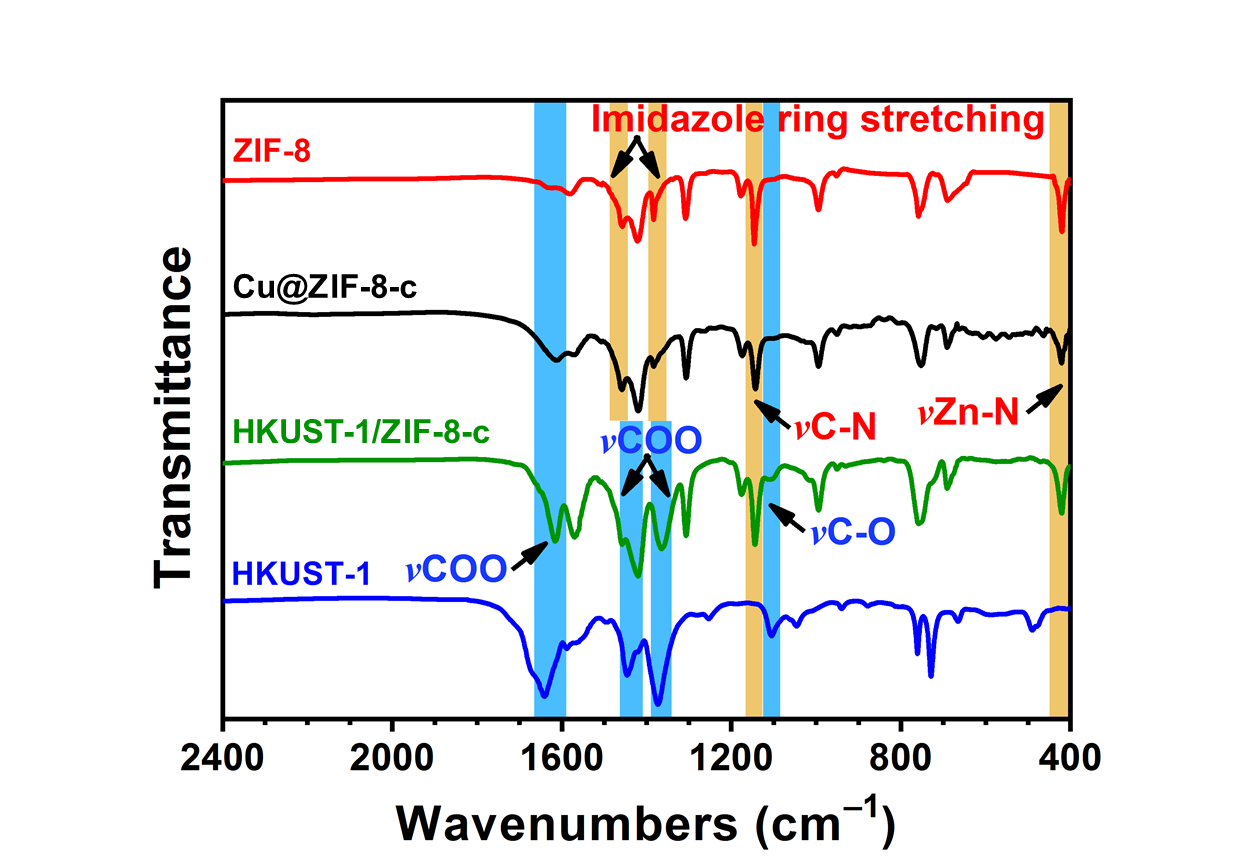
**

**Figure S46.** Comparison of the FT-IR spectra of HKUST-1/ZIF-8-c and Cu@ZIF-8-c.


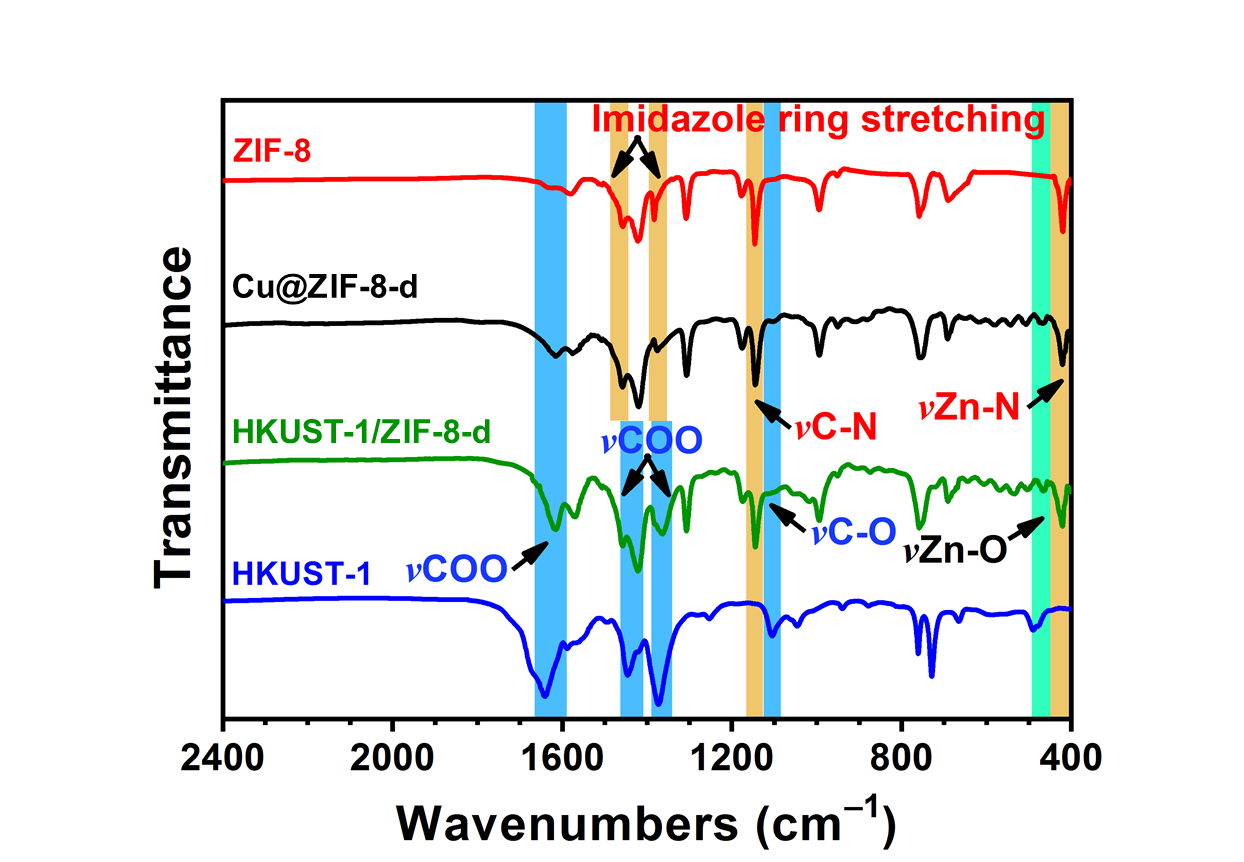


**Figure S47.** Comparison of the FT-IR spectra of HKUST-1/ZIF-8-d and Cu@ZIF-8-d.


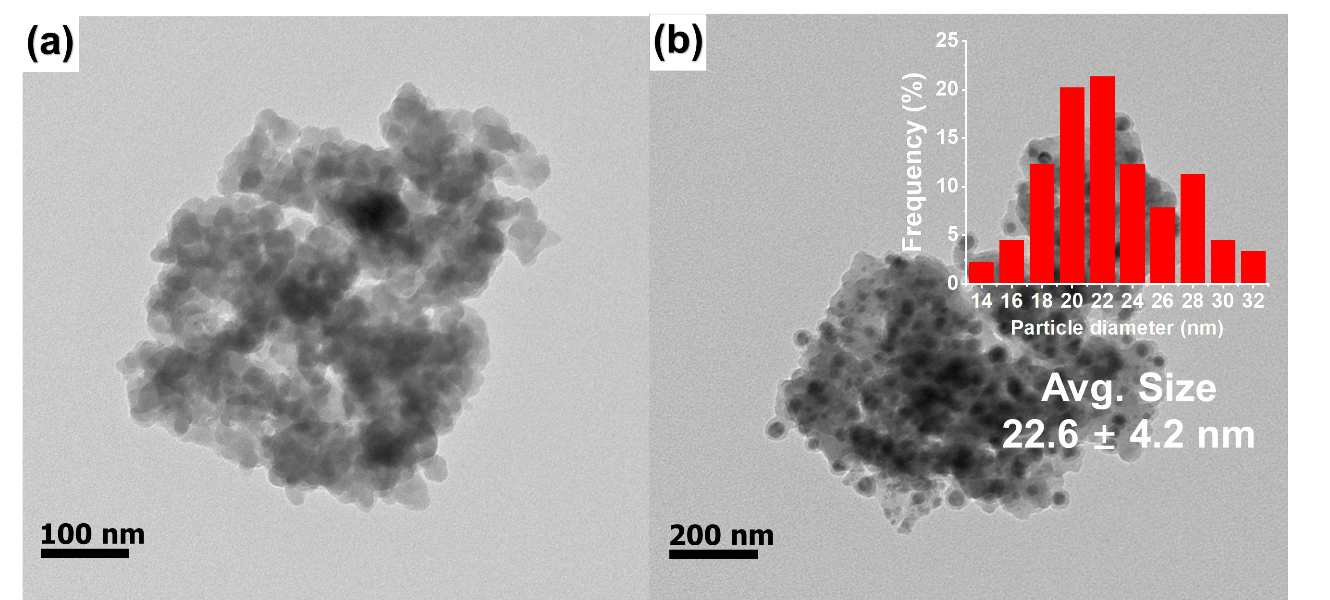


**Figure S48.** TEM images of (a) HKUST-1/ZIF-8-a and (b) Cu@ZIF-8-a. The inset shows a histogram of metal particle size distribution.


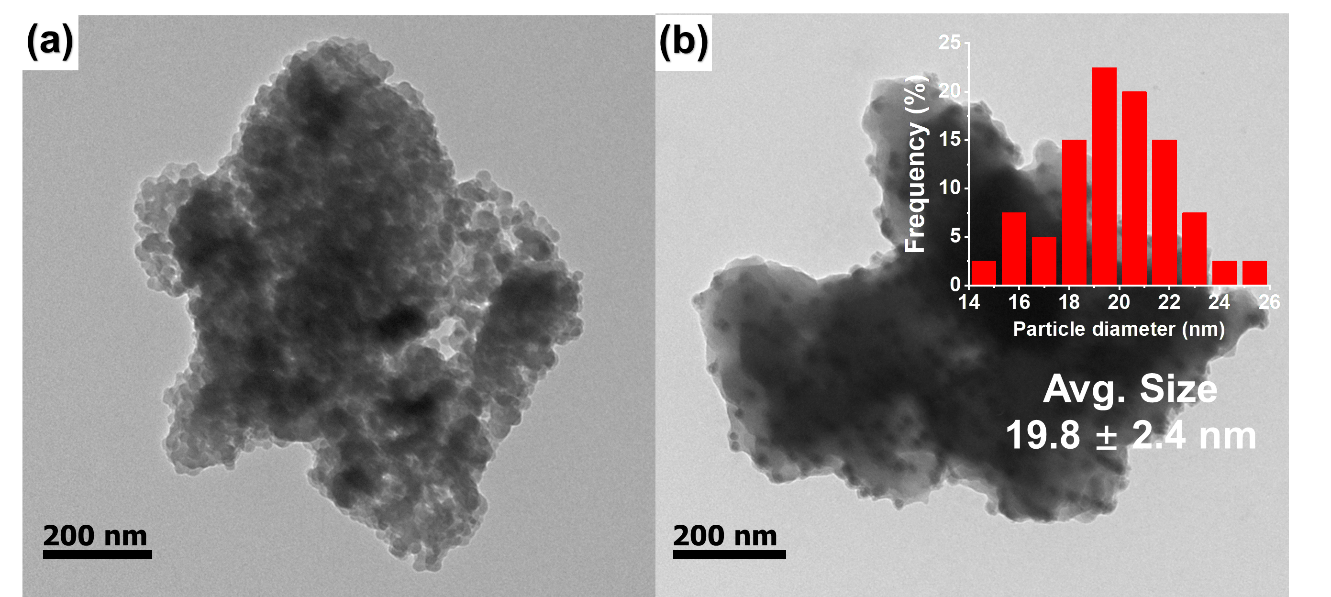


**Figure S49.** TEM images of (a) HKUST-1/ZIF-8-b and (b) Cu@ZIF-8-b. The inset shows a histogram of metal particle size distribution.


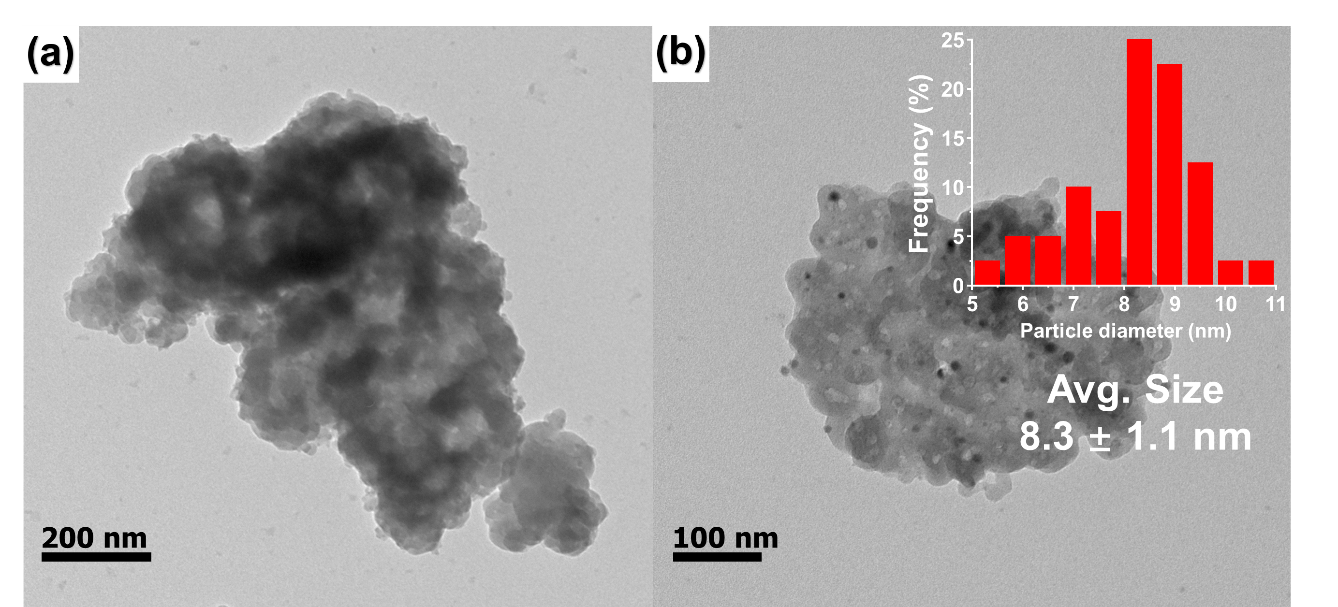


**Figure S50.** TEM images of (a) HKUST-1/ZIF-8-c and (b) Cu@ZIF-8-c. The inset shows a histogram of metal particle size distribution.


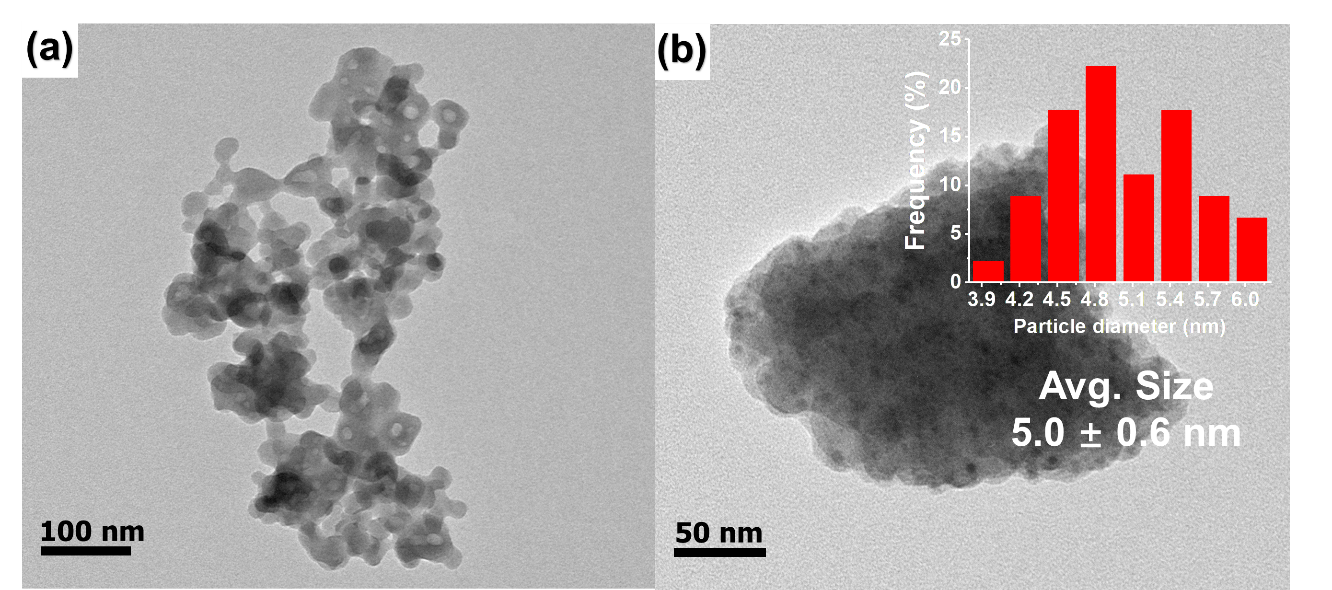


**Figure S51.** TEM images of (a) HKUST-1/ZIF-8-d and (b) Cu@ZIF-8-d. The inset shows a histogram of metal particle size distribution.


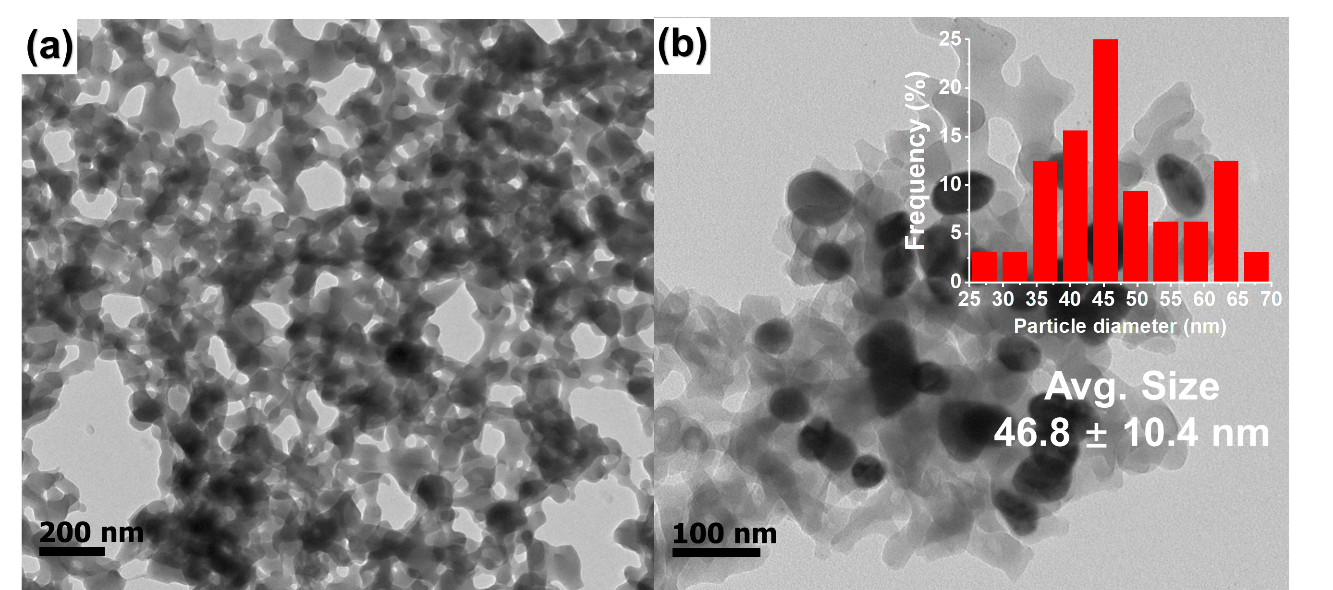


**Figure S52.** TEM images of (a) HKUST-1 and (b) HKUST-1-350. The inset shows a histogram of metal particle size distribution.


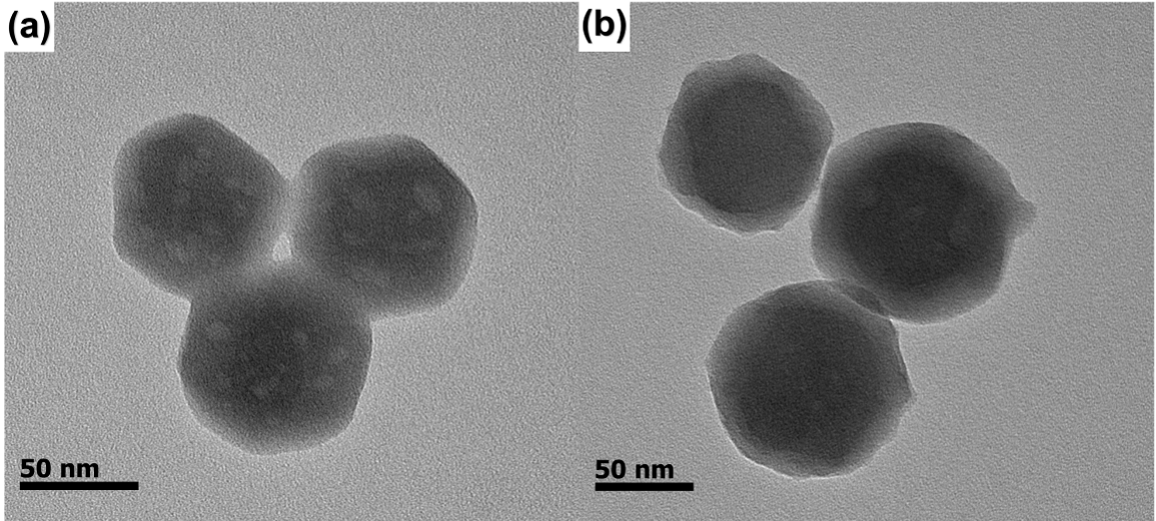


**Figure S53.** TEM images of (a) ZIF-8 and (b) ZIF-8-350.


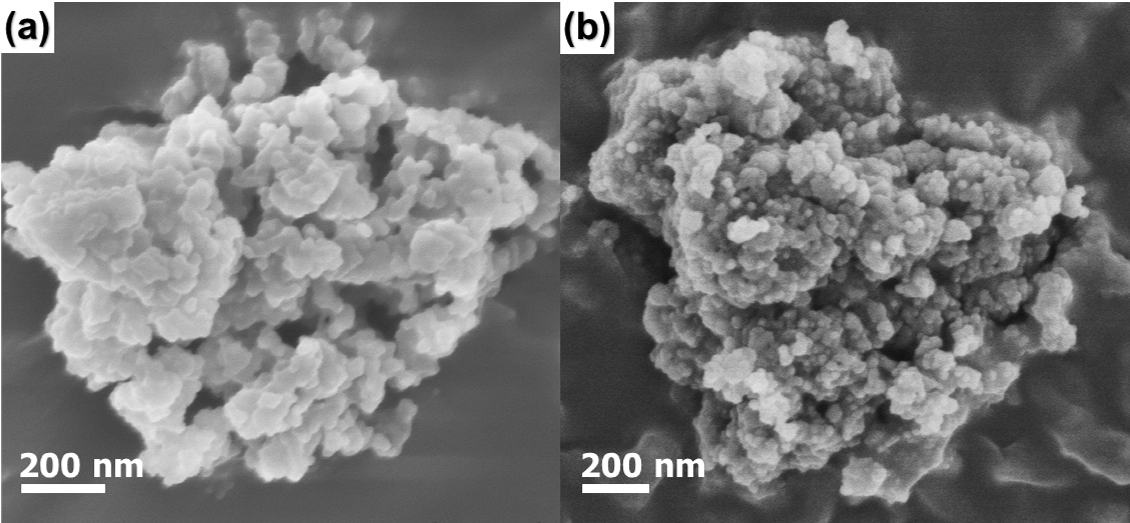


**Figure S54.** SEM images of (a) HKUST-1/ZIF-8-a and (b) Cu@ZIF-8-a.

**
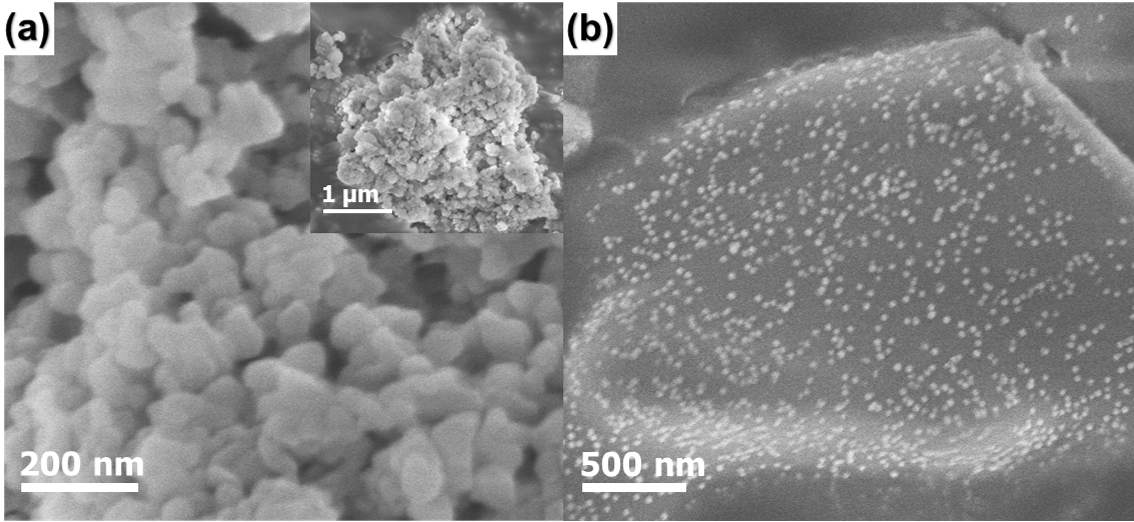
**

**Figure S55.** SEM images of (a) HKUST-1/ZIF-8-b and (b) Cu@ZIF-8-b. The inset shows a low-magnification SEM image.

**
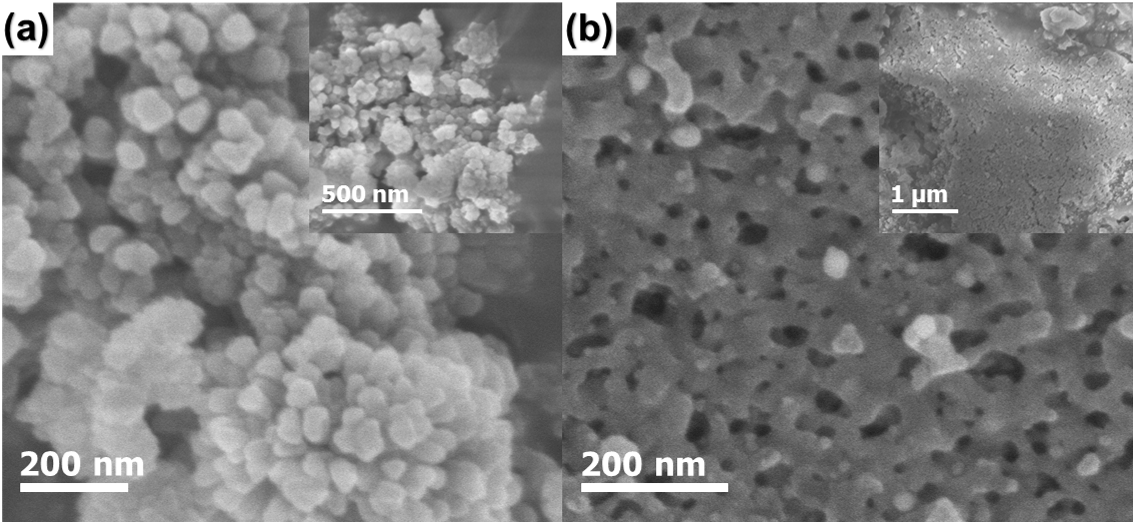
**

**Figure S56.** SEM images of (a) HKUST-1/ZIF-8-c and (b) Cu@ZIF-8-c. The insets show the low-magnification SEM images.


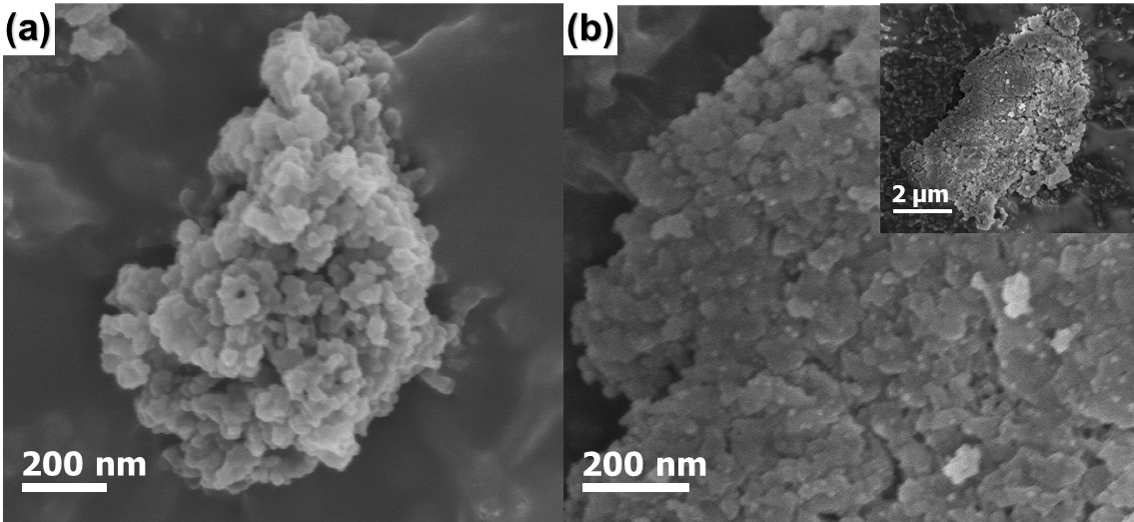


**Figure S57.** SEM images of (a) HKUST-1/ZIF-8-d and (b) Cu@ZIF-8-d. The inset shows a low-magnification SEM image.

**
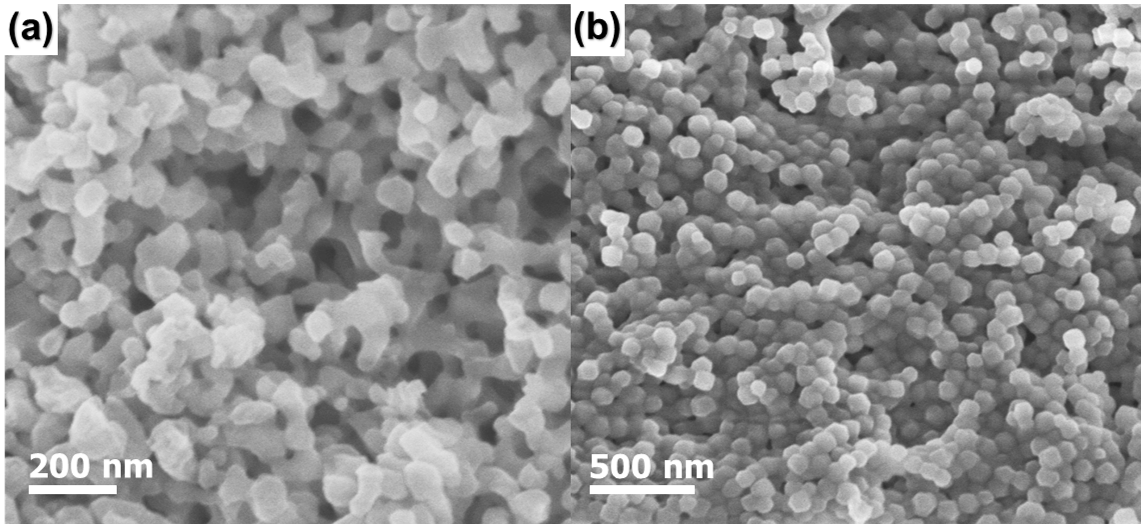
**

**Figure S58.** SEM images of (a) HKUST-1 and (b) HKUST-1-350.


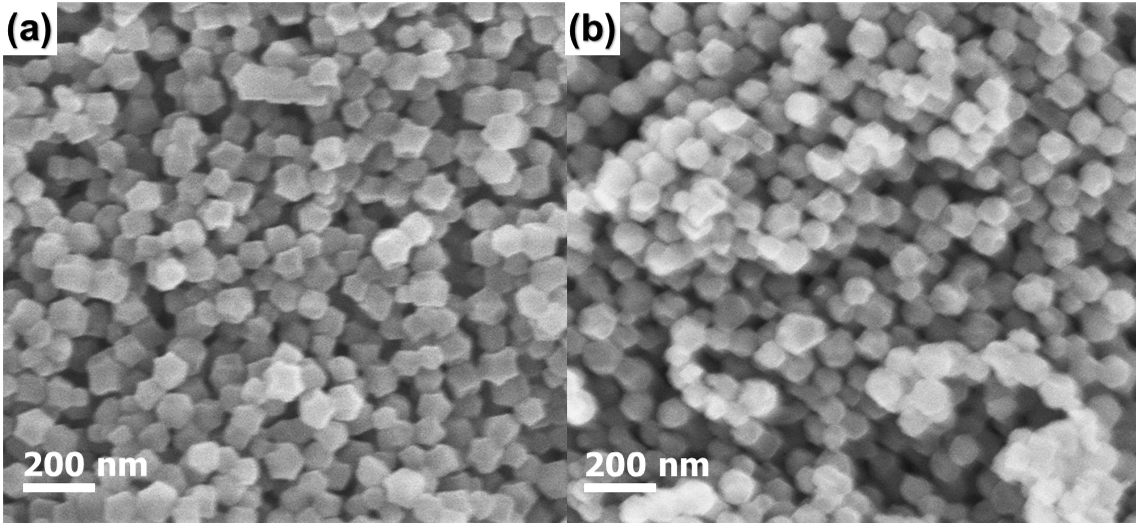


**Figure S59.** SEM images of (a) ZIF-8 and (b) ZIF-8-350.


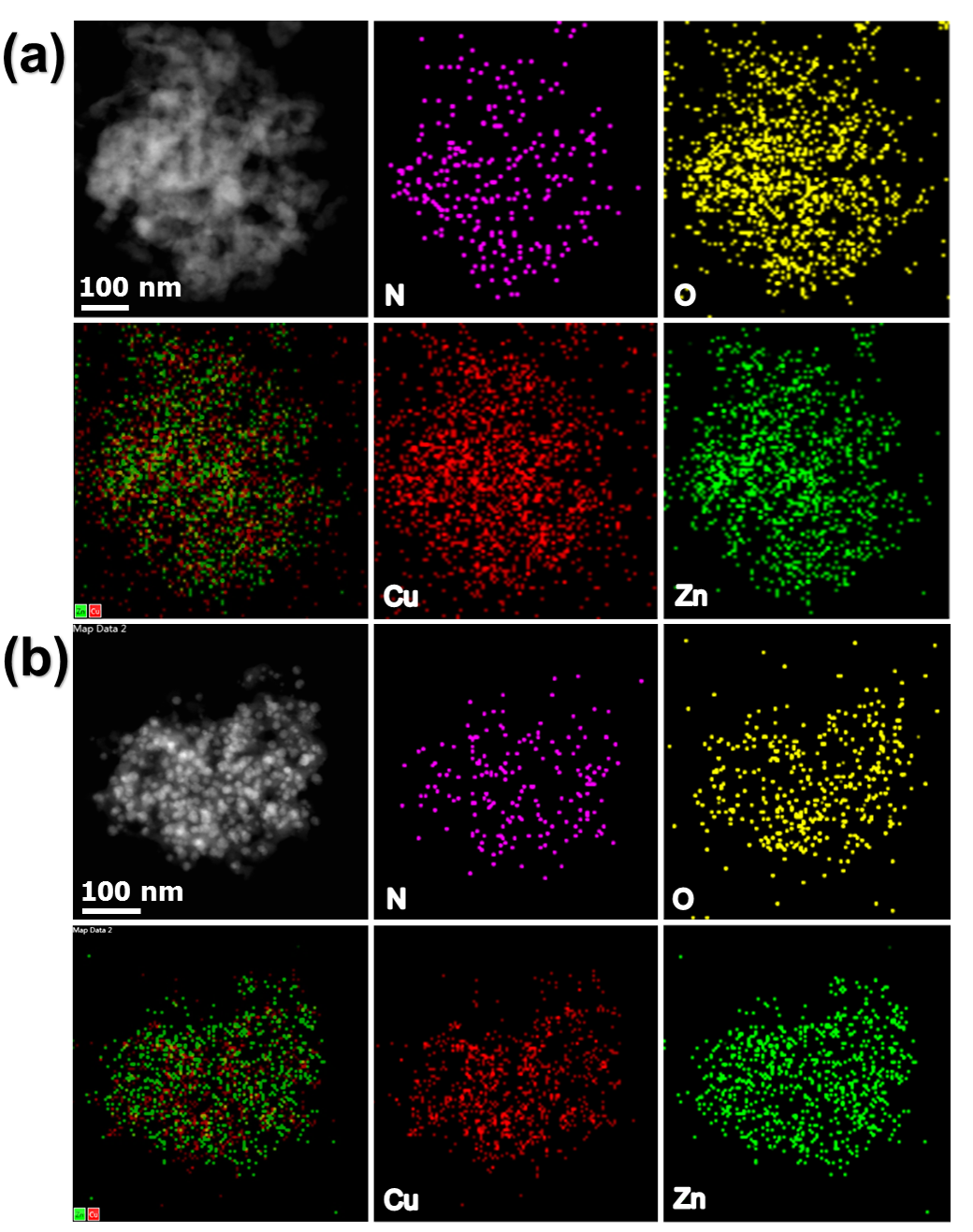


**Figure S60.** HAADF-STEM and EDX element mapping images of (a) HKUST-1/ZIF-8-a and (b) Cu@ZIF-8-a.


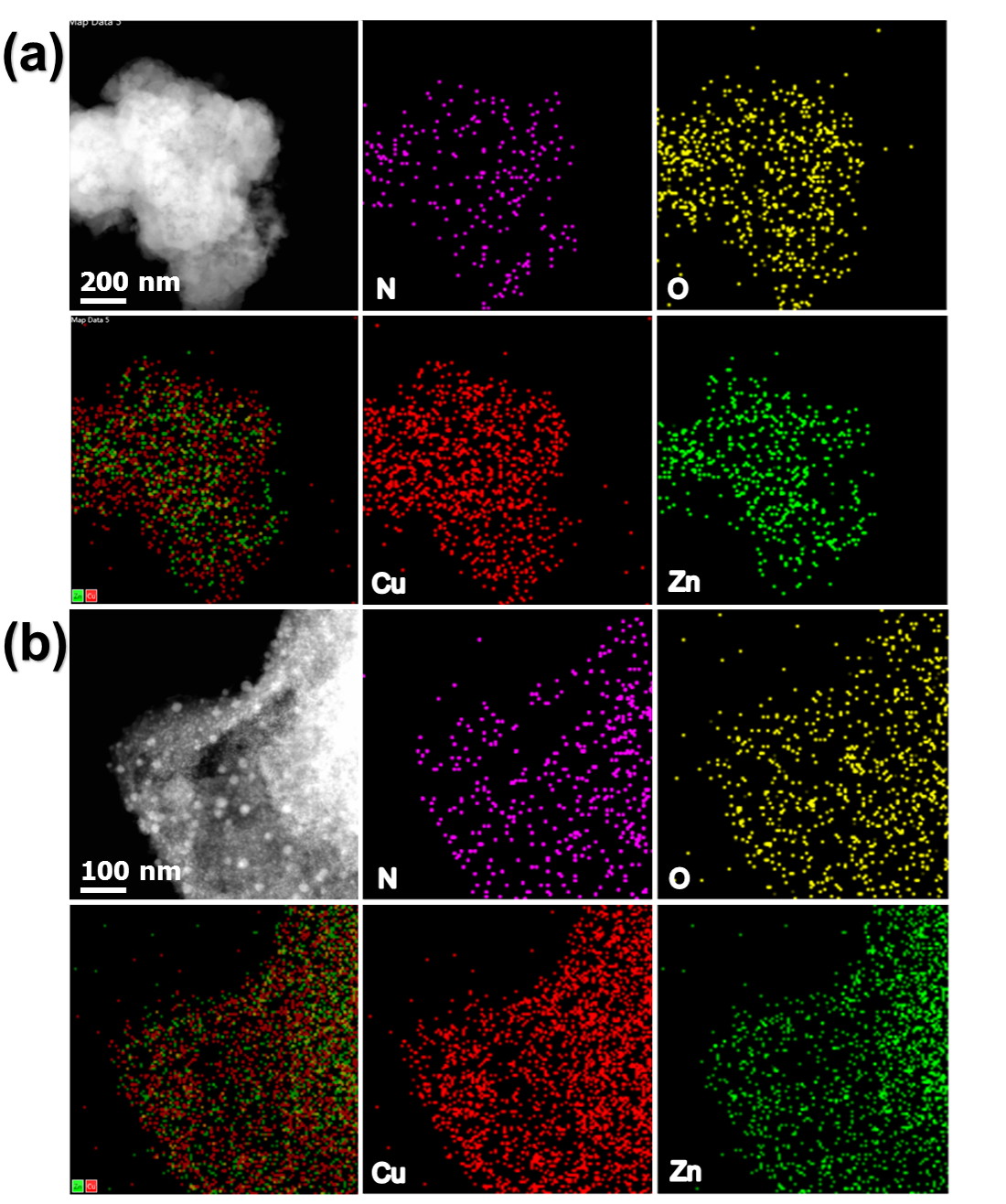


**Figure S61.** HAADF-STEM and EDX element mapping images of (a) HKUST-1/ZIF-8-b and (b) Cu@ZIF-8-b.


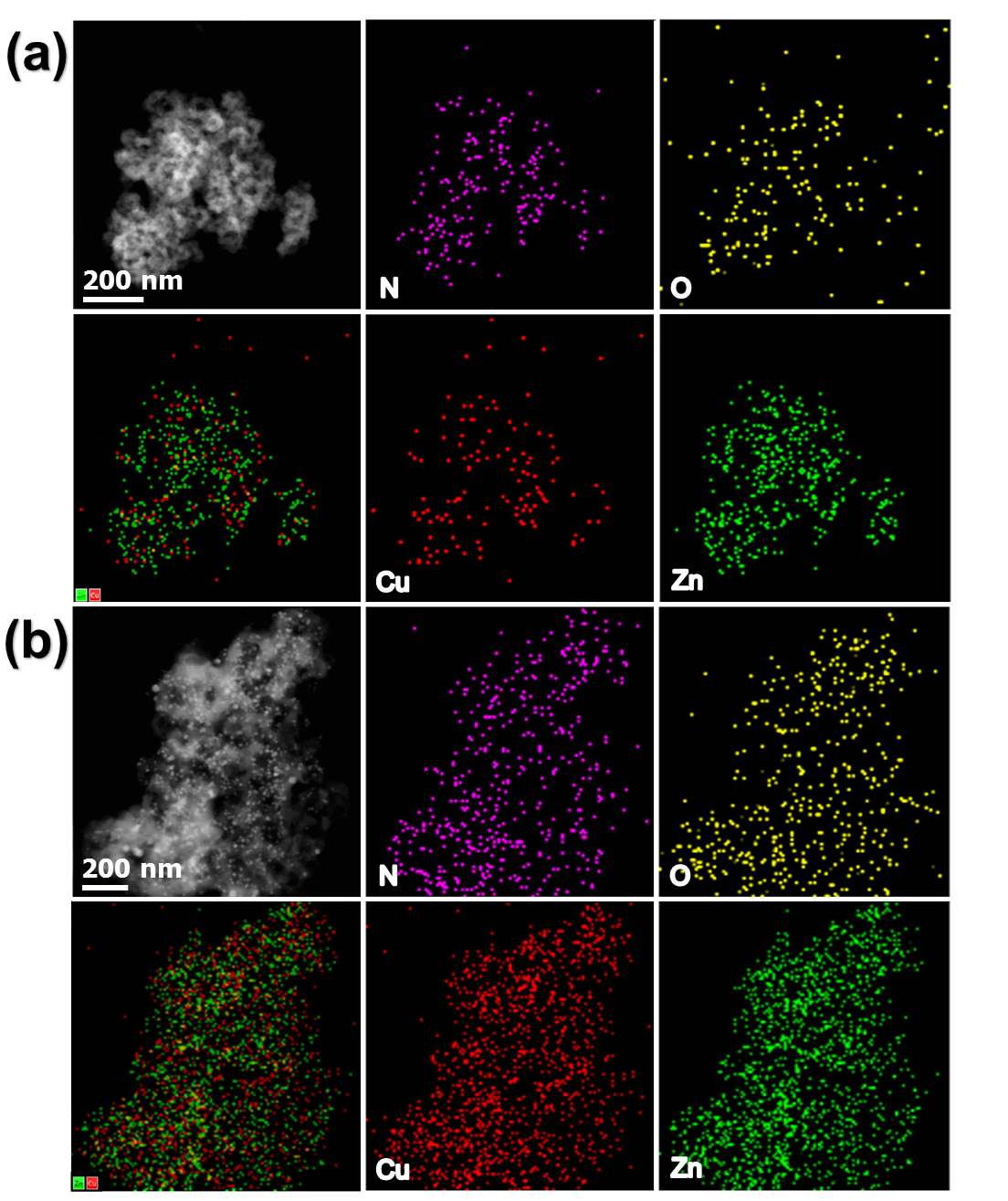


**Figure S62.** HAADF-STEM and EDX element mapping images of (a) HKUST-1/ZIF-8-c and (b) Cu@ZIF-8-c.


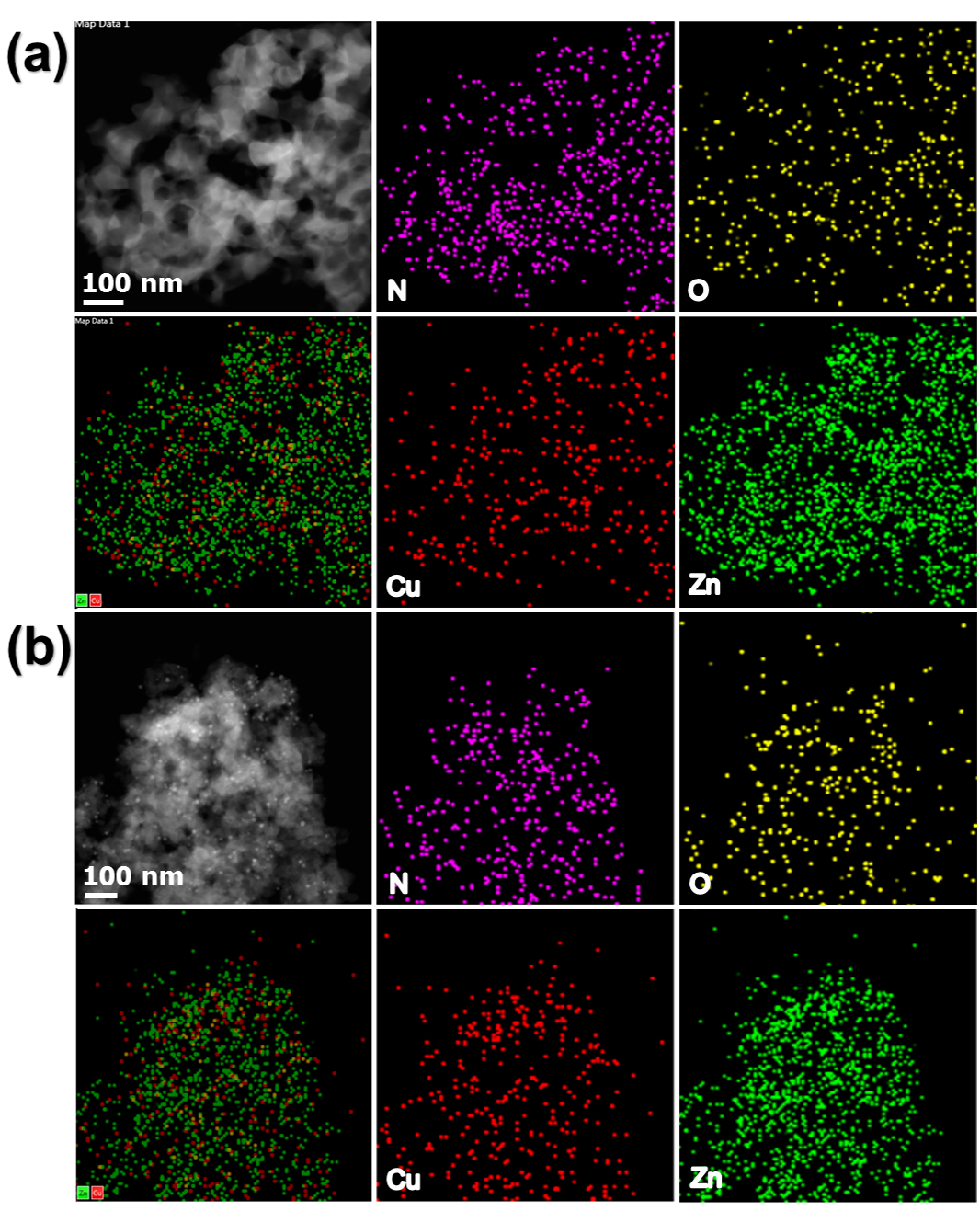


**Figure S63.** HAADF-STEM and EDX element mapping images of (a) HKUST-1/ZIF-8-d and (b) Cu@ZIF-8-d.


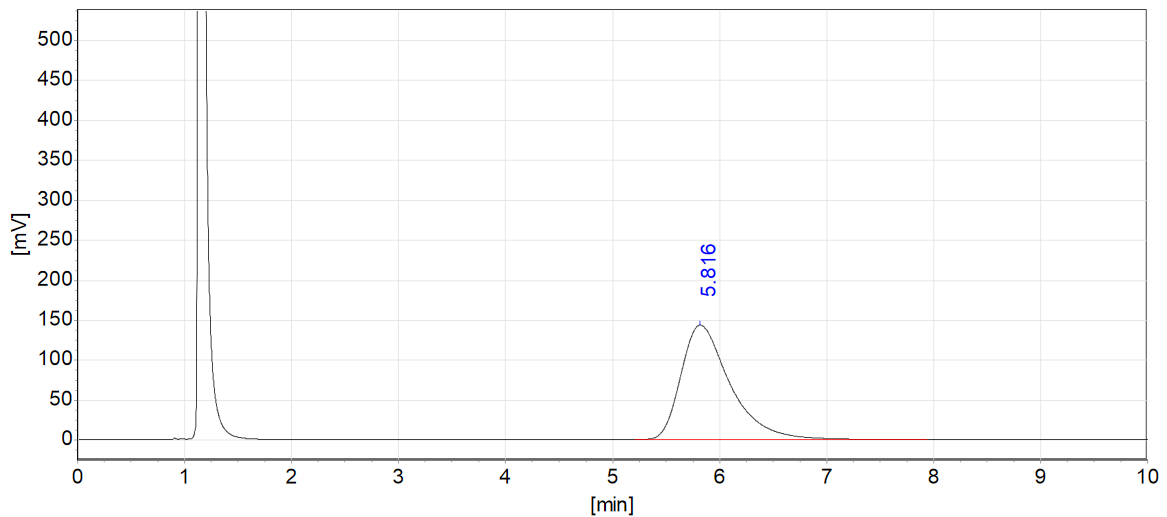


**Figure S64.** GC trace of phenylacetylene.


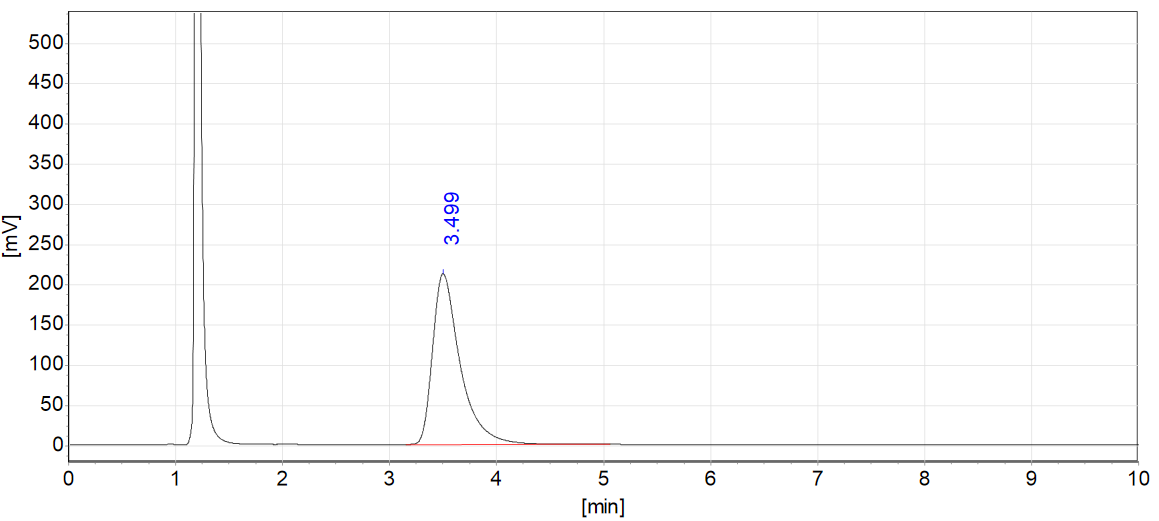


**Figure S65.** GC trace of styrene, the product for semihydrogenation of phenylacetylene.


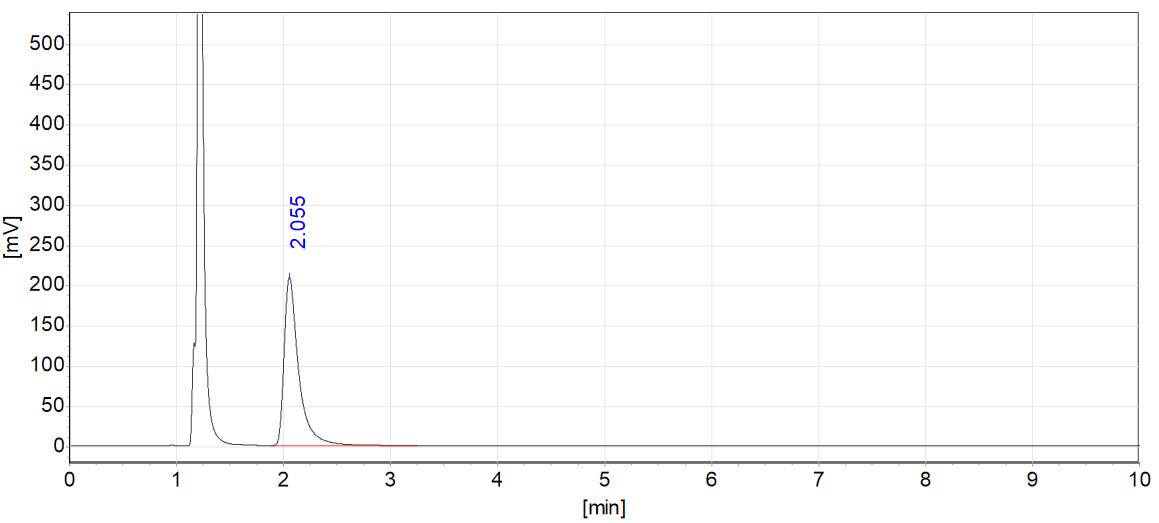


**Figure S66.** GC trace of ethylbenzene, the product for overhydrogenation of phenylacetylene.


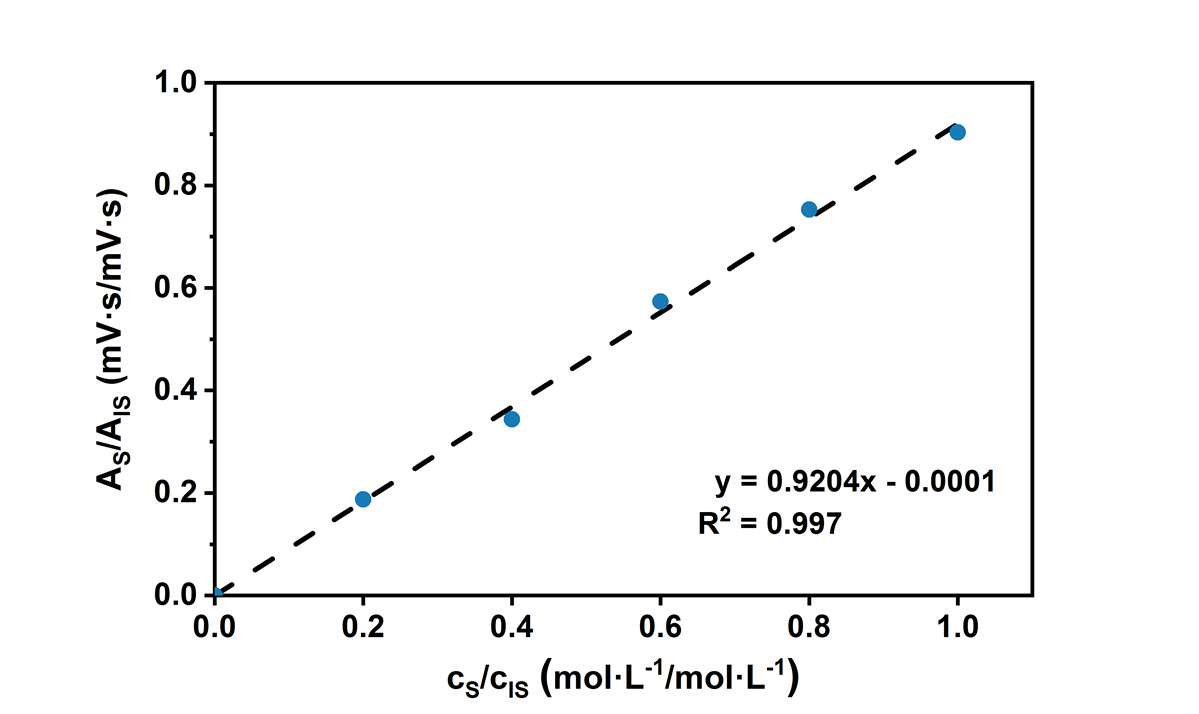


**Figure S67.** GC calibration curve for styrene. S: styrene, IS: internal standard, A: area, c: concentration.


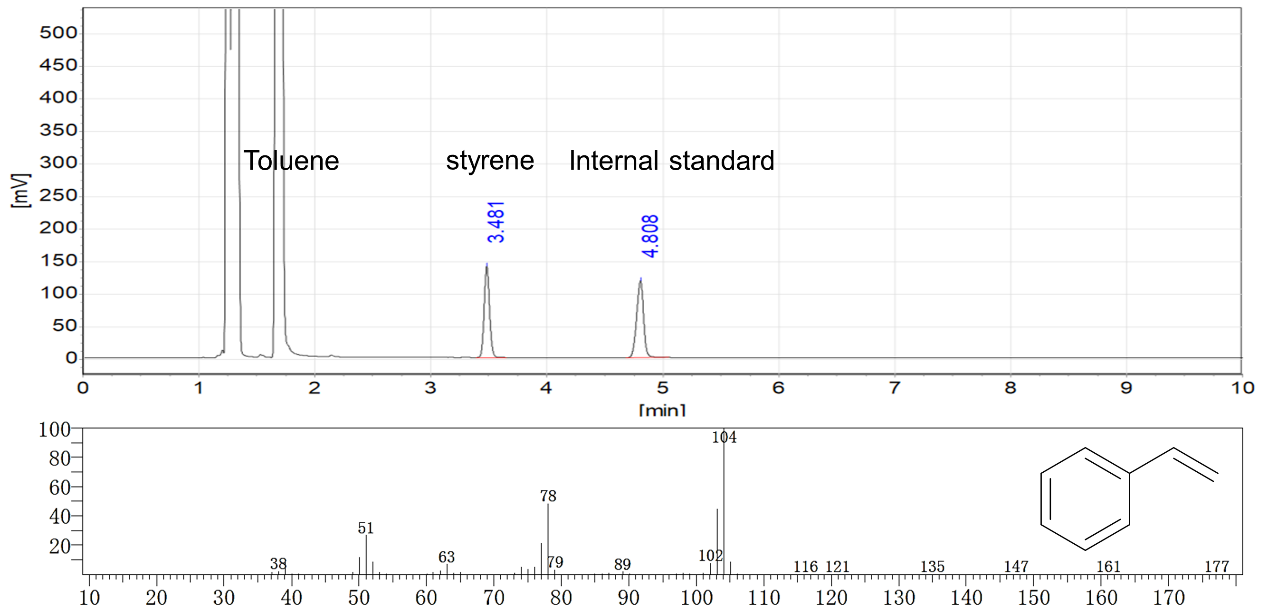


**Figure S68.** GC-MS data for semihydrogenation of phenylacetylene catalyzed by Cu@ZIF-8.

| Peak# | Retention time (min) | Height (mV) | Peak area (mV·s) | Peak area (%) |
| --- | --- | --- | --- | --- |
| 1 | 3.481 | 140132 | 449979 | 47.90362 |
| 2 | 4.808 | 118027 | 489363 | 52.09638 |
| Total |  | 258159 | 939342 | 100 |


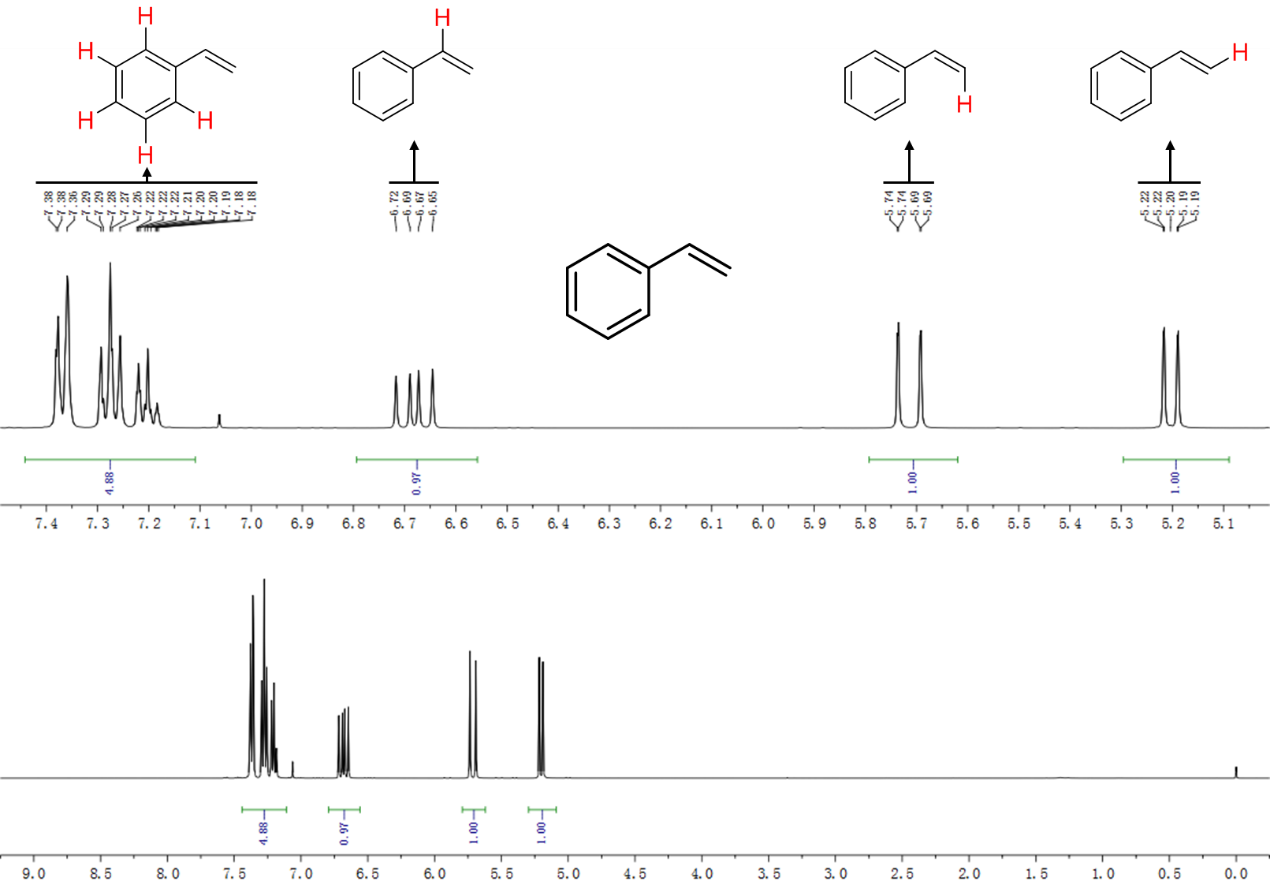


**Figure S69.** ^1^H NMR spectrum of styrene, the product for semihydrogenation of phenylacetylene.


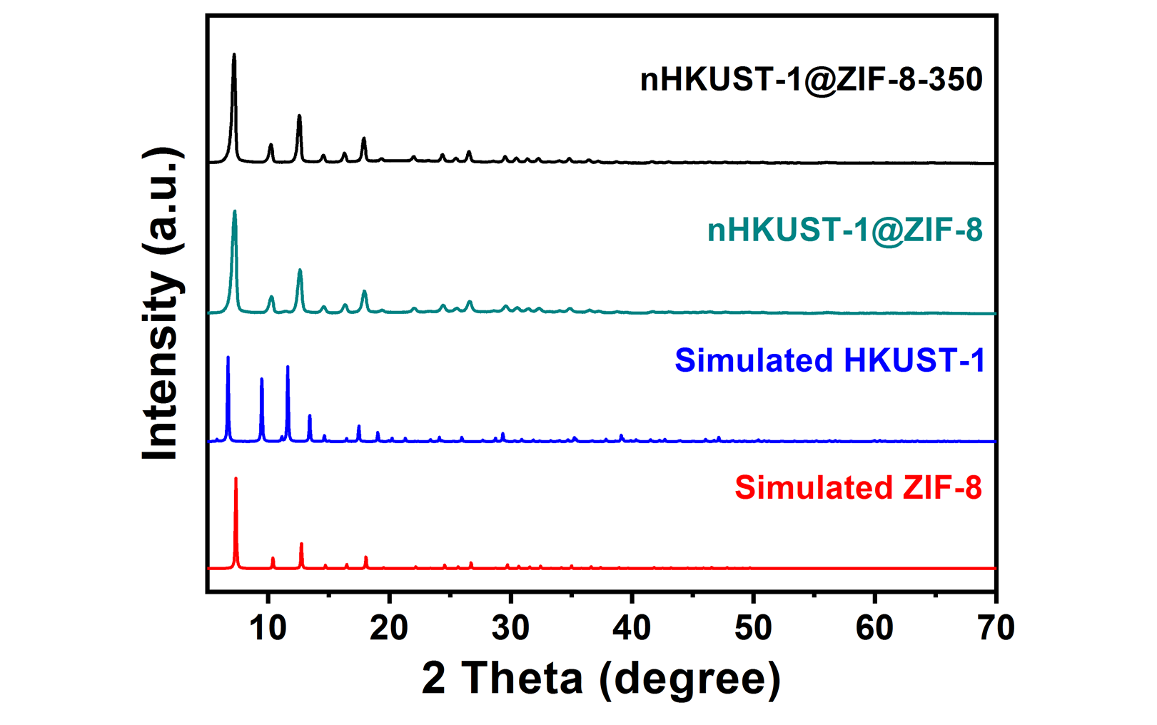


**Figure S70.** PXRD patterns of core-shell nHKUST-1@ZIF-8 and the annealed product nHKUST-1@ZIF-8-350 at 350 ^o^C.


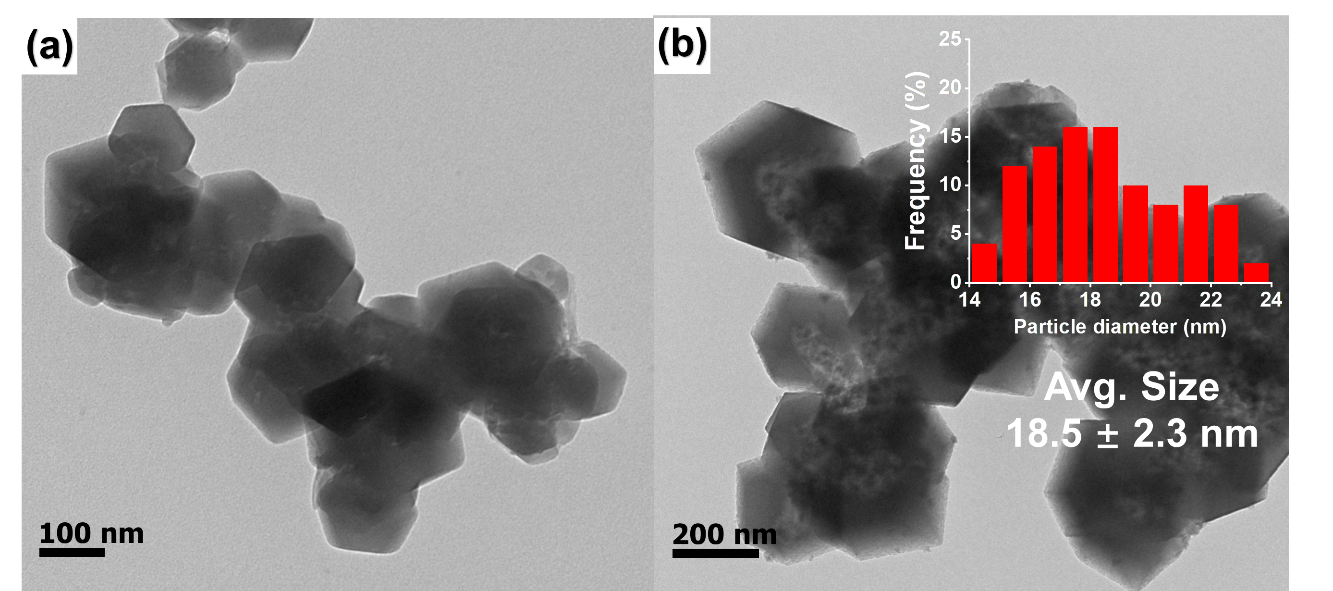


**Figure S71.** TEM images of (a) nHKUST-1@ZIF-8 and (b) nHKUST-1@ZIF-8-350. The inset shows a histogram of metal particle size distribution.


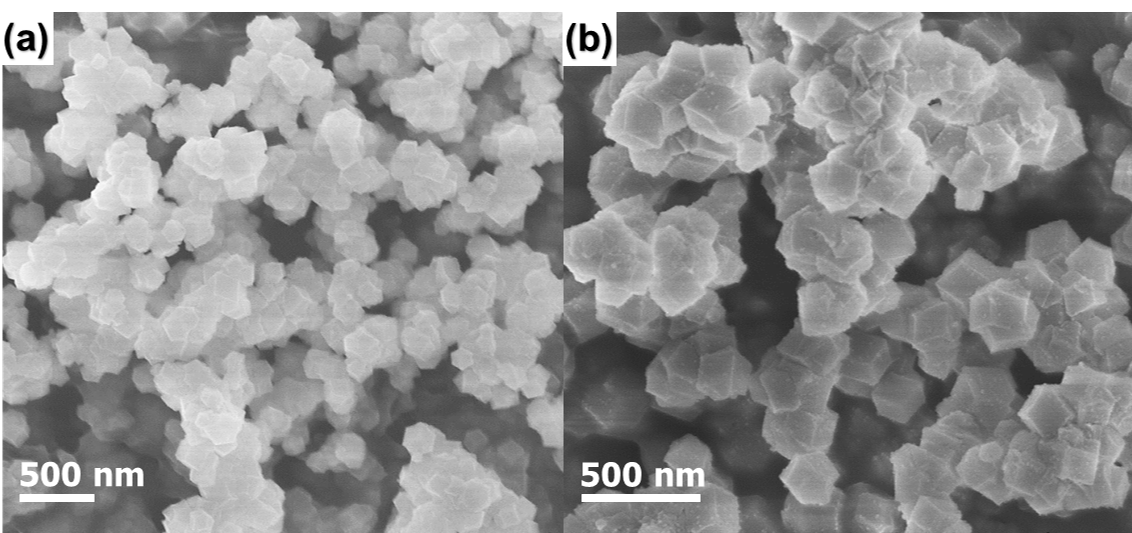


**Figure S72.** SEM images of (a) nHKUST-1@ZIF-8 and (b) nHKUST-1@ZIF-8-350.

**
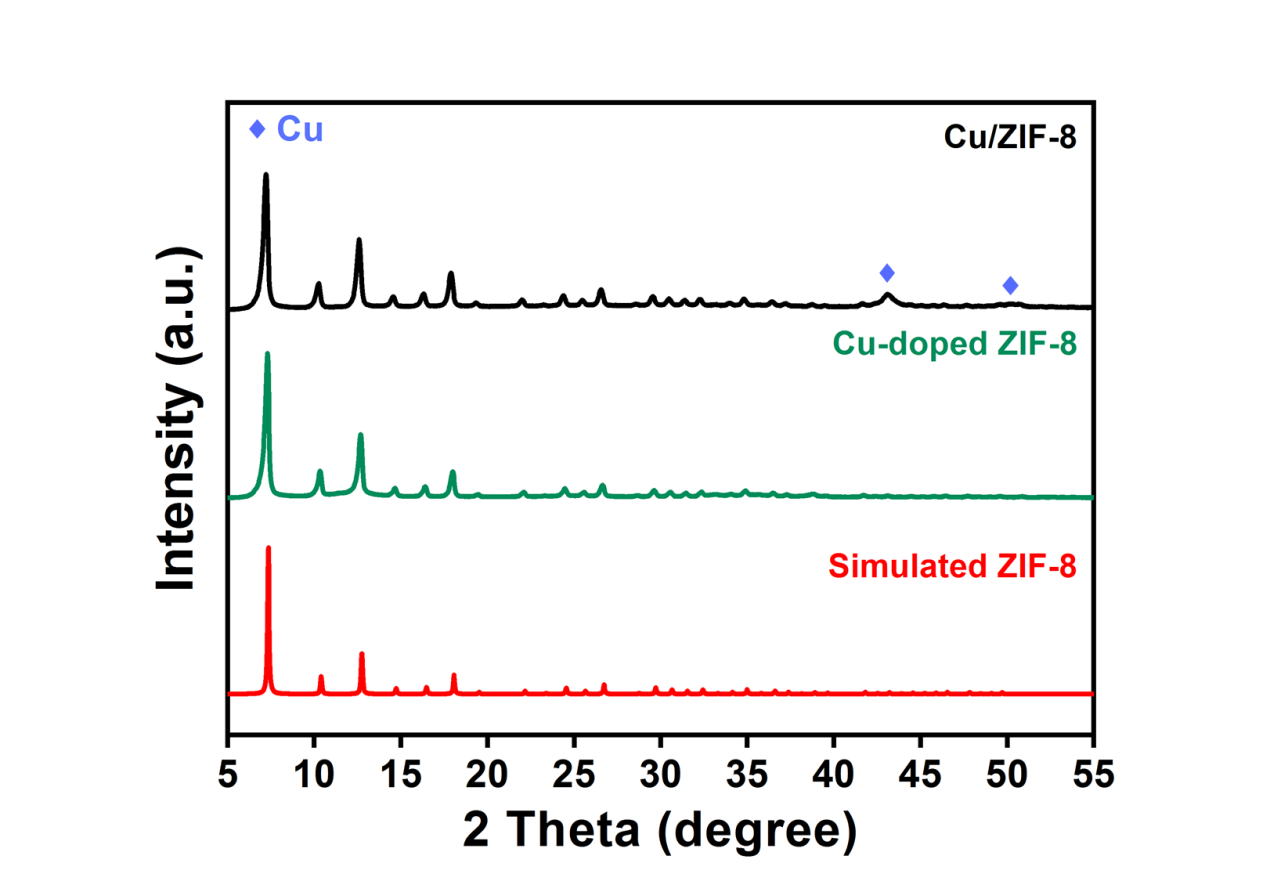
**

**Figure S73.** PXRD patterns of Cu-doped ZIF-8 and Cu/ZIF-8. Cu-doped ZIF-8 was prepared by conventional solution impregnation method for comparison. Cu/ZIF-8 is the annealed product of Cu-doped ZIF-8 at 350 ^o^C.


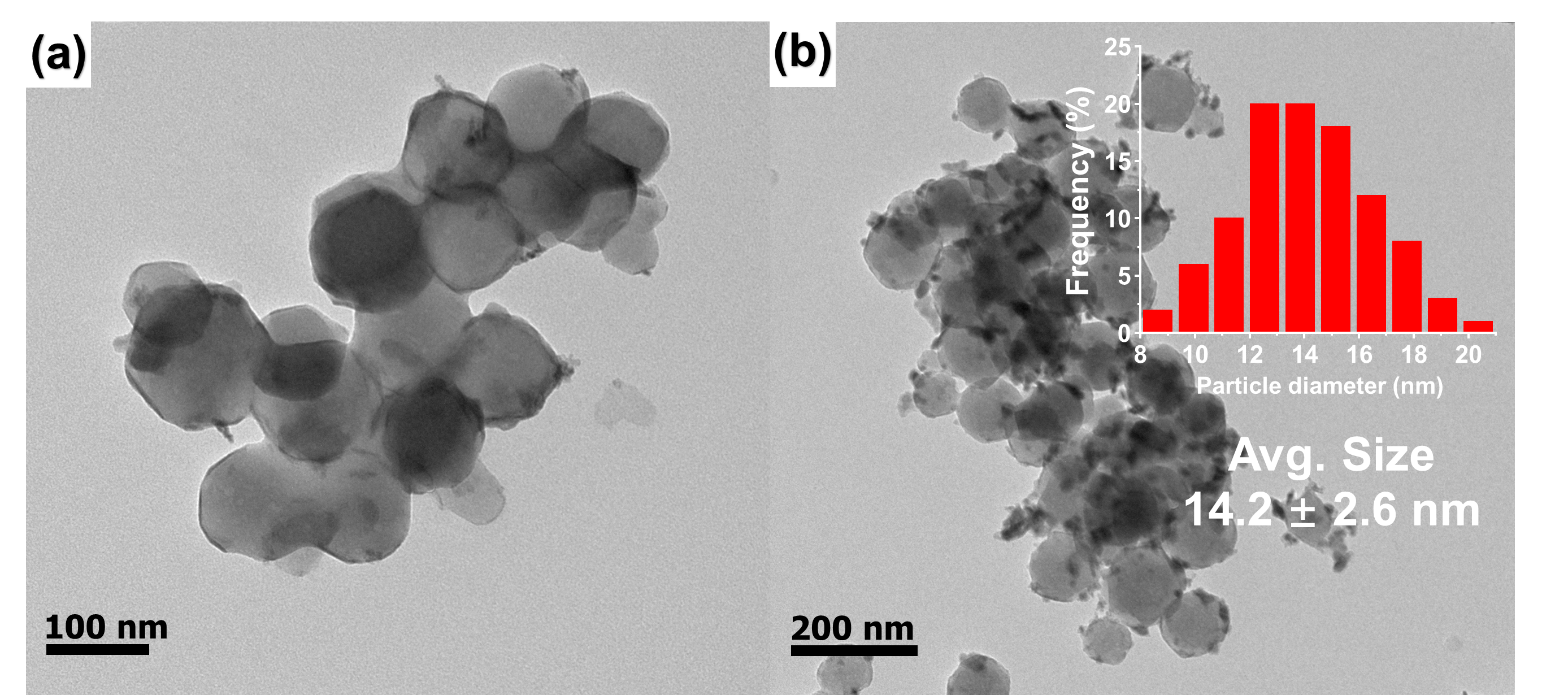


**Figure S74.** TEM images of (a) Cu-doped ZIF-8 and (b) Cu/ZIF-8. The inset shows a histogram of metal particle size distribution.


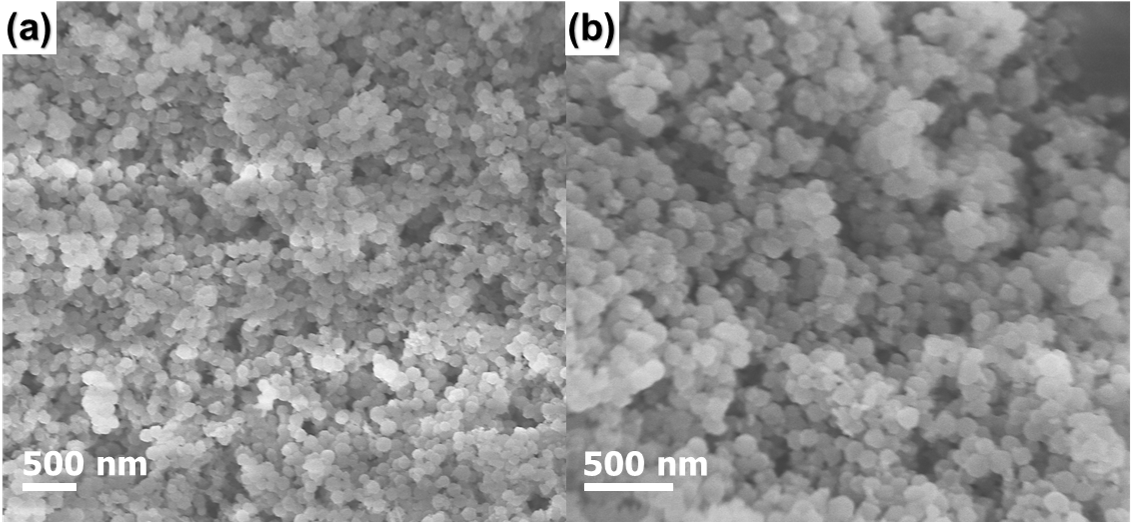


**Figure S75.** SEM images of (a) Cu-doped ZIF-8 and (b) Cu/ZIF-8.


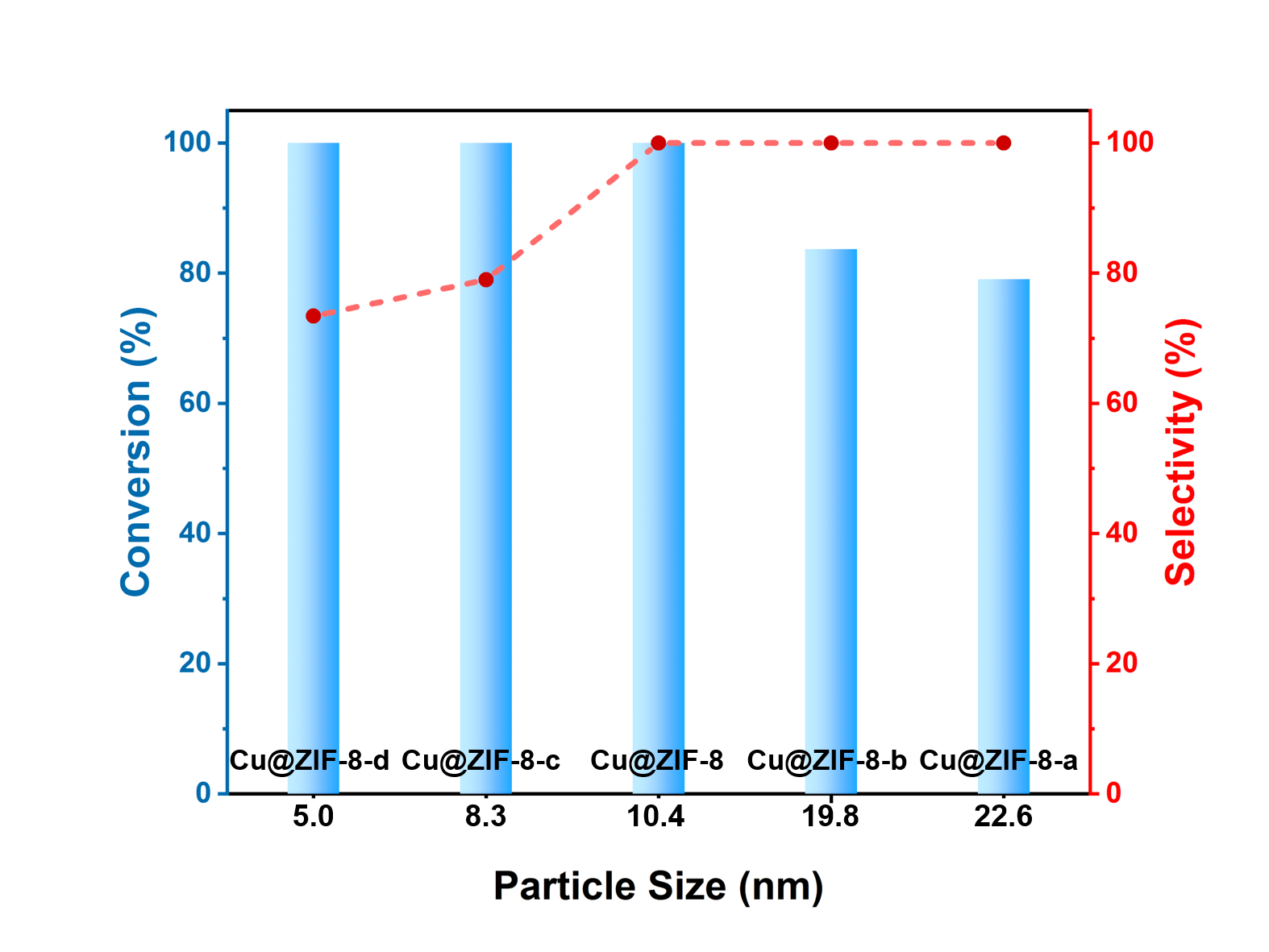


**Figure S76.** Effect of particle size on semihydrogenation of phenylacetylene over Cu@ZIF-8 with different proportions of organic ligands. Reaction conditions: 0.2 mmol phenylacetylene, catalyst (10 mol% based on Cu), 2 mL toluene, 1.5 MPa H_2_, 130 ^o^C, 10 h.

**
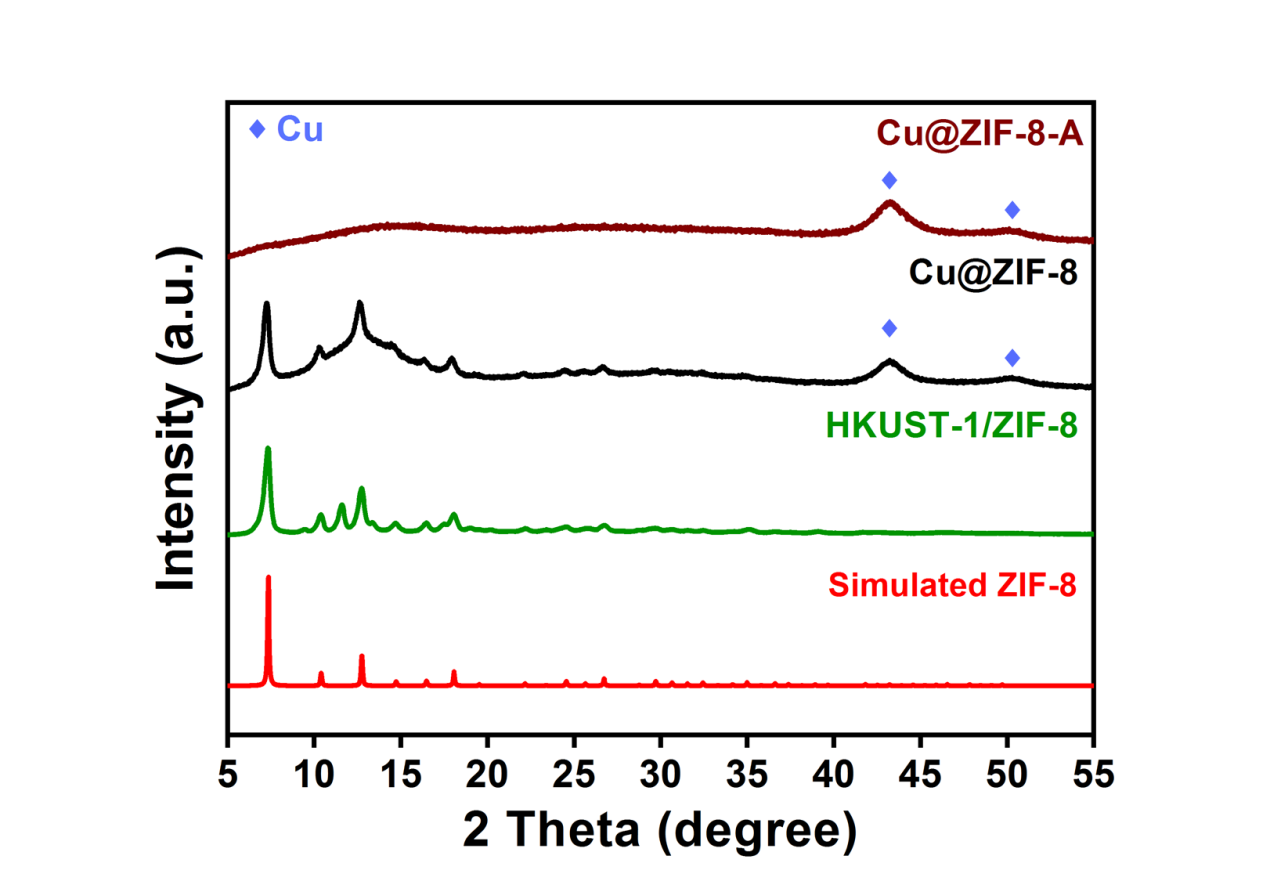
**

**Figure S77.** PXRD pattern of Cu@ZIF-8-A. Cu@ZIF-8-A is the etched product of Cu@ZIF-8, where A represents acetic acid.

**
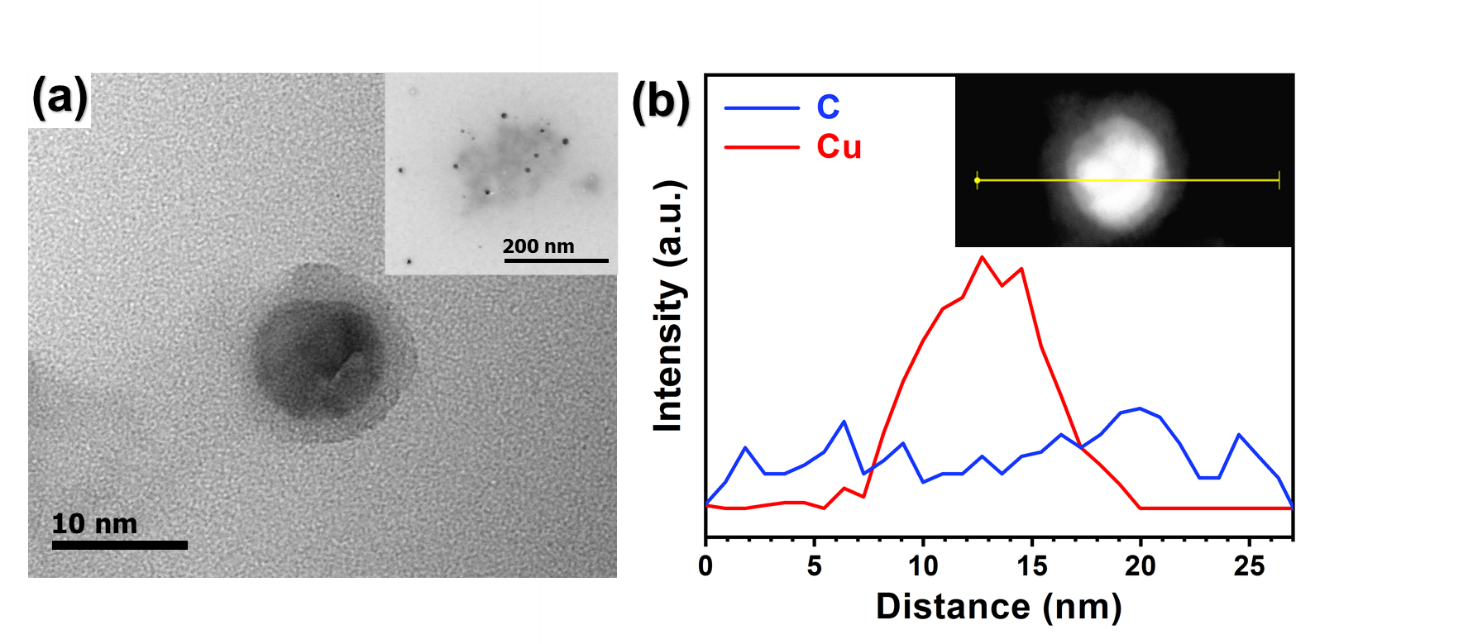
**

**Figure S78.** (a) TEM image and (b) EDX line-scan profile of Cu@ZIF-8-A.

**
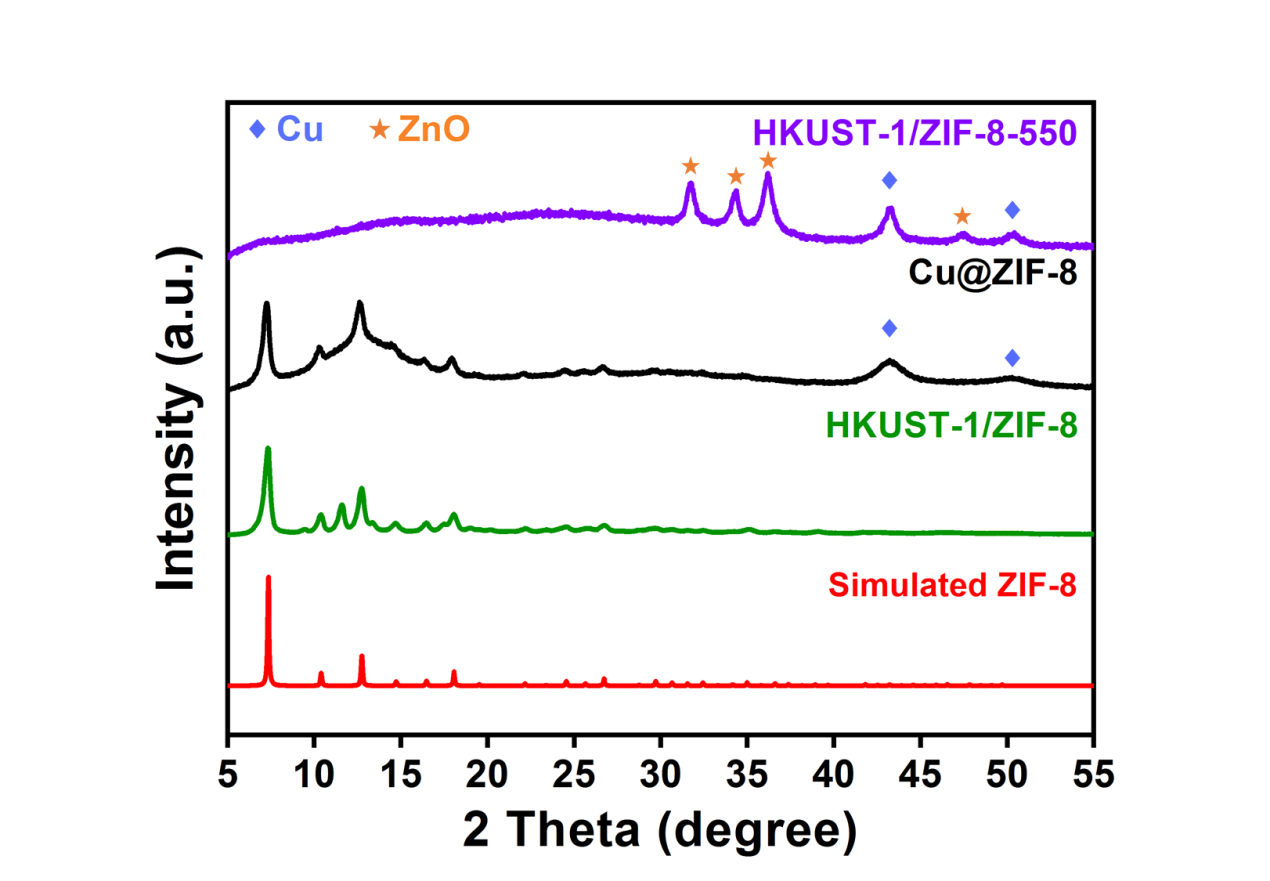
**

**Figure S79.** PXRD pattern of HKUST-1/ZIF-8-550. HKUST-1/ZIF-8-550 is the annealed product of HKUST-1/ZIF-8 at 550 ^o^C.


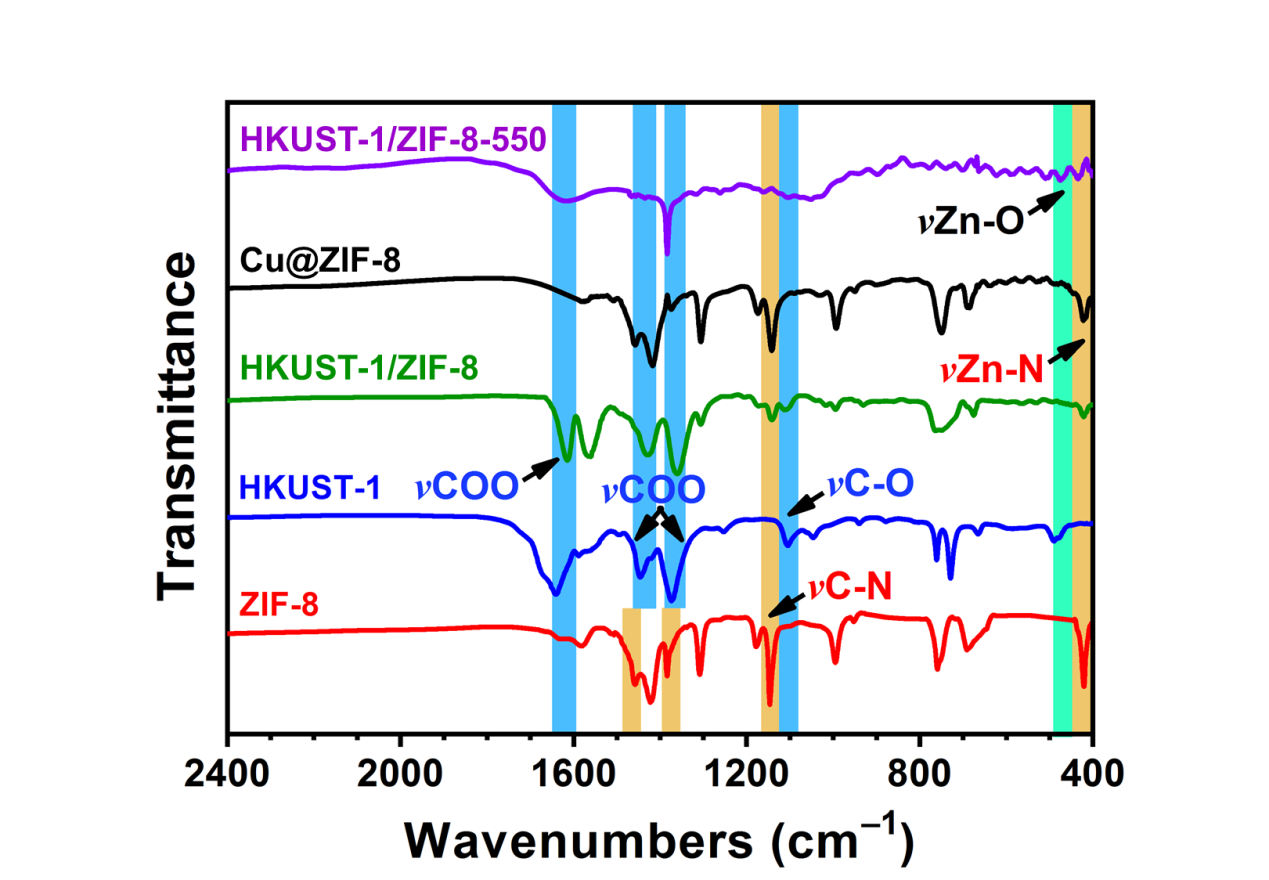


**Figure S80.** FT-IR spectrum of HKUST-1/ZIF-8-550.


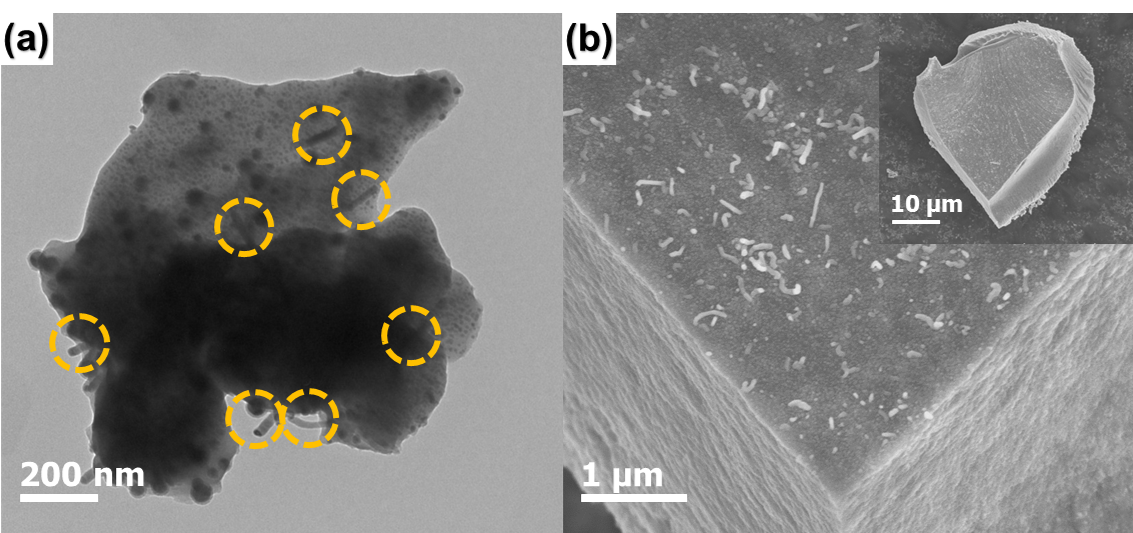


**Figure S81.** (a) TEM image of HKUST-1/ZIF-8-550 (the yellow circles represent the ZnO nanowires derived from decomposition of ZIF-8). (b) SEM image of HKUST-1/ZIF-8-550. The inset shows a low-magnification SEM image.


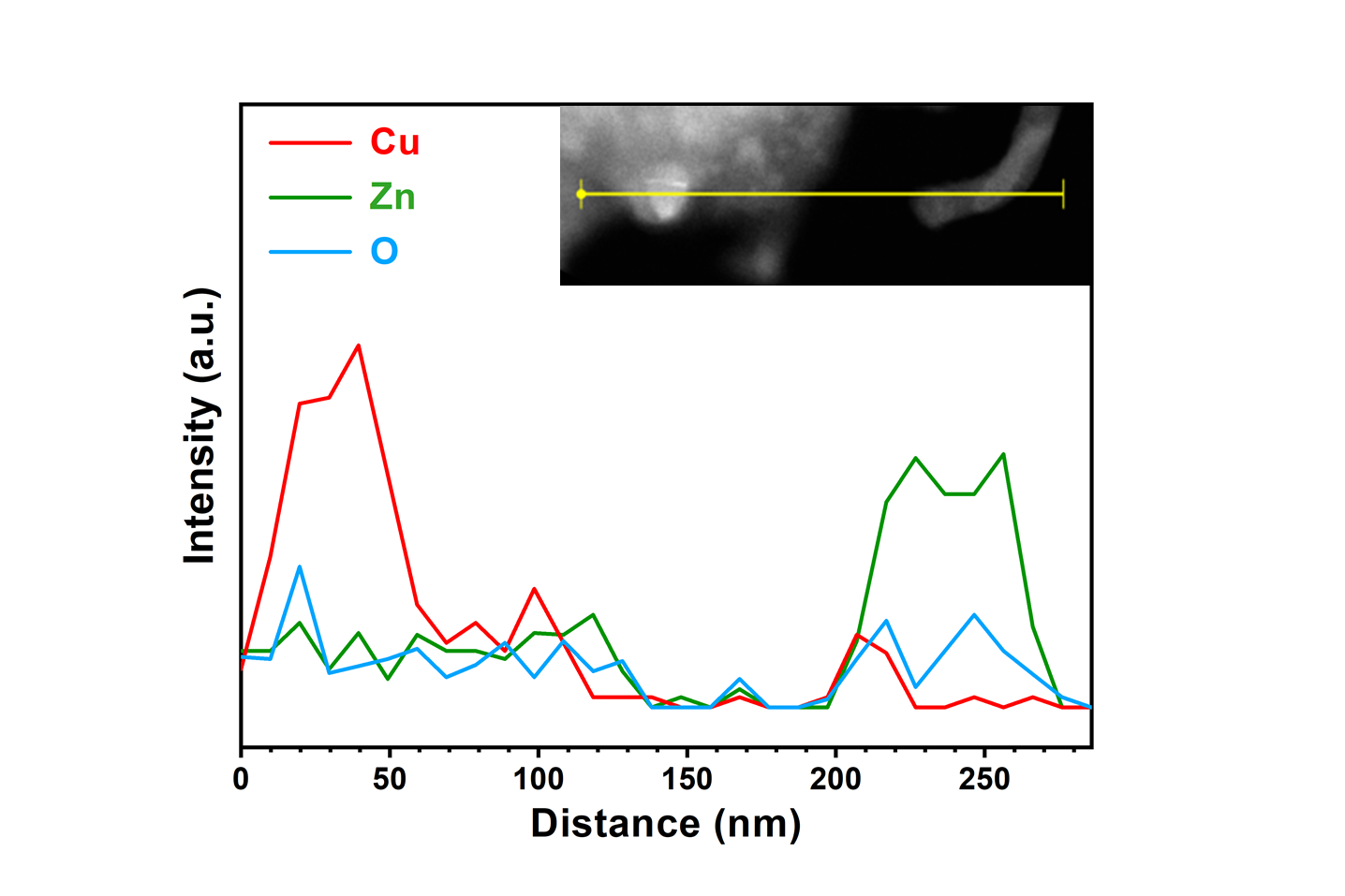


**Figure S82.** EDX line-scan profile of HKUST-1/ZIF-8-550.


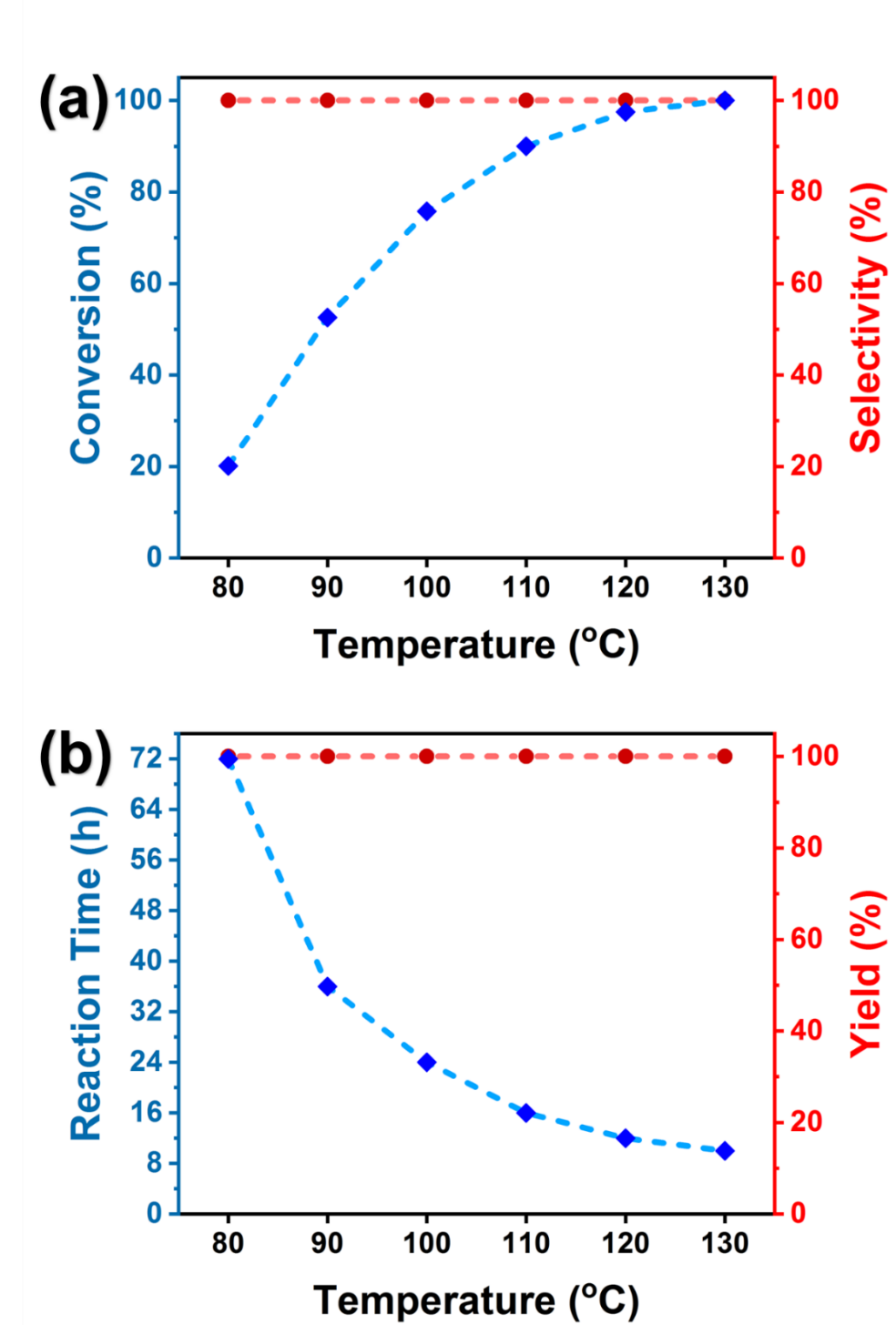


**Figure S83.** Catalytic semihydrogenation of phenylacetylene by Cu@ZIF-8. (a) Phenylacetylene conversion and styrene selectivity versus temperature for phenylacetylene hydrogenation catalyzed by Cu@ZIF-8. (b) Reaction time and styrene yield versus temperature for phenylacetylene hydrogenation catalyzed by Cu@ZIF-8. Reaction conditions: 0.2 mmol phenylacetylene, Cu@ZIF-8 (10 mol% based on Cu), 2 mL toluene, 1.5 MPa H_2_.


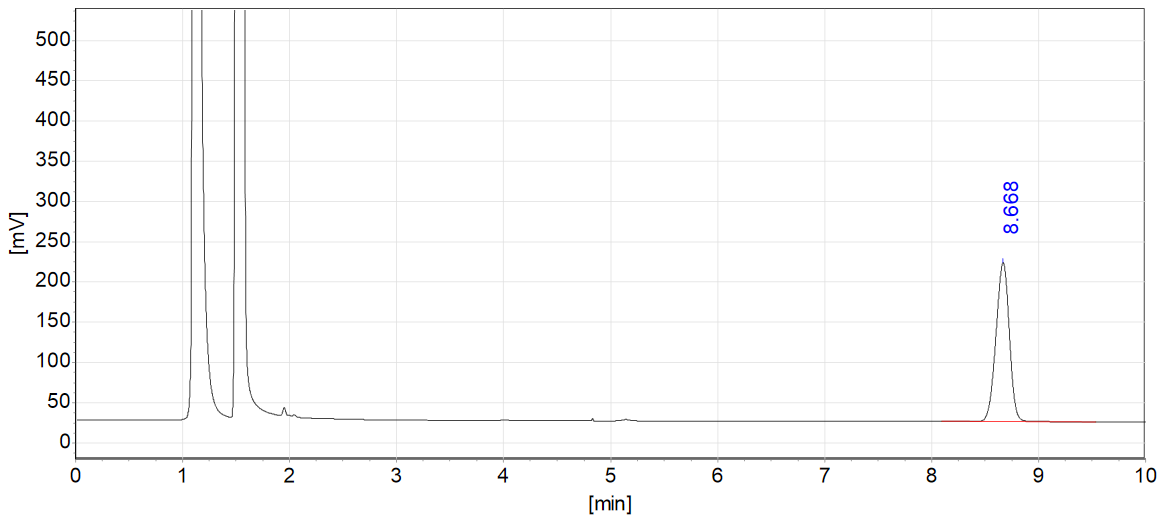


**Figure S84.** GC trace of 3-ethynyltoluene.


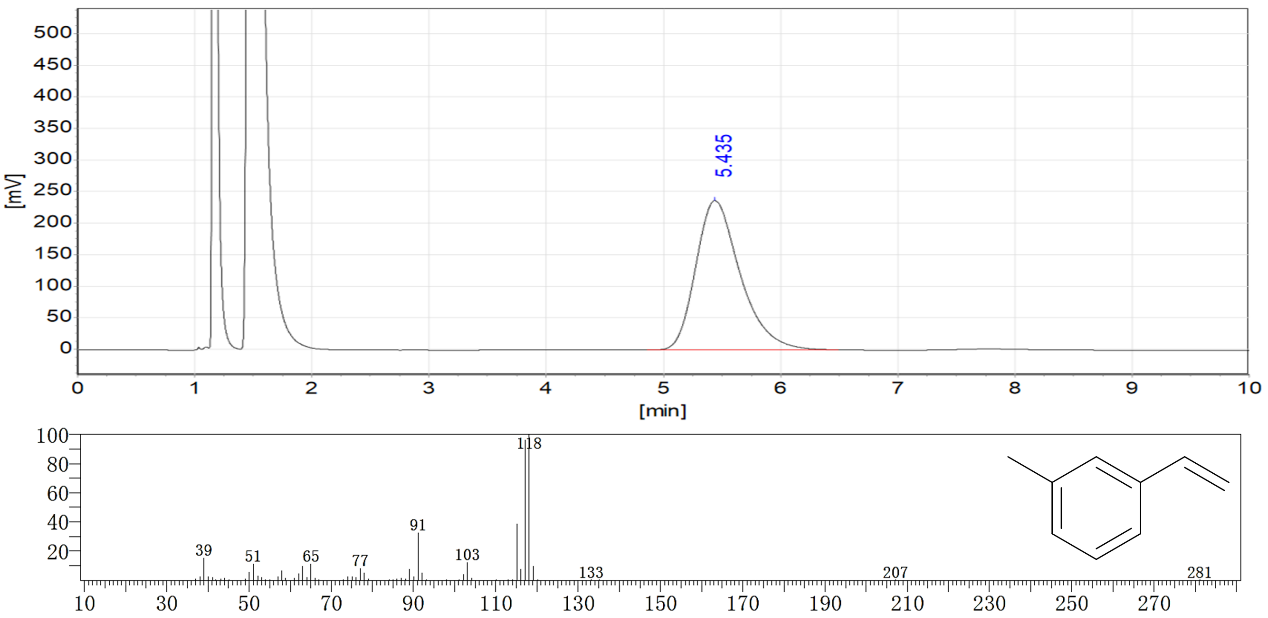


**Figure S85.** GC-MS data for semihydrogenation of 3-ethynyltoluene catalyzed by Cu@ZIF-8.


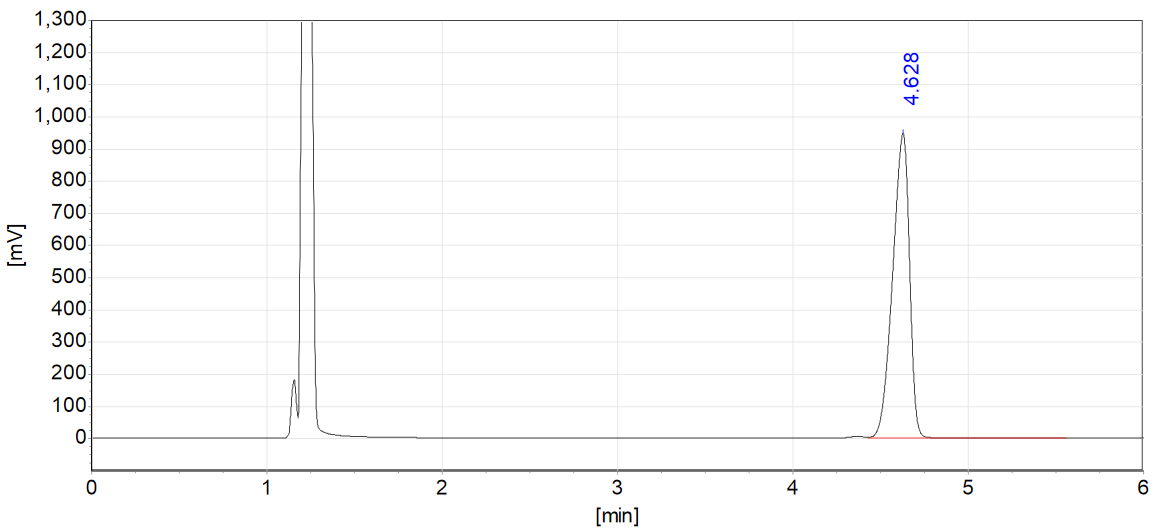


**Figure S86.** GC trace of 1-ethynyl-4-pentylbenzene.


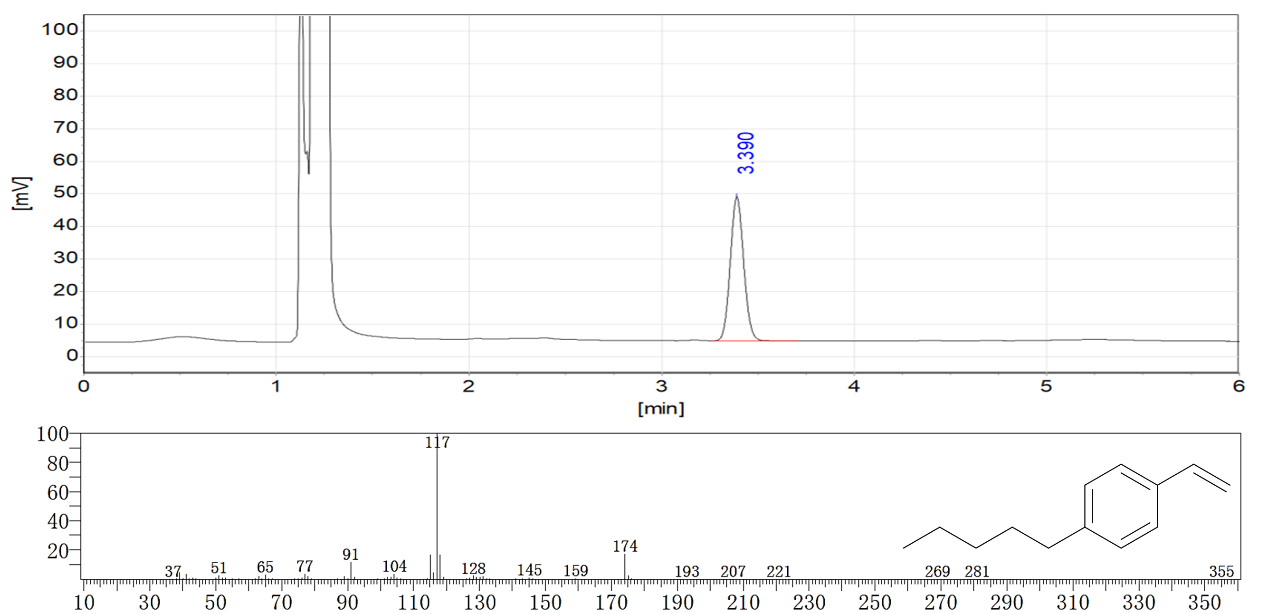


**Figure S87.** GC-MS data for semihydrogenation of 1-ethynyl-4-pentylbenzene catalyzed by Cu@ZIF-8.


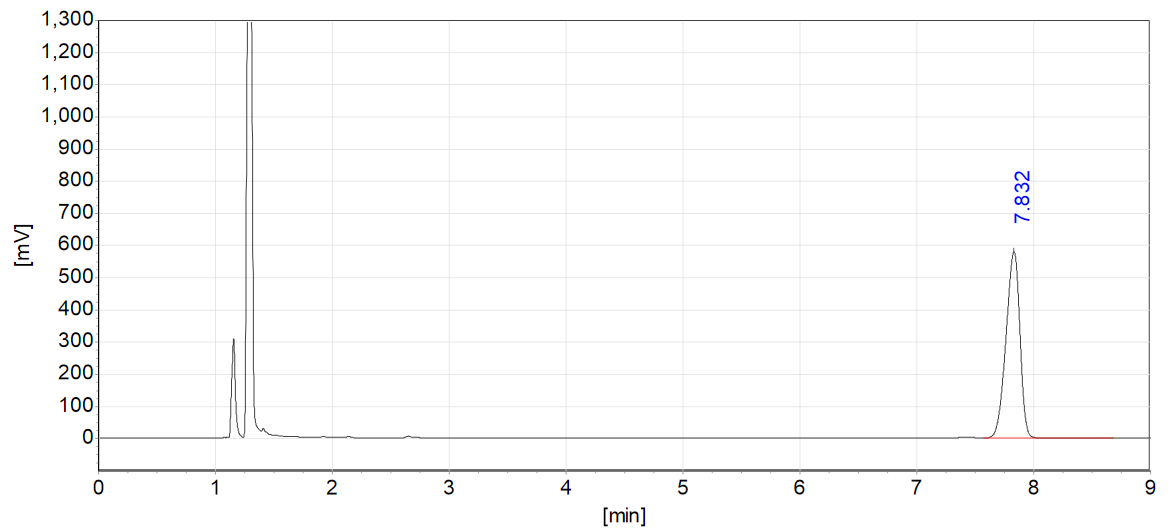


**Figure S88.** GC trace of 3-ethynylanisole.


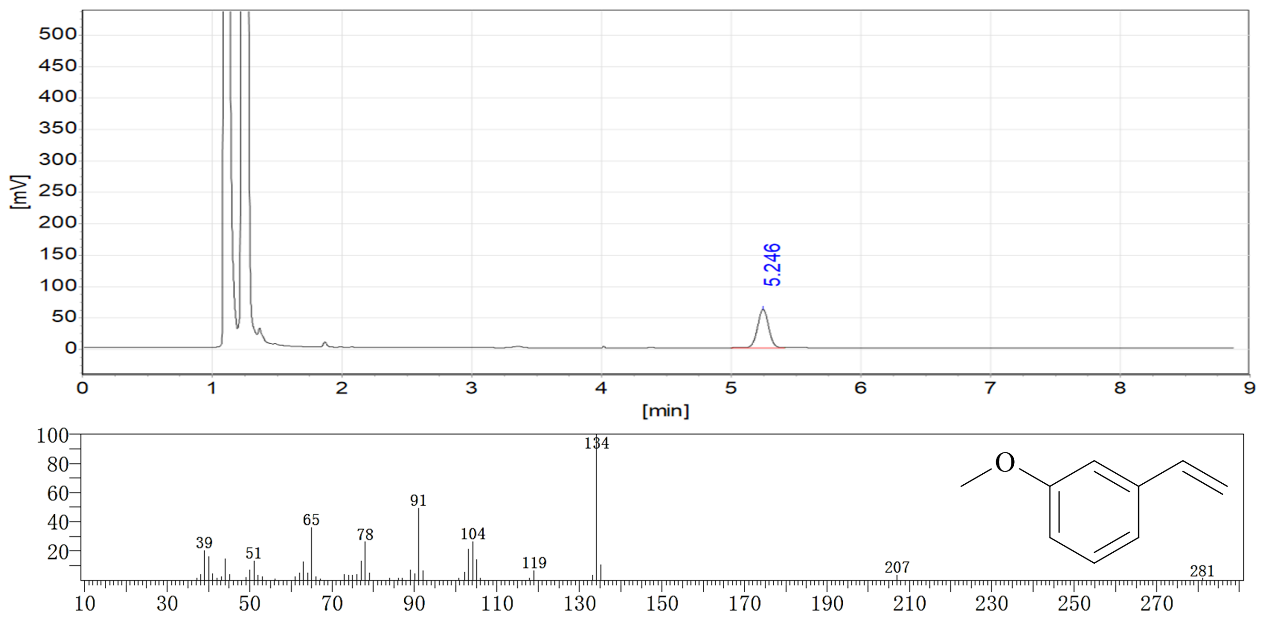


**Figure S89.** GC-MS data for semihydrogenation of 3-ethynylanisole catalyzed by Cu@ZIF-8.


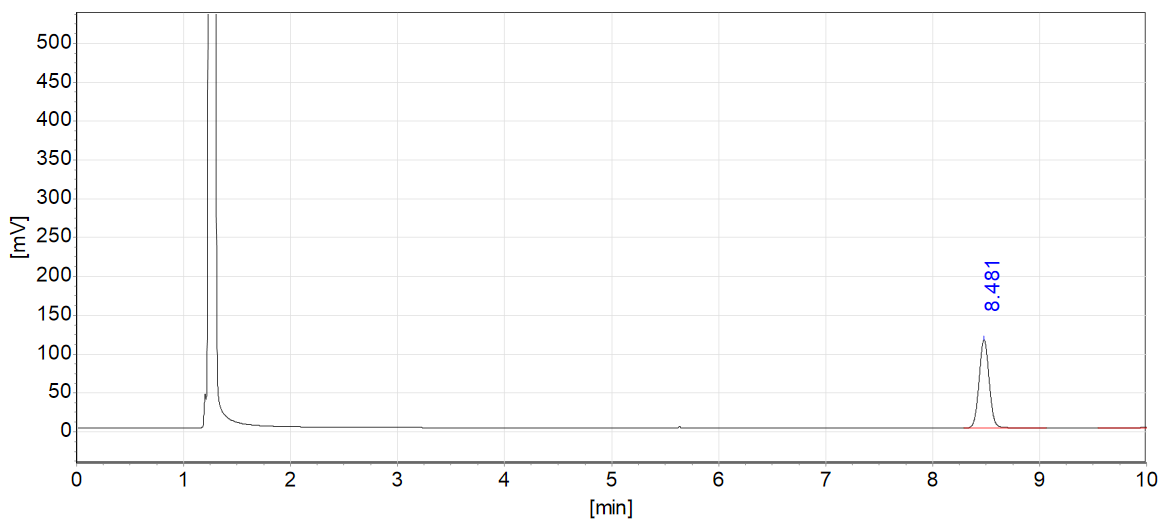


**Figure S90.** GC trace of 4-ethynylaniline.


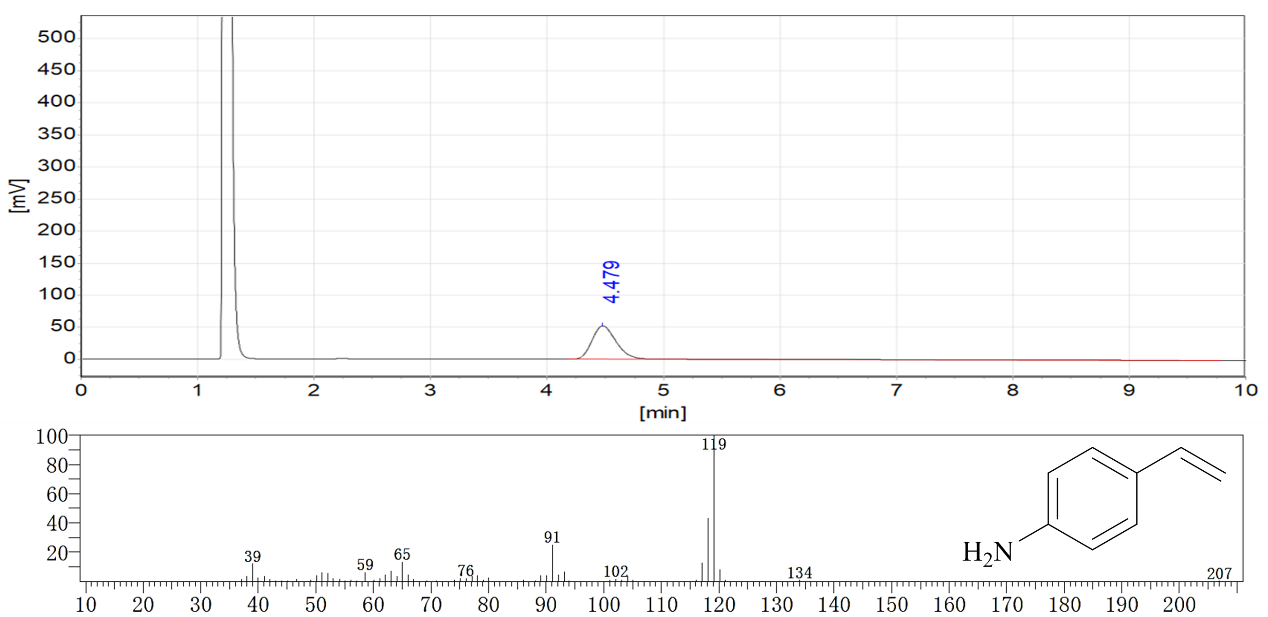


**Figure S91.** GC-MS data for semihydrogenation of 4-ethynylaniline catalyzed by Cu@ZIF-8.


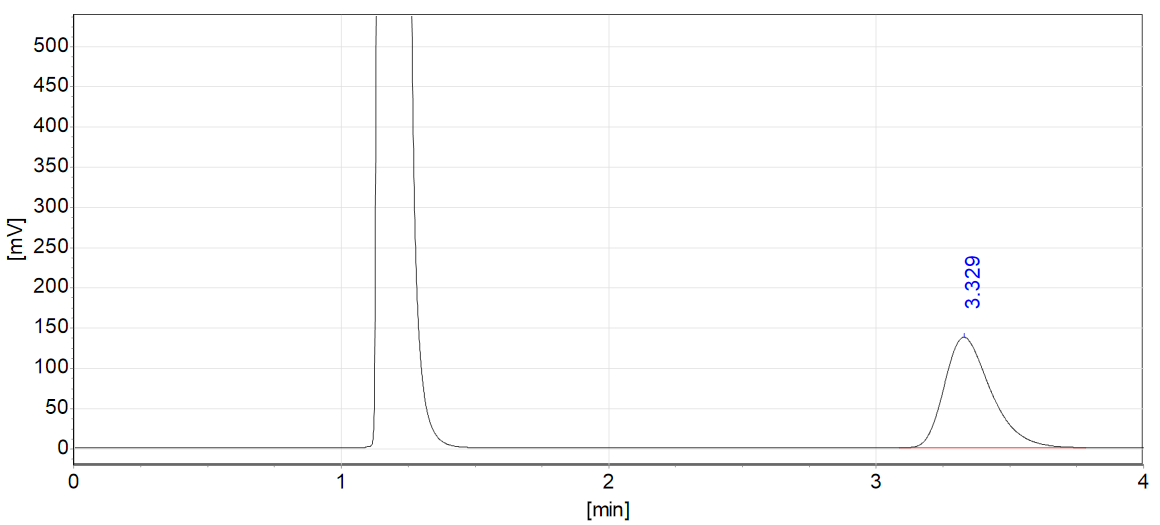


**Figure S92.** GC trace of 1-bromo-4-ethynylbenzene.


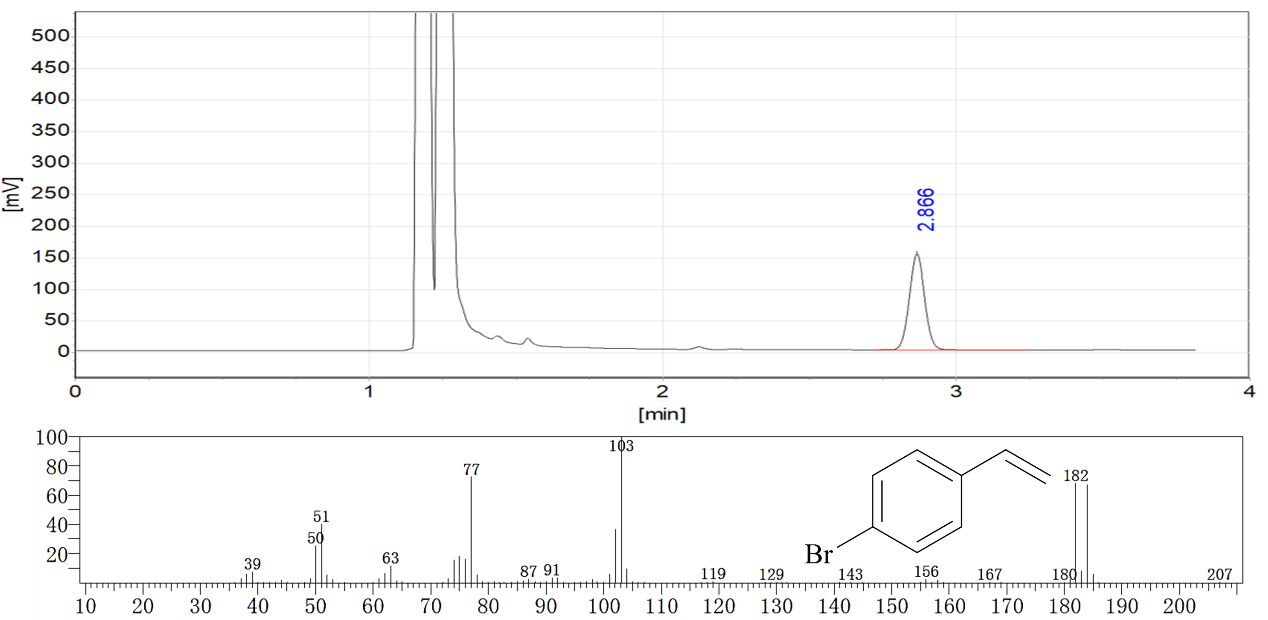


**Figure S93.** GC-MS data for semihydrogenation of 1-bromo-4-ethynylbenzene catalyzed by Cu@ZIF-8.


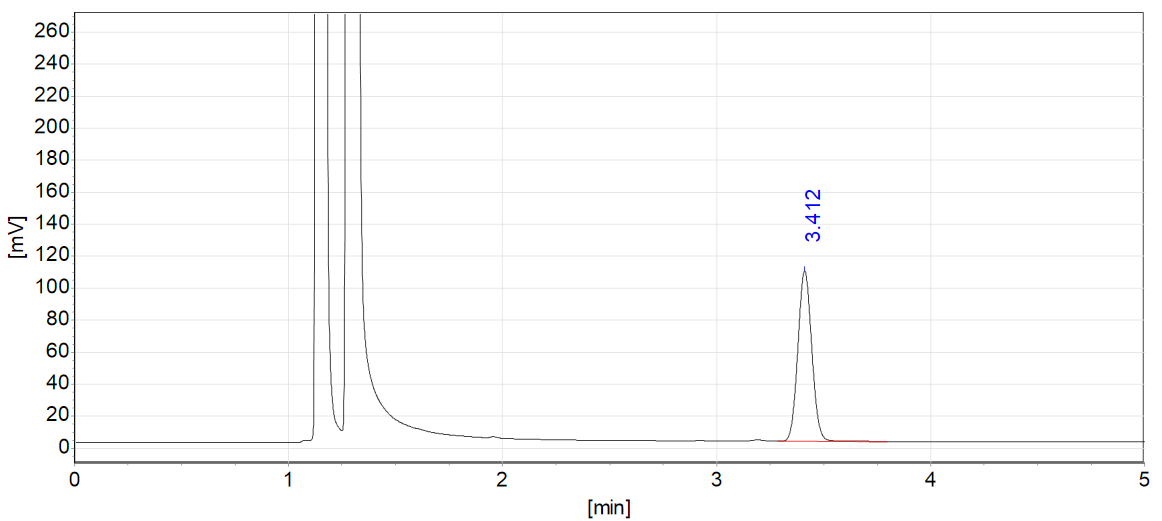


**Figure S94.** GC trace of 3-ethynylpyridine.


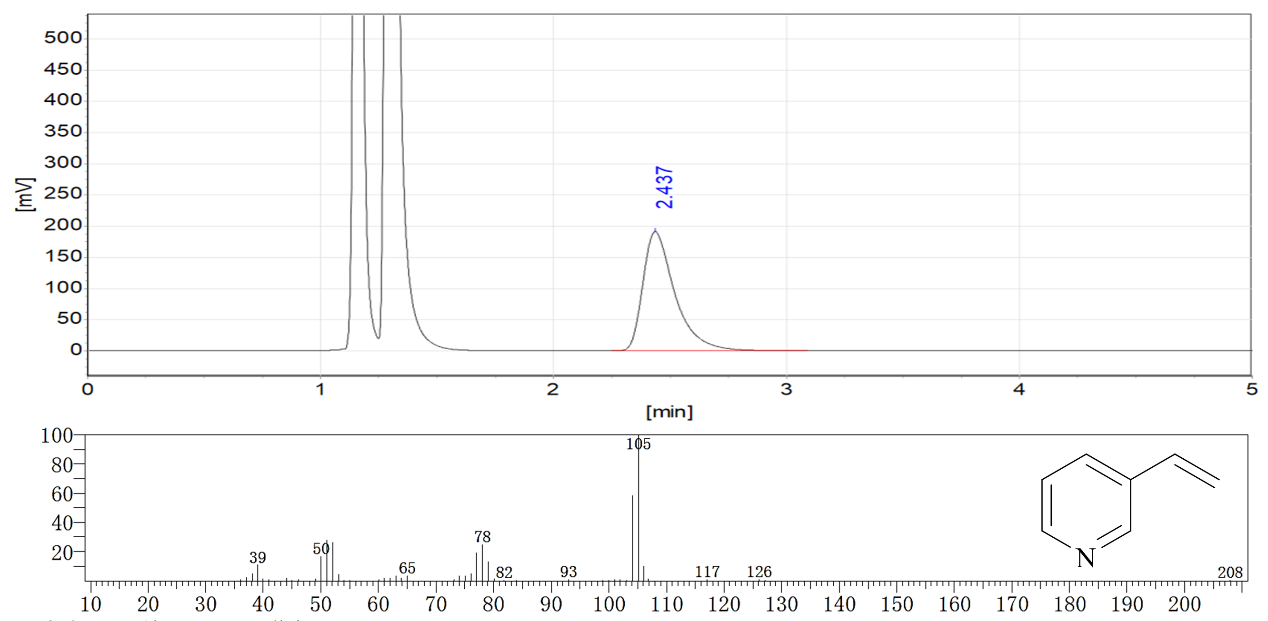


**Figure S95.** GC-MS data for semihydrogenation of 3-ethynylpyridine catalyzed by Cu@ZIF-8.


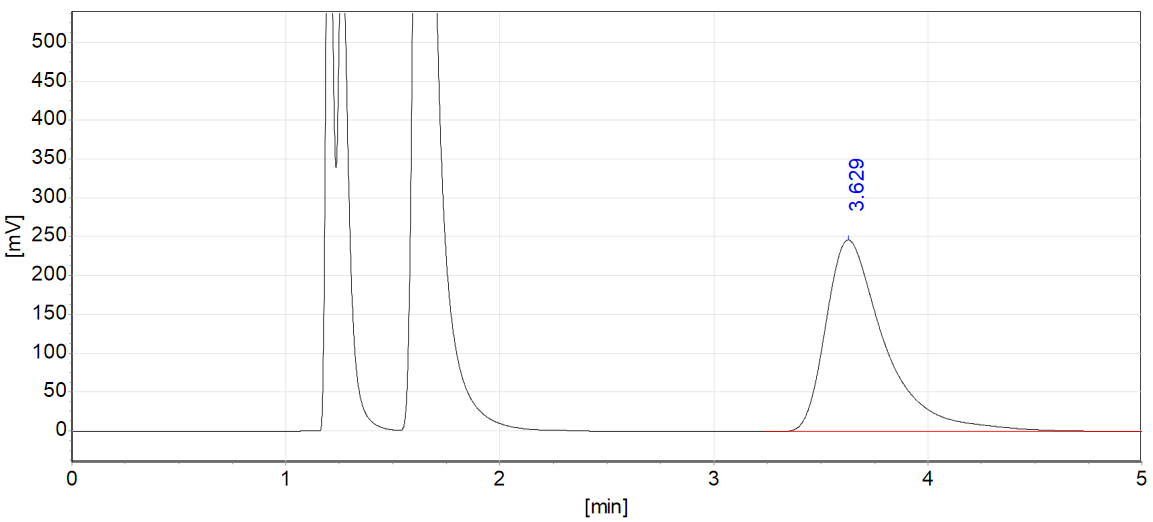


**Figure S96.** GC trace of 1-ethynylcyclohexene.


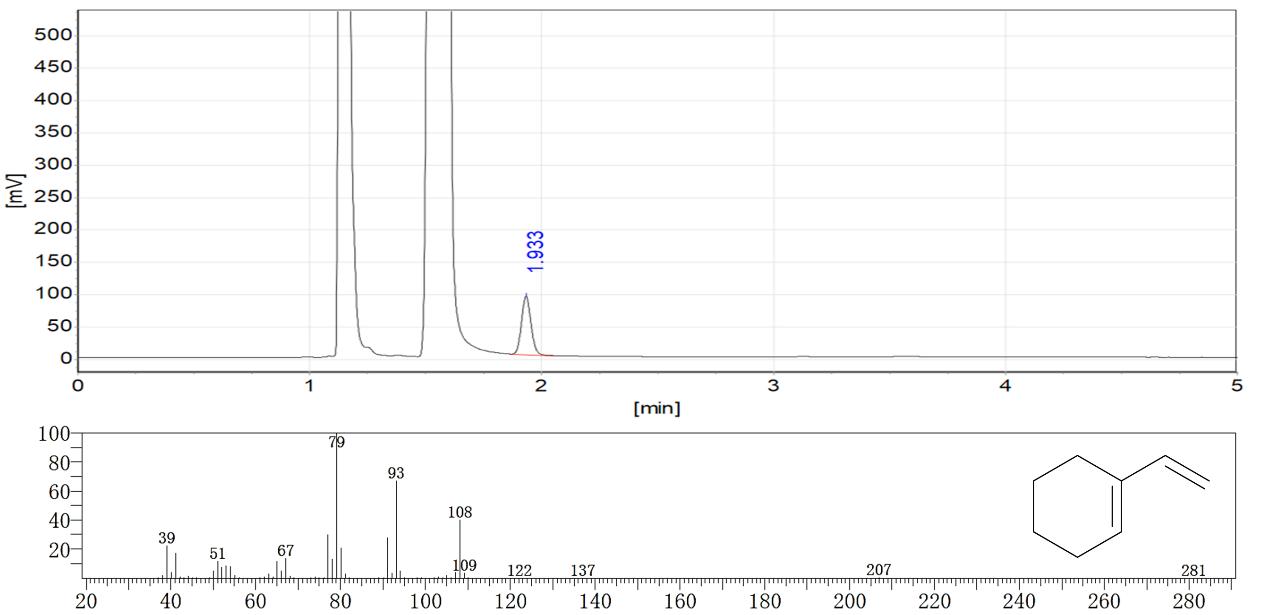


**Figure S97.** GC-MS data for semihydrogenation of 1-ethynylcyclohexene catalyzed by Cu@ZIF-8.


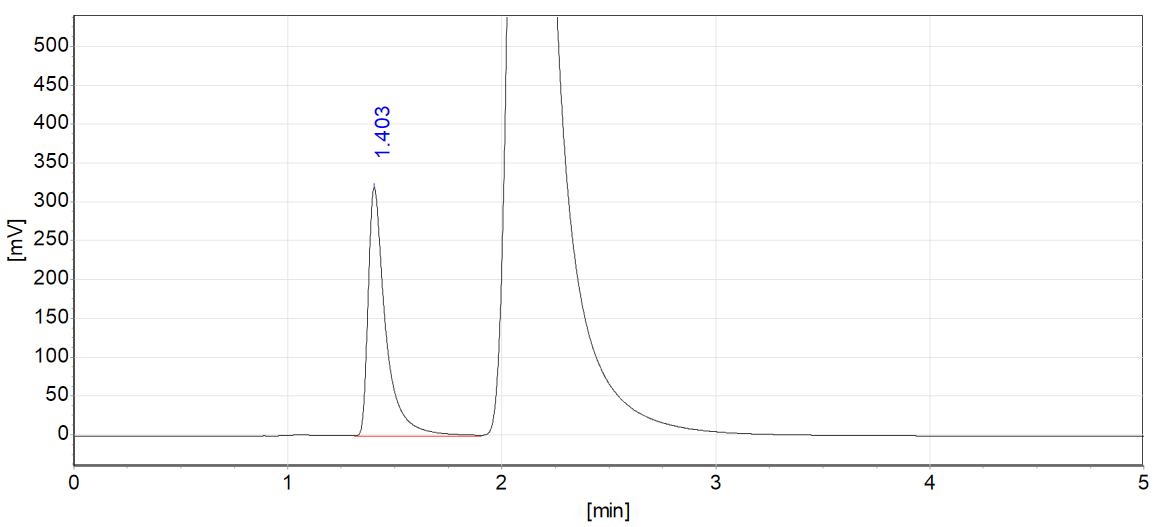


**Figure S98.** GC trace of 1-octyne.


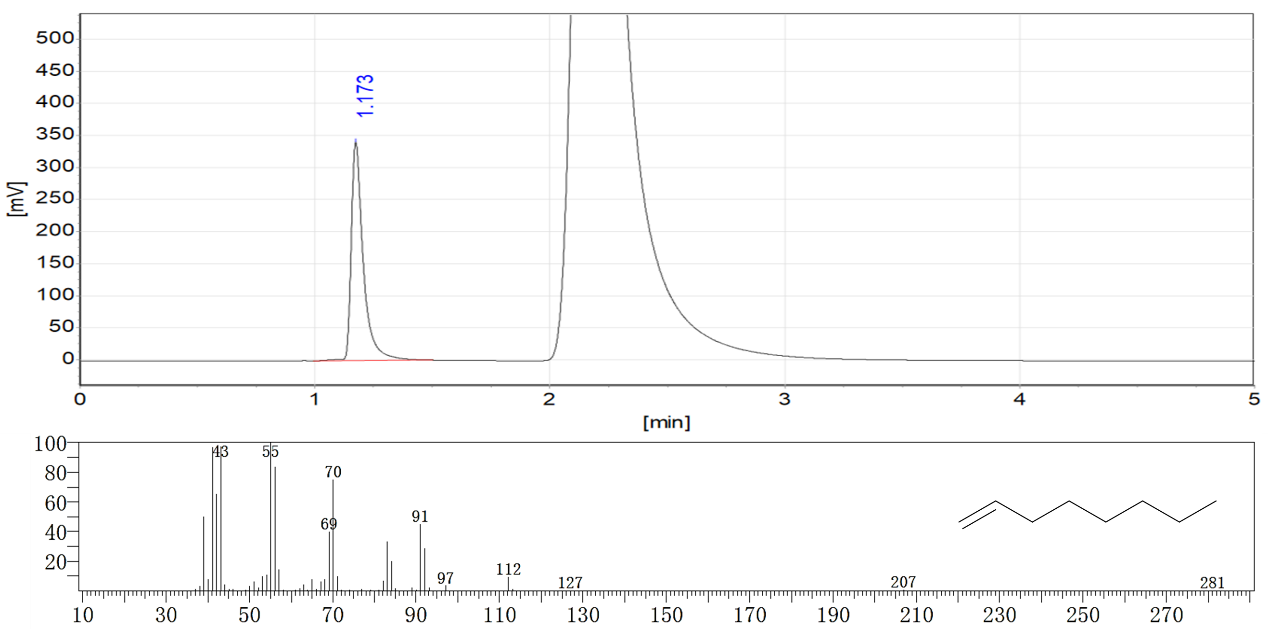


**Figure S99.** GC-MS data for semihydrogenation of 1-octyne catalyzed by Cu@ZIF-8.


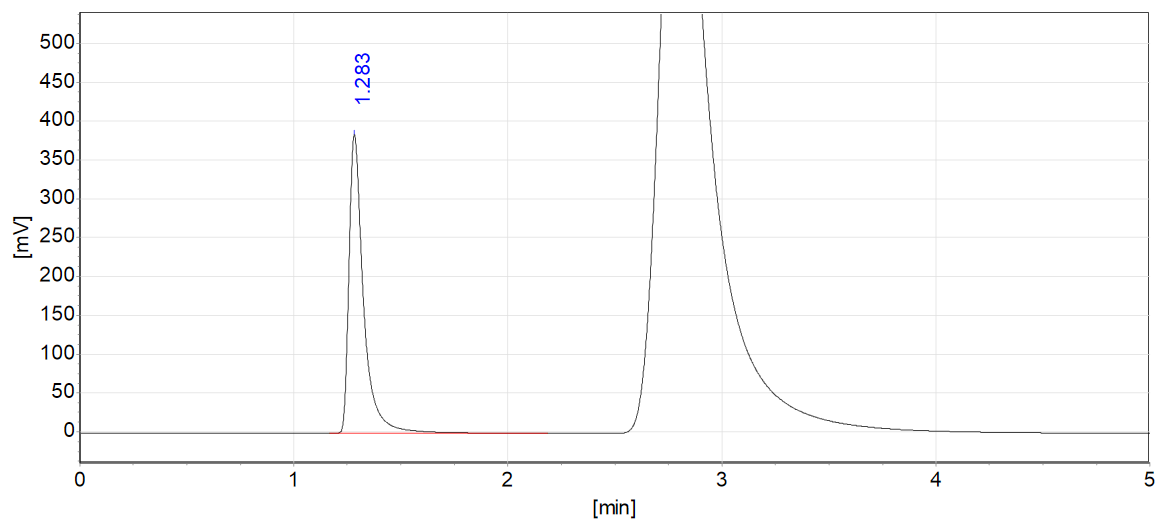


**Figure S100.** GC trace of 1-hexyne.

**Figure S101.** GC-MS data for semihydrogenation of 1-hexyne catalyzed by Cu@ZIF-8.

**Figure S102.** GC trace of 2-methyl-3-butyn-2-ol.

**Figure S103.** GC-MS data for semihydrogenation of 2-methyl-3-butyn-2-ol catalyzed by Cu@ZIF-8.

**Figure S104.** (a) Cu 2p and (b) N 1s high resolution XPS spectra for HKUST-1-350, ZIF-8, Cu@ZIF-8, Cu@ZIF-8-1 and Cu@ZIF-8-2.

**Figure S105.** Phenylacetylene conversion and styrene selectivity versus catalytic cycle for the semihydrogenation of phenylacetylene catalyzed by Cu@ZIF-8. Reaction conditions: 0.2 mmol phenylacetylene, catalyst (20 mol% based on Cu), 2 mL toluene, 1.5 MPa H_2_, 130 ^o^C, 10 h.

**Figure S106.** Comparison of the PXRD patterns of Cu@ZIF-8 before catalysis and after catalysis of the sixth run.

**Figure S107.** TEM image of Cu@ZIF-8 after catalysis of the sixth run. The inset shows a histogram of metal particle size distribution.

**Table S1.** Textual properties of the pristine, etched and annealed samples of HKUST-1/ZIF-8, HKUST-1/ZIF-8-1 and HKUST-1/ZIF-8-2.^[a]^

| Sample | *S*_BET_  (m^2^/g) | *V*_pore_  (cm^3^/g) | *V*_micro_  (cm^3^/g) |
| --- | --- | --- | --- |
| HKUST-1 | 1517.4 | 1.068 | 0.603 |
| ZIF-8 | 1670.7 | 1.531 | 0.706 |
| HKUST-1/ZIF-8 | 968.3 | 1.057 | 0.311 |
| HKUST-1/ZIF-8-1 | 842.1 | 0.624 | 0.342 |
| HKUST-1/ZIF-8-2 | 399.4 | 0.327 | 0.148 |
| HKUST-1/ZIF-8-A | 1207.6 | 1.480 | 0.482 |
| HKUST-1/ZIF-8-B | 1329.8 | 1.197 | 0.488 |
| Cu@ZIF-8 | 1014.1 | 1.061 | 0.372 |
| Cu@ZIF-8-1 | 769.0 | 0.507 | 0.311 |
| Cu@ZIF-8-2 | 34.2 | 0.175 | 0.005 |

^[a]^Calculated from N_2_ physisorption.

**Table S2.** The compositions of different HKUST-1/ZIF-8 dual-MOFs.

| Sample | Cu content  (wt%)^[a]^ | Zn content  (wt%)^[a]^ | H_3_BTC content  (wt%)^[b]^ | 2-Methylimidazole  content  (wt%)^[b]^ |
| --- | --- | --- | --- | --- |
| HKUST-1/ZIF-8 | 10.75 | 19.25 | 17.78 | 49.93 |
| HKUST-1/ZIF-8-1 | 5.22 | 20.58 | 9.28 | 60.53 |
| HKUST-1/ZIF-8-2 | 20.25 | 7.31 | 27.25 | 14.48 |
| HKUST-1/ZIF-8-a | 14.04 | 22.81 | 30.32 | 14.35 |
| HKUST-1/ZIF-8-b | 10.32 | 14.84 | 23.24 | 51.60 |
| HKUST-1/ZIF-8-c | 6.54 | 21.91 | 9.73 | 61.82 |
| HKUST-1/ZIF-8-d | 4.37 | 33.61 | 7.35 | 54.67 |

^[a]^Determined by ICP-OES. ^[b]^Determined by ^1^H NMR.

**Table S3.** The compositions and particle sizes for different annealed samples of HKUST-1/ZIF-8 dual-MOFs.

| Sample | Cu content  (wt%)^[a]^ | Zn content  (wt%)^[a]^ | *d*_Cu_  (nm)^[b]^ |
| --- | --- | --- | --- |
| Cu@ZIF-8 | 15.38 | 27.12 | 10.4 ± 1.6 |
| Cu@ZIF-8-1 | 7.02 | 28.68 | 6.1 ± 1.4 |
| Cu@ZIF-8-2 | 40.40 | 14.80 | 26.4 ± 4.2 |
| Cu@ZIF-8-a | 23.30 | 36.57 | 22.6 ± 4.2 |
| Cu@ZIF-8-b | 15.02 | 21.81 | 19.8 ± 2.4 |
| Cu@ZIF-8-c | 10.70 | 28.02 | 8.3 ± 1.1 |
| Cu@ZIF-8-d | 5.39 | 41.75 | 5.0 ± 0.6 |
| HKUST-1/ZIF-8-550 | 28.26 | 50.60 | 9.2 ± 1.3 |
| Cu@ZIF-8  after the six run catalysis | 20.61 | 36.34 | 11.7 ± 1.5 |

^[a]^Determined by ICP-OES. ^[b]^Calculated based on the TEM analysis results.

**Table S4.** Semihydrogenation of phenylacetylene into styrene by HKUST-1 and ZIF-8, HKUST-1/ZIF-8, Cu/ZIF-8 and various Cu@ZIF-8 composites.^[a]^

| Entry | Catalyst | Conv. (%)^[b]^ | Sel. (%)^[b]^ |
| --- | --- | --- | --- |
| 1 | HKUST-1 | 2.3 | 62.4 |
| 2^[c]^ | ZIF-8 | 7.1 | 89.5 |
| 3 | HKUST-1/ZIF-8 | 6.7 | 78.0 |
| 4 | HKUST-1-350 | 26.2 | 86.9 |
| 5 | Cu@ZIF-8 | >99.9 | >99.9 |
| 6 | Cu@ZIF-8-1 | >99.9 | 83.3 |
| 7 | Cu@ZIF-8-2 | 75.8 | >99.9 |
| 8 | Cu@ZIF-8-a | 79.1 | >99.9 |
| 9 | Cu@ZIF-8-b | 83.7 | >99.9 |
| 10 | Cu@ZIF-8-c | >99.9 | 79.0 |
| 11 | Cu@ZIF-8-d | >99.9 | 73.4 |
| 12 | Cu@ZIF-8-A | 12.2 | >99.9 |
| 13 | HKUST-1/ZIF-8-550 | 85.6 | 48.1 |
| 14 | nHKUST-1@ZIF-8-350 | 9.0 | 76.7 |
| 15 | Cu/ZIF-8 | 96.0 | 45.7 |

^[a]^Reaction conditions: 0.2 mmol phenylacetylene, Cu-based catalyst (10 mol% based on Cu), 2 mL toluene, 1.5 MPa H_2_, 130 ^o^C, 10 h. Major byproduct: ethylbenzene. ^[b]^The phenylacetylene conversion and styrene selectivity were determined by GC-MS. ^[c]^The dosage of ZIF-8 is equal to the content of ZIF-8 in Cu@ZIF-8.

**Table S5.** Representative work for semihydrogenation of phenylacetylene into styrene by noble and non-noble metal-based catalysts in the literature.

| Catalyst | Temp.  (^o^C) | P (H_2_) (MPa) | Time | Conv.  (%) | Sel.  (%) | Ref. |
| --- | --- | --- | --- | --- | --- | --- |
| **Noble metal-based catalysts** | | | | | | |
| Pd@1* | 40 | 0.1 | 1.25 h | >99 | 53 | S3 |
| Pt-Ni-Ag_4.9_ NF@CeO_2_ | 25 | 0.1 | 3.5 h | 97.7 | 86.5 | S4 |
| PdRu | 25 | 0.1 | 4 h | >99 | 90 | S5 |
| Pd/COP | 30 | 1.0 | 5 h | 94.8 | 90.5 | S6 |
| Fe_3_O_4_@ZIF-8/Pd | 40 | 0.1 | 270 min | 99.5 | 90.5 | S7 |
| Pd@Ag-in-UiO-67(30:1) | 25 | 0.1 | 100 min | >99 | 91 | S8 |
| Pd+PEI(L)@HSS_1.35 | 30 | 0.1 | 4.5 h | >99 | 91.3 | S9 |
| Pd@Zn-MOF-74 | 40 | 0.1 | 12 h | >99 | 92 | S10 |
| Pd_5_Au_5_/ZnTi | 45 | 0.1 | 500 min | >99.9 | 92.5 | S11 |
| Pd-Pb alloy octahedral NCs | 25 | 0.1 | 500 min | >99 | 93 | S12 |
| Pd_0.18_Cu_15_/Al_2_O_3_ | 25 | 0.7 | 12 h | 90 | 94 | S13 |
| Pd@mpg-C_3_N_4_ | 30 | 0.1 | 85 min | >99 | 94 | S14 |
| Pd_3_Pb CNCs | 25 | 0.1 | 2 h | >99 | 94.6 | S15 |
| Pd-Ni(1:1)/NC | 50 | 0.1 | 1 h | >99.9 | 94.8 | S16 |
| NU-1000-(py_3_tren)GaRhMe | 100 | 0.5 | 500 min | 99 | 95 | S17 |
| Pd NCs@NCM | 25 | 0.1 | 5 h | 98.9 | 95.3 | S18 |
| Pd@SBA-15 | 30 | 0.1 | 150 min | 94.4 | 95.5 | S19 |
| Pd_1_/Cu(100) | 30 | 0.1 | 2 h | >99.9 | 95.9 | S20 |
| Pd/SPMB-2.7 | 25 | 0.1 | 140 min | >99 | 96 | S21 |
| PdS_0.53_@UiO-66-D | 25 | 0.1 | 2 h | >99.9 | 96.5 | S22 |
| Ru_3_@ZIF-8 | 80 | 4.0 | 5 h | 47 | 97 | S23 |
| PdIn/MgAl_2_O_4_ | 25 | 0.1 | 3.5 h | 92 | 97 | S24 |
| Pd@MPSO/SiO_2_-2 | 30 | 0.1 | 6 h | >99 | 97 | S25 |
| Pd_0.33_Pb_0.67_/C | 30 | 0.1 | 2 h | 97 | 98 | S26 |
| MWCNTs-Fe_3_O_4_-Cu_2_O-Pd | 30 | 0.1 | 6 h | >99 | 98 | S27 |
| Au_>99_Ag_1_NPore | 90 | 0.8 | 24 h | 76 | >99 | S28 |
| Pd@Ag@CeO_2_-1.5 | 40 | 1.5 | 12 h | 97 | >99 | S29 |
| Au_25_(SR)_18_/TiO_2_ | 100 | 2.0 | 20 h | 99.2 | >99.9 | S30 |

| **Non-noble metal-based catalysts** | | | | | | |
| --- | --- | --- | --- | --- | --- | --- |
| Ni-NPs in ionic liquid | 30 | 0.2 | 3 h | >99 | 79 | S31 |
| Fe@C | 300 | 0.1 | 40 min | 99 | 86 | S32 |
| Ni nanosheets | 50 | 0.1 | 5 h | 98 | 89 | S33 |
| pre-NiCu/MMO | 100 | 0.4 | 3 h | 95.8 | 90.3 | S34 |
| NiZn_3_/AlSBA-15 | 40 | 0.1 | 15.5 h | 99.6 | 90.3 | S35 |
| Ni–CNFs(3)/MS | 80 | 0.1 | 3 h | 60 | 91.6 | S36 |
| Ni_5_Mg_4_Ga_3_-500 | 40 | 0.3 | 5 h | 95.1 | 92.2 | S37 |
| Ni_5_Ga_3_/MgAl_2_O_4_ | 40 | 0.5 | 13 h | 98 | 93 | S38 |
| Cu@ZIF-8 | 130 | 1.5 | 10 h | >99.9 | >99.9 | This work |

**Table S6.** Effect of composition and particle size on hydrogenation of phenylacetylene over Cu@ZIF-8.^[a]^

| Entry | Catalyst | Cu content (wt%)^[b]^ | Zn content  (wt%)^[b]^ | *d*_Cu_  (nm)^[c]^ | Conv. (%)^[d]^ | Sel. (%)^[d]^ |
| --- | --- | --- | --- | --- | --- | --- |
| 1 | Cu@ZIF-8 | 15.4 | 27.1 | 10.4 ± 1.6 | >99.9 | >99.9 |
| 2 | Cu@ZIF-8-1 | 7.0 | 28.7 | 6.1 ± 1.4 | >99.9 | 83.3 |
| 3 | Cu@ZIF-8-2 | 40.4 | 14.8 | 26.4 ± 4.2 | 75.8 | >99.9 |
| 4 | Cu@ZIF-8-a | 23.3 | 36.6 | 22.6 ± 4.2 | 79.1 | >99.9 |
| 5 | Cu@ZIF-8-b | 15.0 | 21.8 | 19.8 ± 2.4 | 83.7 | >99.9 |
| 6 | Cu@ZIF-8-c | 10.7 | 28.0 | 8.3 ± 1.1 | >99.9 | 79.0 |
| 7 | Cu@ZIF-8-d | 5.4 | 41.8 | 5.0 ± 0.6 | >99.9 | 73.4 |

^[a]^Reaction conditions: 0.2 mmol phenylacetylene, Cu-based catalyst (10 mol% based on Cu), 2 mL toluene, 1.5 MPa H_2_, 130 ^o^C, 10 h. Major byproduct: ethylbenzene. ^[b]^Determined by ICP-OES. ^[c]^Calculated based on the TEM analysis results. ^[d]^Determined by GC.

**Table S7.** The chemical species adsorbed in HKUST-1/ZIF-8, Cu@ZIF-8 and HKUST-1/ZIF-8-550.^[a]^

| Entry | Catalyst | Adsorbed phenylacetylene (mmol/g)^[b]^ | Adsorbed styrene (mmol/g)^[b]^ |
| --- | --- | --- | --- |
| 1 | HKUST-1/ZIF-8 | 3.58 | 3.26 |
| 2 | Cu@ZIF-8 | 3.46 | 0.66 |
| 3 | HKUST-1/ZIF-8-550 | 0.64 | 1.63 |

^[a]^Absorption conditions: 0.2 mmol phenylacetylene, 0.2 mmol styrene, Cu-based catalyst (10 mg), 2 mL toluene, 1 atm N_2_, 25 ^o^C, 10 h. ^[b]^Determined by GC.

**Table S8.** Semihydrogenation of styrene catalyzed by Cu@ZIF-8 and HKUST-1/ZIF-8-550.^[a]^

| Entry | Catalyst | Substrate | Product | Yield (%)^[b]^ |
| --- | --- | --- | --- | --- |
| 1 | Cu@ZIF-8 |  |  | 0 |
| 2 | HKUST-1/ZIF-8-550 |  |  | 23.2 |

^[a]^Reaction conditions: 0.2 mmol styrene, Cu-based catalyst (10 mol% based on Cu), 2 mL toluene, 1.5 MPa H_2_, 130 ^o^C, 10 h. ^[b]^Determined by GC.

**Table S9.** Hydrogenation of a mixture of phenylacetylene and styrene catalyzed by Cu@ZIF-8.^[a]^

| Entry | Substrate | | | Phenylacetylene conv. (%)^[b]^ | Styrene sel. (%)^[b]^ | Styrene conv. (%)^[b]^ |
| --- | --- | --- | --- | --- | --- | --- |
|  | Phenylacetylene (mmol) | Styrene (mmol) | Toluene  (mL) |  |  |  |
| 1 | 0.2 | 0.2 | 2 | >99.9 | >99.9 | 0 |
| 2 | 0.2 | 2.0 | 5 | >99.9 | >99.9 | 0 |
| 3 | 0.2 | 20.0 | 20 | >99.9 | >99.9 | 0 |

^[a]^Reaction conditions: phenylacetylene, styrene, Cu@ZIF-8 (10 mol% based on Cu), toluene, 1.5 MPa H_2_, 130 ^o^C, 10 h. ^[b]^Determined by GC.

**Supplementary references**

[S1] J. Yang, H. Ye, F. Zhao, and B. Zeng, “A novel Cu_x_O nanoparticles@ZIF-8 composite derived from core–shell metal–organic frameworks for highly selective electrochemical sensing of hydrogen peroxide,” *ACS Applied Materials & Interfaces*, vol. 8, no. 31, pp. 20407–20414, 2016.

[S2] B. Hu, Y. Yin, Z. Zhong, D. Wu, G. Liu, and X. Hong, “Cu@ZIF-8 derived inverse ZnO/Cu catalyst with sub-5 nm ZnO for efficient CO_2_ hydrogenation to methanol,” *Catalysis Science & Technology*, vol. 9, no. 10, pp. 2673–2681, 2019.

[S3] F. Maesing, X. Wang, H. Nuesse, J. Klingauf, and A. Studer, “Facile light-mediated preparation of small polymer-coated palladium-nanoparticles and their application as catalysts for alkyne semi-hydrogenation,” *Chemistry A European Journal*, vol. 23, no. 25, pp. 6014–6018, 2017.

[S4] Y. Long, J. Li, L. Wu, Q. Wang, Y. Liu, X. Wang, S. Song, and H. Zhang, “Construction of trace silver modified core@shell structured Pt-Ni nanoframe@CeO_2_ for semihydrogenation of phenylacetylene,” *Nano Research*, vol. 12, no. 4, pp. 869–875, 2019.

[S5] J. Ge, D. He, L. Bai, R. You, H. Lu, Y. Lin, C. Tan, Y.-B. Kang, B. Xiao, Y. Wu, Z. Deng, W. Huang, H. Zhang, X. Hong, and Y. Li, “Ordered porous Pd octahedra covered with monolayer Ru atoms,” *Journal of the American Chemical Society*, vol. 137, no. 46, pp. 14566–14569, 2015.

[S6] S. Yun, S. Lee, S. Yook, H. A. Patel, C. T. Yavuz, and M. Choi, “Cross-linked “poisonous” polymer: thermochemically stable catalyst support for tuning chemoselectivity,” *ACS Catalysis*, vol. 6, no. 4, pp. 2435–2442, 2016.

[S7] L. Yang, Y. Jin, X. Fang, Z. Cheng, and Z. Zhou, “Magnetically recyclable core-shell structured Pd-based catalysts for semihydrogenation of phenylacetylene,” *Industrial & Engineering Chemistry Research*, vol. 56, no. 48, pp. 14182–14191, 2017.

[S8] L. Chen, B. Huang, X. Qiu, X. Wang, R. Luque, and Y. Li, “Seed-mediated growth of MOF-encapsulated Pd@Ag core-shell nanoparticles: toward advanced room temperature nanocatalysts,” *Chemical Science*, vol. 7, no. 1, pp. 228–233, 2016.

[S9] Y. Kuwahara, H. Kango, and H. Yamashita, “Pd nanoparticles and aminopolymers confined in hollow silica spheres as efficient and reusable heterogeneous catalysts for semihydrogenation of alkynes,” *ACS Catalysis*, vol. 9, no. 3, pp. 1993–2006, 2019.

[S10] H. Q. Wu, L. Huang, J. Q. Li, A. M. Zheng, Y. Tao, L. X. Yang, W. H. Yin, and F. Luo, “Pd@Zn-MOF-74: restricting a guest molecule by the open-metal site in a metal–organic framework for selective semihydrogenation,” *Inorganic Chemistry*, vol. 57, no. 20, pp. 12444–12447, 2018.

[S11] Y. Shen, K. Yin, C. An, and Z. Xiao, “Design of a difunctional Zn-Ti LDHs supported PdAu catalyst for selective hydrogenation of phenylacetylene,” *Applied Surface Science*, vol. 456, no. 31, pp. 1–6, 2018.

[S12] W. Niu, Y. Gao, W. Zhang, N. Yan, and X. Lu, “Pd-Pb alloy nanocrystals with tailored composition for semihydrogenation: taking advantage of catalyst poisoning,” *Angewandte Chemie International Edition*, vol. 54, no. 28, pp. 8271–8274, 2015.

[S13] M. B. Boucher, B. Zugic, G. Cladaras, J. Kammert, M. D. Marcinkowski, T. J. Lawton, E. C. H. Sykes, and M. Flytzani-Stephanopoulos, “Single atom alloy surface analogs in Pd_0.18_Cu_15_ nanoparticles for selective hydrogenation reactions,” *Physical Chemistry Chemical Physics*, vol. 15, no. 29, pp. 12187–12196, 2013.

[S14] D. Deng, Y. Yang, Y. Gong, Y. Li, X. Xu, and Y. Wang, “Palladium nanoparticles supported on mpg-C_3_N_4_ as active catalyst for semihydrogenation of phenylacetylene under mild conditions,” *Green Chemistry*, vol. 15, no. 9, pp. 2525–2531, 2013.

[S15] J. Zhang, W. Xu, L. Xu, Q. Shao, and X. Huang, “Concavity tuning of intermetallic Pd-Pb nanocubes for selective semihydrogenation catalysis,” *Chemistry of Materials*, vol. 30, no. 18, pp. 6338–6345, 2018.

[S16] W. Zhang, W. Wu, Y. Long, J. Qin, F. Wang, and J. Ma, “Promoting role of iron series elements modification on palladium/nitrogen doped carbon for the semihydrogenation of phenylacetylene,” *ChemCatChem*, vol. 11, no. 5, pp. 1510–1517, 2019.

[S17] S. P. Desai, J. Ye, J. Zheng, M. S. Ferrandon, T. E. Webber, A. E. Platero-Prats, J. Duan, P. Garcia-Holley, D. M. Camaioni, K. W. Chapman, M. Delferro, O. K. Farha, J. L. Fulton, L. Gagliardi, J. A. Lercher, R. L. Penn, A. Stein, and C. C. Lu, “Well-defined rhodium–gallium catalytic sites in a metal–organic framework: promoter-controlled selectivity in alkyne semihydrogenation to *E*-alkenes,” *Journal of the American Chemical Society*, vol. 140, no. 45, pp. 15309–15318, 2018.

[S18] X. Li, Y. Pan, H. Yi, J. Hu, D. Yang, F. Lv, W. Li, J. Zhou, X. Wu, A. Lei, and L. Zhang, “Mott–schottky effect leads to alkyne semihydrogenation over Pd-nanocube@N-doped carbon,” *ACS Catalysis*, vol. 9, no. 5, pp. 4632–4641, 2019.

[S19] D. Bhuyan, K. Selvaraj, and L. Saikia, “Pd@SBA-15 nanocomposite catalyst: synthesis and efficient solvent-free semihydrogenation of phenylacetylene under mild conditions,” *Microporous Mesoporous Mater*, vol. 241, pp. 266–273, 2017.

[S20] L. Jiang, K. Liu, S.-F. Hung, L. Zhou, R. Qin, Q. Zhang, P. Liu, L. Gu, H. M. Chen, G. Fu, and N. Zheng, “Facet engineering accelerates spillover hydrogenation on highly diluted metal nanocatalysts,” *Nature Nanotechnology*, vol. 15, no. 10, pp. 848–853, 2020.

[S21] Y. Zhang, X. Wen, Y. Shi, R. Yue, L. Bai, Q. Liu, and X. Ba, “Sulfur-containing polymer as a platform for synthesis of size-controlled Pd nanoparticles for selective semihydrogenation of alkynes,” *Industrial & Engineering Chemistry Research*, vol. 58, no. 3, pp. 1142–1149, 2019.

[S22] M.-J. Dong, X. Wang, and C.-D. Wu, “Creation of redox-active PdS_x_ nanoparticles inside the defect pores of MOF UiO-66 with unique semihydrogenation catalytic properties,” *Advanced Functional Materials*, vol. 30, no. 7, article 1908519, 2019.

[S23] S. Ji, Y. Chen, S. Zhao, W. Chen, L. Shi, Y. Wang, J. Dong, Z. Li, F. Li, C. Chen, Q. Peng, J. Li, D. Wang, and Y. Li, “Atomically dispersed ruthenium species inside metal−organic frameworks: combining the high activity of atomic sites and the molecular sieving effect of MOFs,” *Angewandte Chemie International Edition*, vol. 58, no. 13, pp. 4271–4275, 2019.

[S24] Q. Feng, S. Zhao, Y. Wang, J. Dong, W. Chen, D. He, D. Wang, J. Yang, Y. Zhu, H. Zhu, L. Gu, Z. Li, Y. Liu, R. Yu, J. Li, and Y. Li, “Isolated single-atom Pd sites in intermetallic nanostructures: high catalytic selectivity for semihydrogenation of alkynes,” *Journal of the American Chemical Society*, vol. 139, no. 21, pp. 7294–7301, 2017.

[S25] T. Mitsudome, Y. Takahashi, S. Ichikawa, T. Mizugaki, K. Jitsukawa, and K. Kaneda, “Metal–ligand core–shell nanocomposite catalysts for the selective semihydrogenation of alkynes,” *Angewandte Chemie International Edition*, vol. 52, no. 5, pp. 1481–1485, 2013.

[S26] J. Liu, Y. Zhu, C. Liu, X. Wang, C. Cao, and W. Song, “Excellent selectivity with high conversion in semi-hydrogenation of alkynes using Pd-based bimetallic catalysts,” *ChemCatChem*, vol. 9, no. 21, pp. 4053–4057, 2017.

[S27] S. Yang, C. Cao, L. Peng, J. Zhang, B. Han, and W. Song, “A Pd-Cu_2_O nanocomposite as an effective synergistic catalyst for selective semi-hydrogenation of the terminal alkynes only,” *Chemical Communications*, vol. 52, no. 18, pp. 3627–3630, 2016.

[S28] B. S. Takale, X. Feng, Y. Lu, M. Bao, T. Jin, T. Minato, and Y. Yamamoto, “Unsupported nanoporous gold catalyst for chemoselective hydrogenation reactions under low pressure: effect of residual silver on the reaction,” *Journal of the American Chemical Society*, vol. 138, no. 32, pp. 10356–10364, 2016.

[S29] S. Song, K. Li, J. Pan, F. Wang, J. Li, J. Feng, S. Yao, X. Ge, X. Wang, and H. Zhang, “Achieving the trade-off between selectivity and activity in semihydrogenation of alkynes by fabrication of (asymmetrical Pd@Ag core)@(CeO_2_ shell) nanocatalysts via autoredox reaction,” *Advanced Materials*, vol. 29, no. 8, article 1605332, 2017.

[S30] G. Li, and R. Jin, “Gold nanocluster-catalyzed semihydrogenation: a unique activation pathway for terminal alkynes,” *Journal of the American Chemical Society*, vol. 136, no. 32, pp. 11347–11354, 2014.

[S31] H. Konnerth, and M. H. G. Prechtl, “Selective partial hydrogenation of alkynes to (*Z*)-alkenes with ionic liquid-doped nickel nanocatalysts at near ambient conditions,” *Chemical Communications*, vol. 52, no. 58, pp. 9129–9132, 2016.

[S32] A. V. Erokhin, E. S. Lokteva, A. Y. Yermakov, D. W. Boukhvalov, K. I. Maslakov, E. V. Golubina, and M. A. Uimin, “Phenylacetylene hydrogenation on Fe@C and Ni@C core–shell nanoparticles: about intrinsic activity of graphene-like carbon layer in H_2_ activation,” *Carbon*, vol. 74, pp. 291–301, 2014.

[S33] J.-W. Yu, X.-Y. Wang, C.-Y. Yuan, W.-Z. Li, Y.-H. Wang, and Y.-W. Zhang, “Synthesis of ultrathin Ni nanosheets for semihydrogenation of phenylacetylene to styrene under mild conditions,” *Nanoscale*, vol. 10, no. 15, pp. 6936–6944, 2018.

[S34] Y. Liu, J. Zhao, J. Feng, Y. He, Y. Du, and D. Li, “Layered double hydroxide-derived Ni-Cu nanoalloy catalysts for semi-hydrogenation of alkynes: improvement of selectivity and anti-coking ability via alloying of Ni and Cu,” *Journal of Catalysis*, vol. 359, pp. 251–260, 2018.

[S35] L. Yang, S. Yu, C. Peng, X. Fang, Z. Cheng, and Z. Zhou, “Semihydrogenation of phenylacetylene over nonprecious Ni-based catalysts supported on AlSBA-15,” *Journal of Catalysis*, vol. 370, pp. 310–320, 2019.

[S36] W. Donphai, T. Kamegawa, M. Chareonpanich, and H. Yamashita, “Reactivity of Ni–carbon nanofibers/mesocellular silica composite catalyst for phenylacetylene hydrogenation,” *Industrial & Engineering Chemistry Research*, vol. 53, no. 24, pp. 10105–10111, 2014.

[S37] C. Li, Y. Chen, S. Zhang, J. Zhou, F. Wang, S. He, M. Wei, D. G. Evans, and X. Duan, “Nickel-gallium intermetallic nanocrystal catalysts in the semihydrogenation of phenylacetylene,” *ChemCatChem*, vol. 6, no. 3, pp. 824–831, 2014.

[S38] Y. Liu, X. Liu, Q. Feng, D. He, L. Zhang, C. Lian, R. Shen, G. Zhao, Y. Ji, D. Wang, G. Zhou, and Y. Li, “Intermetallic Ni_x_M_y_ (M = Ga and Sn) nanocrystals: a non-precious metal catalyst for semi-hydrogenation of alkynes,” *Advanced Materials*, vol. 28, no. 23, pp. 4747–4754, 2016.
